# Oxidative damage to epigenetically methylated sites affects DNA stability, dynamics and enzymatic demethylation

**DOI:** 10.1093/nar/gky893

**Published:** 2018-10-05

**Authors:** David R Gruber, Joanna J Toner, Heather L Miears, Andrey V Shernyukov, Alexey S Kiryutin, Alexander A Lomzov, Anton V Endutkin, Inga R Grin, Darya V Petrova, Maxim S Kupryushkin, Alexandra V Yurkovskaya, Eric C Johnson, Mark Okon, Elena G Bagryanskaya, Dmitry O Zharkov, Serge L Smirnov

**Affiliations:** 1 Chemistry Department, Western Washington University, 516 High St., Bellingham, WA 98225-9150, USA; 2 N. N. Vorozhtsov Novosibirsk Institute of Organic Chemistry SB RAS, 9 Lavrentieva Ave., Novosibirsk 630090, Russia; 3 Novosibirsk State University, 2 Pirogova St., Novosibirsk 630090, Russia; 4 SB RAS International Tomography Center, 3a Institutskaya St., Novosibirsk 630090, Russia; 5 SB RAS Institute of Chemical Biology and Fundamental Medicine, 8 Lavrentieva Ave., Novosibirsk 630090, Russia; 6 Bruker Biospin, 61 Daggett Dr, San Jose, CA 95134, USA; 7 Department of Biochemistry and Molecular Biology, Department of Chemistry, and Michael Smith Laboratories, University of British Columbia, Vancouver BC, V6T 1Z3, Canada

## Abstract

DNA damage can affect various regulatory elements of the genome, with the consequences for DNA structure, dynamics, and interaction with proteins remaining largely unexplored. We used solution NMR spectroscopy, restrained and free molecular dynamics to obtain the structures and investigate dominant motions for a set of DNA duplexes containing CpG sites permuted with combinations of 5-methylcytosine (mC), the primary epigenetic base, and 8-oxoguanine (oxoG), an abundant DNA lesion. Guanine oxidation significantly changed the motion in both hemimethylated and fully methylated DNA, increased base pair breathing, induced BI→BII transition in the backbone 3′ to the oxoG and reduced the variability of shift and tilt helical parameters. UV melting experiments corroborated the NMR and molecular dynamics results, showing significant destabilization of all methylated contexts by oxoG. Notably, some dynamic and thermodynamic effects were not additive in the fully methylated oxidized CpG, indicating that the introduced modifications interact with each other. Finally, we show that the presence of oxoG biases the recognition of methylated CpG dinucleotides by ROS1, a plant enzyme involved in epigenetic DNA demethylation, in favor of the oxidized DNA strand. Thus, the conformational and dynamic effects of spurious DNA oxidation in the regulatory CpG dinucleotide can have far-reaching biological consequences.

## INTRODUCTION

**Figure 1.**
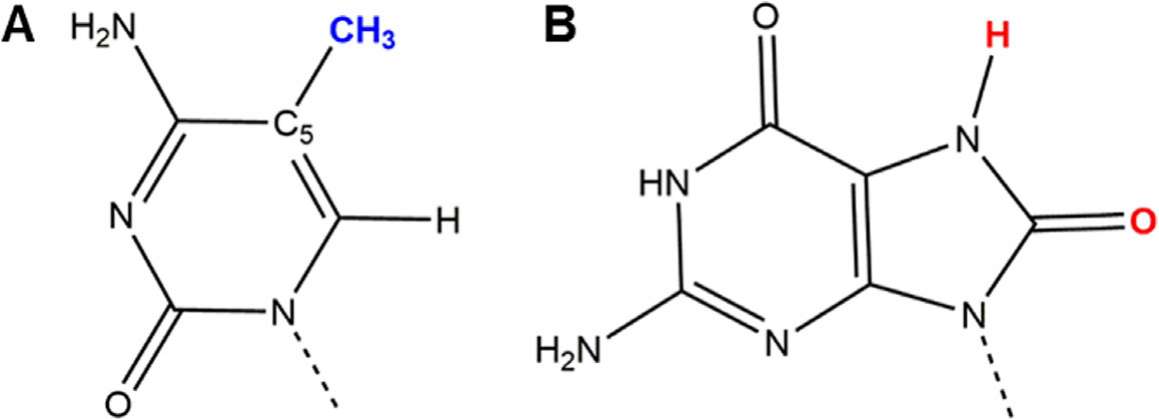
Chemical modifications of cytosine and guanine: (**A**) mC; (**B**) oxoG. The modified groups are color-coded: methyl group at C5 on mC (blue); O8 and H7 on oxoG (red).

Enzymatic methylation of cytosine (C) in the CpG context results in the formation of 5-methylcytosine (mC), a principal epigenetic regulatory base (Figure 1A) (1). In parallel, environmental and endogenous DNA damage results in a number of DNA base lesions. Guanine has the lowest redox potential of the four DNA bases and is the most susceptible to oxidation (2). As a result, one of the most abundant DNA base lesions is represented by 8-oxoguanine (oxoG) (Figure 1B) (3). In addition to being a pre-mutagenic lesion (4), oxoG interferes with the action of many DNA-binding proteins (5–14).

Assuming a consensus level of oxoG as 1 per 10^6^ G (15,16) and 2.8 × 10^7^ CpG dinucleotides per haploid human genome (17), oxoG would be expected to be found in ∼100 CpG dinucleotides per diploid human cell. This number has the potential to rise significantly with an increasing guanine oxidation typical of oxidative stress (18,19). Considering 70–80% of CpG dinucleotides are methylated in the human genome (17,20), possible interference of oxoG with the epigenetic functions of mC may be of biological significance.

Individually, mC and oxoG cause context-dependent perturbations in the stability (21–24), structure (25–28) and dynamics (21,24,29,30) in duplex CpG DNA. We hypothesize that introduction of oxoG to a hemimethylated or fully methylated CpG context can significantly affect the structure and dynamics of this site. The consequences may include alterations in DNA stability, base position within the helix, conformation of the phosphate backbone, shape/charge/H-bonds surface patterns as well as local base and backbone dynamics. These changes may critically affect specific recognition of methylated CpG dinucleotides by proteins involved in epigenetic regulation.

It has been reported that oxoG in CpG sites interferes with the activity of DNMT3a, a human methyltransferase that methylates cytosine at C5 (8). Active demethylation in higher eukaryotes employs two mechanisms: C^5^ oxidation by TET1–TET3 followed by deamination by AID or APOBEC3 family enzymes in mammals, and removal by specific DNA glycosylases (DME, ROS1) in plants. All these enzymes could in principle be affected by the appearance of oxoG in their recognition sites, but this possibility has never been investigated.

To address the consequences of guanine oxidation in methylated GpG dinucleotides, we designed a series of DNA duplexes representing a single oxoG clustered with all possible combinations of mC (*cis*-hemimethylated, *trans*-hemimethylated and fully methylated) within a target CpG site in two different sequence contexts (Table 1). The duplexes were comprehensively analyzed to compare the target effects in the oxidized/methylated duplexes with those in only oxidized and only methylated counterparts. Using UV melting, solution NMR, and molecular dynamics, we have determined the thermodynamic stability of these duplexes, their average structures as well as the rates/amplitudes and correlations of the internal motions. Finally, we show that oxidation of guanine in a methylated CpG dinucleotide significantly affects its recognition by the plant epigenetic demethylase, ROS1.

**Table 1.** Sequences of self-complementary oligonucleotides used in this study

Duplex names	Modifications	PDB IDs	Duplex sequences
C^3^G^4^//C^9^G^10^	None	1BNA ^a^	5′-CG**CG**AATT**CG**CG-3′
			3′-GC**GC**TTAA**GC**GC-5′
C^3^X^4^//C^9^G^10^	oxoG	5IV1 ^b^	5′-CG**CX**AATT**CG**CG-3′
			3′-GC**GC**TTAA**XC**GC-5′
C^3^G^4^//C^9^X^10^	oxoG	5IZP ^b^	5′-CG**CG**AATT**CX**CG-3′
			3′-GC**XC**TTAA**GC**GC-5′
M^3^G^4^//C^9^G^10^	mC	5L06	5′-CG**MG**AATT**CG**CG-3′
			3′-GC**GC**TTAA**GM**GC-5′
C^3^G^4^//M^9^G^10^	mC	5L2G	5′-CG**CG**AATT**MG**CG-3′
			3′-GC**GM**TTAA**GC**GC-5′
M^3^G^4^//M^9^G^10^	mC/mC	6ALT	5′-CG**MG**AATT**MG**CG-3′
			3′-GC**GM**TTAA**GM**GC-5′
M^3^G^4^//C^9^X^10^	mC/oxoG (*trans*)^c^	5UZ1	5′-CG**MG**AATT**CX**CG-3′
			3′-GC**XC**TTAA**GM**GC-5′
C^3^X^4^//M^9^G^10^	mC/oxoG (*trans*)^c^	5TRN	5′-CG**CX**AATT**MG**CG-3′
			3′-GC**GM**TTAA**XC**GC-5′
M^3^X^4^//C^9^G^10^	mC/oxoG (*cis*)^c^	6ALU	5′-CG**MX**AATT**CG**CG-3′
			3′-GC**GC**TTAA**XM**GC-5′
C^3^G^4^//M^9^X^10^	mC/oxoG (*cis*)^c^	5UZ3	5′-CG**CG**AATT**MX**CG-3′
			3′-GC**XM**TTAA**GC**GC-5′
M^3^X^4^//M^9^G^10^	mC/oxoG (full)^c^	6ALS	5′-CG**MX**AATT**MG**CG-3′
			3′-GC**GM**TTAA**XM**GC-5′
M^3^G^4^//M^9^X^10^	mC/oxoG (full)^c^	5UZ2	5′-CG**MG**AATT**MX**CG-3′
			3′-GC**XM**TTAA**GM**GC-5′

**M** = 5-methylcytosine, **X** = 8-oxoguanine.

^a^C^3^G^4^//C^9^G^10^:Drew–Dickerson dodecamer (DDD), self-complementary scaffold 5′-C_1_G_2_**C_3_G_4_**A_5_A_6_T_7_T_8_**C_9_G_10_**C_11_G_12_-3′ used for all the duplexes with the unmodified target CpG site highlighted in bold (79).

^b^published in (5).

^c^ *trans*: mC:oxoG base pair; *cis*: mC-p-oxoG; *full*: oxidized, fully-methylated CpG site.

## MATERIALS AND METHODS

### Oligonucleotides synthesis and purification

Sequences of the oligonucleotides used for the thermodynamics, solution NMR structure and dynamics in this study are listed in Table 1. The details of their synthesis and purification are described in Supplementary Experimental Section and Methods.

### UV melting experiments

Thermodynamic properties of the duplexes was studied by UV melting technique. The experiments were performed using Cary 300 Bio spectrophotometer equipped with a six-cell Peltier thermostated cuvette holder (Agilent Technologies). Melting curves were recorded at wavelengths 260, 270 and 300 nm in the 5–95°C range with temperature change rate 0.5°C/min. The absorbance at 300 nm was subtracted from the values at other wavelengths at each temperature. The optical data were collected every 0.2°C. Thermodynamic parameters of the duplex formation (changes of enthalpy Δ*H*°, entropy Δ*S*° and Gibbs free energy at 37°C, Δ*G*°_37_) were obtained by direct non-linear fitting and concentration methods (31). Fitting of the melting curves were performed using a two-state model corrected for intramolecular hairpin formation as described (32). Thermodynamic parameters obtained at 260 and 270 nm from heating and cooling transitions were averaged. The melting temperature (*T*_m_) was determined as the temperature at which half of the total oligonucleotide is single-stranded. The concentration method is based on the linear dependence of reciprocal temperature on the logarithm of the total oligonucleotide concentration (32,33).

### NMR experiments

NMR data were recorded on Bruker AVANCE III spectrometers operating at 500, 600, 700 and 850 MHz (^1^H Larmor frequencies). All the duplexes were exchanged to 10 mM sodium phosphate (pH 6.8), 50 mM NaCl, 1 mM EDTA and 0.3-1.1 mM oligonucleotide dissolved in either H_2_O + D_2_O at a 9:1 ratio or 100% D_2_O. The standard 1D ^1^H, 2D ^1^H–^1^H and 2D ^1^H–^31^P NMR data were recorded (details are in Supplementary Experimental Section and Methods).

### Restrained and free molecular dynamics

All molecular dynamics simulations were conducted with AMBER 14 (34). Distance and dihedral restraints enforced during the simulated annealing protocol were derived from the 2D ^1^H–^1^H NOESY resonance line intensities and ^31^P chemical shift values, respectively. Free molecular dynamics simulations were run for 600 ns using the PMEMD.cuda implementation of Sander with ffOL15 force field (35–37). Other details of the simulations and analysis are described in Supplementary Experimental Section and Methods.

### Structure visualization and analysis

The structures were visualized and analyzed with VMD (38), 3DNA (39) and Chimera (40). The molecular dynamics trajectories were analyzed using CPPTRAJ (41) and in-house scripts.

### Enzyme kinetics

Oligonucleotides containing a single CpG site or its mC- and/or oxoG-containing derivatives (Supplementary Table S1) were 5′-^32^P-labeled and annealed to form the required double-stranded substrate. Cloning and purification of the catalytic fragment of tobacco ROS1 (NtROS1cat) is described in the Supplementary Experimental Methods. The reaction mixture (60 μl) contained 50 nM substrate, 430 nM NtROS1cat, 50 mM Tris–HCl (pH 8.0), 100 mM NaCl, 1 mM dithiothreitol, 1 mM EDTA and 0.1% bovine serum albumin. The reaction mixture was incubated at 37°C, and 5-μl aliquots were withdrawn at 2–240 min, mixed with an equal volume of formamide dye (80% formamide, 20 mM EDTA, 0.1% xylene cyanol, 0.1% bromophenol blue) and heated for 5 min at 95°C. The reaction products were resolved by electrophoresis in 20% polyacrylamide/8 M urea gel, and the apparent rate constant *k*_cat_ was derived by fitting the data to the equation

\begin{equation*}\ \left[ {\rm{P}} \right] = a{\left[ {\rm{S}} \right]_0}\ \left( {1 - {e^{ - {k_{{\rm{cat}}}}t}}} \right)\end{equation*}

where [P] is the product concentration, [S]_0_ is the initial substrate concentration, *a* is binding bias coefficient and *t* is the reaction time.

## RESULTS

### Sample design

To put the methylated/oxidized CpG dinucleotides into a known structural and dynamic context, we have designed a series of substrates based on the Drew–Dickerson sequence (DDD, Table 1), which had been characterized by solution NMR in the unmodified and oxidized state (42). As DDD has two CpG sites, we had two series of the target samples: one clustering oxidation at G4 with *trans*-hemimethylation (C^3^X^4^//M^9^G^10^), *cis*-hemimethylation (M^3^X^4^//C^9^G^10^) and full methylation (M^3^X^4^//M^9^G^10^) in the sequence context 5′…GCGA…3′, another clustering oxidation at G10 with *trans*-hemimethylation (M^3^G^4^//C^9^X^10^), *cis*-hemimethylation (C^3^G^4^//M^9^X^10^) and full methylation (M^3^G^4^//M^9^X^10^) in the sequence context 5′…TCGC…3′. Control samples included oxidized-only (C^3^X^4^//C^9^G^10^, C^3^G^4^//C^9^X^10^) and methylated-only (M^3^G^4^//C^9^G^10^, C^3^G^4^//M^9^G^10^, M^3^G^4^//M^9^G^10^) DNA (Table 1). Double oxidation is not expected to occur with any biologically relevant frequency and therefore was not investigated.

### Thermodynamic stability of the DNA duplexes

Analysis of the thermal denaturation profiles at different wavelength showed a good agreement between theoretical and experimental curves (Supplementary Figures S1 and S2). The heating and cooling curves were close to each other. The thermodynamic parameters obtained from heating and cooling at 260 and 270 nm did not differ by more than 15% and thus were averaged. The values of Δ*H*° and Δ*S*° obtained at different concentrations also did not differ by more than 15% except in the M^3^G^4^//M^9^G^10^ duplex. In all cases, the values of Δ*G*° were different by less than 10% due to the enthalpy-entropy compensation (43). The Δ*H*° values increased as the concentration of oligonucleotides decreased because of the presence of hairpin structure at concentrations below 10^−5^ M (32). *T*_m_ values were calculated using the average thermodynamic parameters for the standardized total oligonucleotide concentration 2.5 × 10^−5^ M. The linear dependences of 1/*T*_m_ on the logarithm of concentration with *r*^2^ > 0.95 were observed for all duplexes (Supplementary Figure S3), indicating that the population of hairpins, which may form due to self-complementarity of the oligonucleotides, is minimal, and the two-state model of duplex formation is adequate. The thermodynamic parameters obtained by non-linear fitting and the concentrations method are shown in Supplementary Table S2. Although the enthalpy and entropy changes obtained by these methods deviated by >20% for several sequences, the difference in Gibbs free energies was less than 15% for all duplexes. The typical difference in melting temperatures was less than 1.5°C, with only two exceptions of fully methylated oxidized duplex (Supplementary Table S2).

The effects of base modifications on the thermal stability of the duplexes are summarized in Table 2. With the exception of the fully methylated oxidized CpG sites, methylation of a single C in the 3^rd^ position slightly stabilized the duplexes by 0.3–1.7°C, consistent with literature reports on the better duplex-forming properties of mC (44,45), whereas methylation of C in the 9^th^ position slightly destabilized the duplexes by 1.7–2.9°C. Introduction of two mC residues into the unmodified duplex caused no change in thermal stability. The introduction of oxoG to any context dramatically destabilized the duplexes by 10–20°C and increased Δ*G*° by up to 4 kcal/mol. Such impact was reported for unmodified oligonucleotides (23), and here we have found that oxoG obviously overcomes the stabilizing effect of mC when one is observed.

**Table 2.** Melting temperature, Gibbs free energy and their non-additivity for modifications in the Drew–Dickerson sequence

	Experimental^b^		Difference with unmodified duplex^c^				Modification non-additivity	
Duplex^a^	*T*_m_	Δ*G*°	Δ*T*_m exp_	ΔΔ*G*°_exp_	Δ*T*_m calc_	ΔΔ*G*°_calc_	Δ*T*_m calc_−Δ*T*_m exp_	ΔΔ*G*°_calc_−ΔΔ*G*°_exp_
C^3^G^4^//C^9^G^10^	66.6	−12.6						
C^3^X^4^//C^9^G^10^	49.4	−8.8	−17.1	3.9				
C^3^G^4^//C^9^X^10^	51.2	−8.7	−15.3	3.9				
M^3^G^4^//C^9^G^10^	68.0	−12.2	1.4	0.4				
C^3^G^4^//M^9^G^10^	64.6	−11.2	−1.9	1.4				
M^3^G^4^//M^9^G^10^	66.3	−11.1	−0.2	1.5	−0.5	1.8	−0.3	0.3
M^3^G^4^//C^9^X^10^	51.5	−9.2	−15.1	3.4	−13.9	4.3	1.2	0.9
C^3^X^4^//M^9^G^10^	46.6	−7.7	−20.0	5.0	−19.1	5.2	0.9	0.2
M^3^X^4^//C^9^G^10^	50.4	−8.0	−16.2	4.5	−15.7	4.2	0.4	−0.2
C^3^G^4^//M^9^X^10^	48.3	−8.1	−18.3	4.5	−17.3	5.3	1.0	0.8
M^3^X^4^//M^9^G^10^	56.9	−9.6	−9.7	3.1	−17.7	5.6	−7.9	2.5
M^3^G^4^//M^9^X^10^	55.9	−9.5	−10.7	3.1	−15.9	5.7	−5.2	2.6

^a^M, 5-methylcytosine; X, 8-oxoguanine.

^b^All temperatures are in°C, all Gibbs free energies are in kcal/mol.

^c^exp, experimentally measured; calc, calculated as the sum of experimentally measured effects of individual modifications.

The destabilizing effect of oxoG in non- and hemimethylated contexts was also moderately position-dependent in the Drew–Dickerson sequence: the decrease in *T*_m_ caused by oxoG in position 4 (−17.2°C to −18.0°C) was on average ∼1.5°C more pronounced than when position 10 was oxidized (−15.4°C to −16.5°C). Since position 10 is closer to the end of the symmetric duplex than position 4, this difference is more likely due to the nature of the lesion rather than to the terminus proximity effect, which would destabilize position 10 to a higher degree.

While the non- and hemi- methylated CpG dinucleotides behaved nearly additively in terms of the effect of the modifications on *T*_m_ and Δ*G*°, the fully methylated sequences showed quite a different picture. In the fully methylated duplex, oxoG had a much less pronounced destabilizing effect, decreasing *T*_m_ only by ∼10°C in both positions, with the difference in Δ*G*° ∼2.5 kcal/mol less than expected from an additive model (Table 2). The observed non-additive effects of mC and oxoG likely reflect structural or dynamic interactions of the modified bases within the CpG dinucleotide.

### NMR characterization of the DNA duplexes

**Figure 2.**
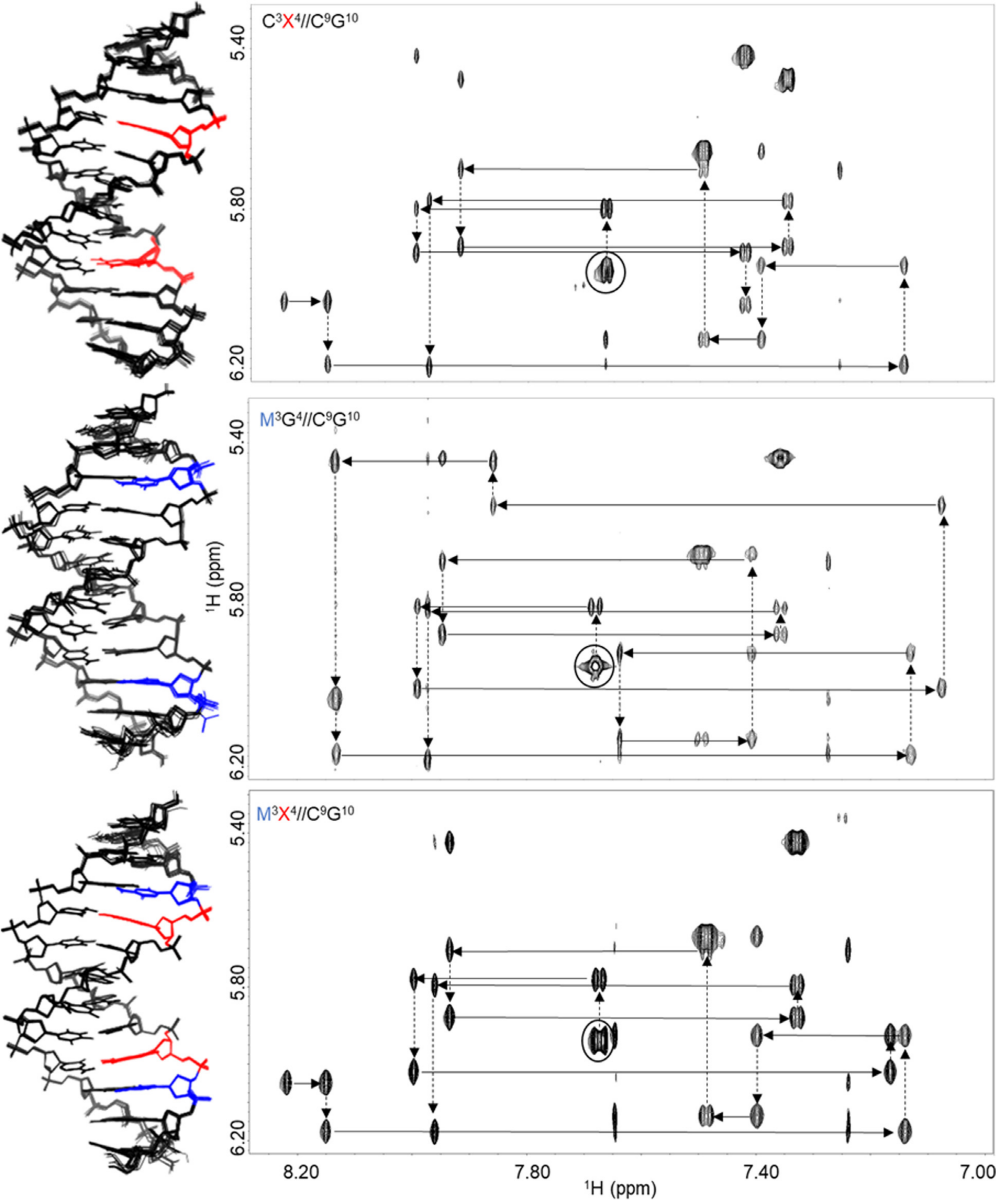
Solution NMR structures and ^1^H–^1^H NOESY ‘walk regions’ of representative duplexes: C^3^X^4^//C^9^G^10^ (oxidized only), M^3^G^4^//C^9^G^10^ (methylated–only) and M^3^X^4^//C^9^G^10^ (oxidized and methylated) in 99.9% D2O at 298 K (*T*_mix_ 200 ms). Solid arrows indicate inter-base—sugar NOEs. Dashed arrows show intra-sugar—base NOEs. Beginning with the cytosine-1 H5-H6 NOE (black circle), H8/H6 base protons to H1′ sugar NOEs were identified in the sequential manner. Sequential base-H1′ connectivities involving oxoG were disrupted due to the loss of the H8 proton.

The composition of the 2D ^1^H–^1^H and ^1^H–^31^P resonance lines indicated that each duplex adopts a single A/B-DNA conformation. The 2D NOESY peak patterns for all the duplexes were analogous to the NMR signature of d(CGCGAATTCGCG), the Drew-Dickerson dodecamer, a *de facto* A/B DNA standard (46). The 2D NOESY recorded in 90% H_2_O confirmed Watson-Crick hydrogen bonding for all base pairs (2D NOESY of an imino-base region, Supplementary Figure S4). The 2D NOESY spectra delivered a sufficient density and uniformity of ^1^H–^1^H NOE restraints for every duplex (Supplementary Table S3). All oxoG-containing duplexes presented distinct spectral features: an absence of purine H8 NOESY cross-peaks for oxoG (Figure 2), a downfield shift for H2″ (>2.33 ppm) with respect to DDD, and presence of peaks involving HN7 (Supplementary Figure S5). Duplexes containing mC displayed a NOESY/TOCSY cross-peaks of its H6 with the methyl group at C5 and lacked the characteristic H5–H6 peak in the ‘walk region’ (Supplementary Figure S6).

**Figure 3.**
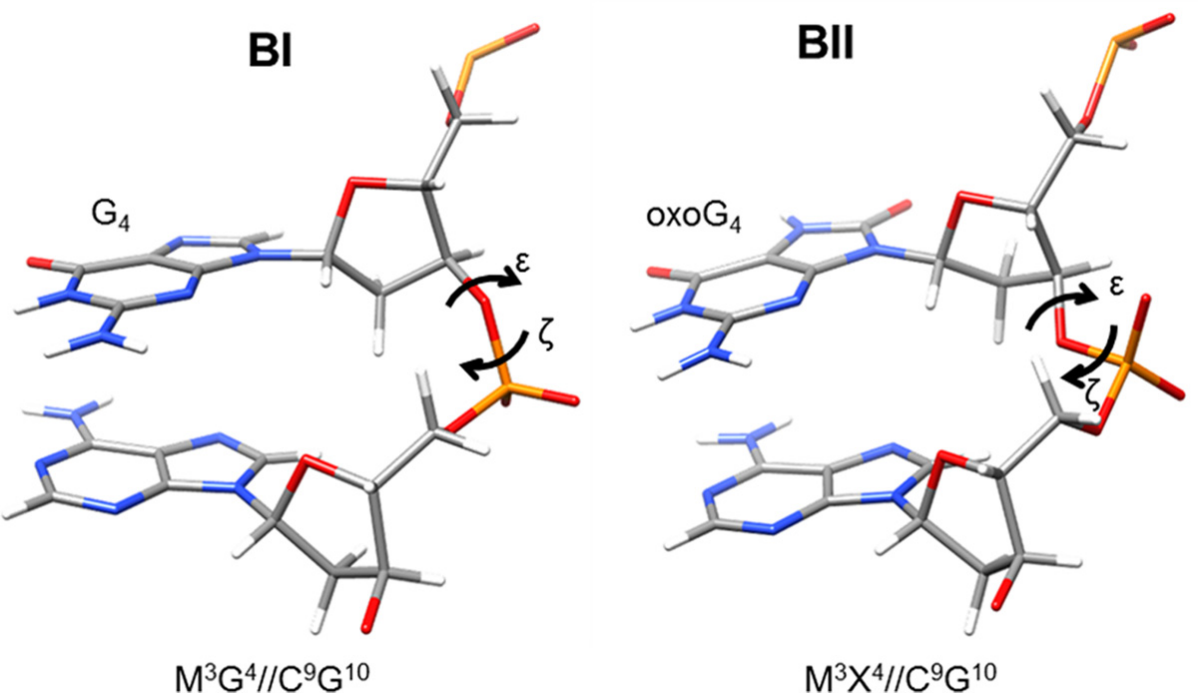
Representative BI and BII conformations for phosphate 3′ of oxoG. The backbone adopts a different conformation at the ϵ and ζ dihedrals for the BI and BII states.

The conformational flexibility and dynamics of the DNA backbone are vital for protein-DNA recognition (47). One such determinant is the correlated transitions between the ϵ and ζ DNA backbone torsion angles defining two conformational states (Figure 3), BI (ϵ – ζ < 0, more common) and BII (ϵ – ζ > 0, less common) (48). ^31^P NMR chemical shifts values can provide valuable insight regarding the relative populations of BI and BII states in duplex DNA (49). Both 1D and 2D ^1^H-^31^P NMR spectra for duplexes in this study presented the majority of ^31^P resonances within ∼0.5–0.8 ppm (Supplementary Table S4) indicating a strong prevalence of BI states, as previously reported for the unmodified sequence (50). The ^31^P lines from oxoG phosphate groups were shifted downfield by ∼0.15–0.44 ppm from the cluster of other ^31^P lines in every oxoG-containing duplex (Supplementary Figure S7, Supplementary Table S4). The chemical shift values of oxoG ^31^P resonances indicate a transition in the phosphate backbone from BI to BII range (49).

### Solution NMR structures

The NMR solution structure was determined for all modified duplexes listed in Table 1. For each duplex, an ensemble of lowest-energy structures was derived from restrained MD simulations (Figure 2, Supplementary Figure S8). Statistics pertaining to the quality of the final models are presented in Table 3. The greatest NOE restraint violation for any model was 0.35 Å and dihedral violations did not exceed 17.6°. Within each ensemble, the largest pairwise backbone RMSD value was no >1.06 Å. The structural differences between duplexes were minor (backbone RMSD of 0.62–2.34 Å) and no major structural alterations outside the target CpG sites were detected. In addition, our structures were similar to known X-ray and solution NMR DDD structures (PDB 1BNA, 355D and 1NAJ; backbone RMSD of 1.26–3.95 Å).

**Table 3.** NOE distance restraints derived from 2D ^1^H NOESY recorded in D_2_O

Duplex	models per ensemble	Greatest backbone RMSD within ensemble (Å)	Backbone RMSD range against representative models (Å)	Greatest NOE violation (Å)	Greatest dihedral angle violation (degrees)
C^3^X^4^//C^9^G^10^	13	0.66	1.20-1.86	0.07	7.49
C^3^G^4^//C^9^X^10^	14	0.87	1.13-2.05	0.10	10.2
M^3^G^4^//C^9^G^10^	8	0.89	0.62-2.22	0.03	17.6
C^3^G^4^//M^9^G^10^	8	0.69	0.62-2.34	0.17	13.5
M^3^G^4^//M^9^G^10^	10	0.71	0.89-2.10	0.04	13.2
M^3^G^4^//C^9^X^10^	11	0.80	1.07-2.23	0.08	15.1
C^3^X^4^//M^9^G^10^	11	0.85	1.52-2.35	0.14	12.5
M^3^X^4^//C^9^G^10^	10	0.80	1.08-2.26	0.19	11.3
C^3^G^4^//M^9^X^10^	9	1.06	0.88-2.36	0.07	13.7
M^3^X^4^//M^9^G^10^	10	0.68	1.17-1.96	0.35	8.9
M^3^G^4^//M^9^X^10^	13	0.84	0.90-1.82	0.09	15.9

All oxidized duplexes had ϵ and ζ torsion angles of the oxoG nucleotide in the BII range. Minor unwinding (lowering of the twist parameter values) was observed for the fully-methylated/oxidized duplexes, M^3^X^4^//M^9^G^10^ and M^3^G^4^//M^9^X^10^ with respect to the majority of the methylated-only counterparts (Supplementary Table S5), whereas in M^3^X^4^//C^9^G^10^ twist was elevated by several degrees above all the rest. These findings are corroborated by the free MD analysis (below).

### Imino proton NMR line widths and base dynamics

**Figure 4.**
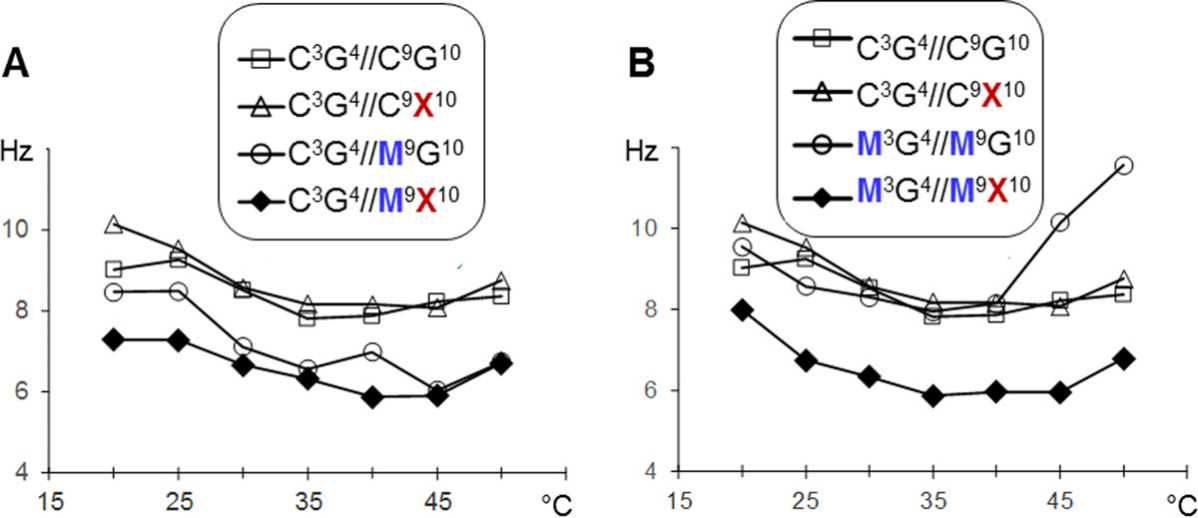
1D ^1^H NMR linewidths for G4 in selected sets of samples as function of temperature. The error bars on both graphs, 0.3 Hz, are of the same size as the data characters. For this reason, the error bars are not shown.

The NMR linewidths of the base imino protons provide an informative probe into base pair opening dynamics: broader lines indicate increased base pair opening rates and reduced base pair stability. The imino proton resonance assignment for each oligonucleotide was completed using imino-base and imino-imino cross peaks from the ^1^H–^1^H NOESY in 90% H_2_O (Supplementary Figure S4). Guanine and oxoG base imino proton NMR line widths were monitored as a function of temperature (5–60°C, in 5°C increments). Oxidation of guanine led to imino proton line broadening for the guanine base in all the cases, a common trend reported for oxoG containing DNA (21,24). Methylation (mC), on the other hand, predominantly resulted in imino proton line narrowing for adjacent guanine bases, in accordance with the general view that mC stabilizes duplex DNA (22). For both *cis-* and *trans-*methylation, the typical outcome followed an expected trend, where mC/oxoG clustering narrowed imino proton lines with respect to oxidized only CpG guanines and broadened imino proton lines with respect to methylated only CpG guanines (Figure 4, Supplementary Figure S9). The most notable exception from this trend is narrowing of imino ^1^H for G4 within C^3^G^4^//M^9^X^10^ with respect to C^3^G^4^//M^9^G^10^ at temperature values below 45°C (Figure 4A). Surprisingly, mC/oxoG clustering (full methylation + oxidation) in one sequence context resulted in imino ^1^H line width narrowing for non-oxidized guanines (G4) within the M^3^G^4^//M^9^X^10^ CpG sites in comparison to those for the same base within C^3^G^4^//C^9^X^10^ and M^3^G^4^//M^9^G^10^ CpG sites (Figure 4B). Notably, both effects observed (Figure 4) describe the stabilization of the base pair involving non-oxidized G4 base in response to oxidizing the guanine in the opposing strand of the CpG site (G10 → X10) for both *trans*-hemimethylation and full methylation. These effects were only observed in one sequence context and thus are considered to be sequence dependent (Supplementary Figure S9).

### Molecular dynamics reproduces the solution NMR structures

The backbone root mean square deviations (RMSD) of each 600 ns simulation was monitored as a function of simulation time to assess stability and divergence from the initial starting structure. The backbone RMSD from the initial starting structure for all twelve simulations was stable, fluctuating between 1.0 and 2.5 Å when monitoring the 8 internal base pairs of the duplex (Supplementary Table S6 and Supplementary Figure S10). The average values and standard deviations for helical and backbone parameters were within two standard deviation values of the solution NMR structures over the last 555 ns of MD analyzed (Supplementary Table S7; Supplementary Figures S11 and S12). The exceptions to this observation are extremely rare (less than 3% of all the occurrences in our 11 duplexes and 19 parameters monitored). All phosphate backbone steps adopting BII conformation in the NMR structures were reproduced in our free MD simulations.

### BI/BII backbone conformations in free MD

The free MD simulations revealed that clustering oxoG and mC affected the preference for BII backbone phosphate conformation at the G4 and G10 positions (Supplementary Figure S13, Table 4). Specifically, duplexes containing oxoG in a fully*-*methylated CpG had the greatest fraction of BII phosphate conformation of the oxoG backbone (89% BII for M^3^X^4^//M^9^G^10^ and 86% BII for M^3^G^4^//M^9^X^10^). Notably, these percentages of BII conformation were significantly higher than those in only oxidized (79% for C^3^X^4^//C^9^G^10^ and 69% for C^3^G^4^//C^9^X^10^) and only fully-methylated duplexes at the same positions (60–72%). *Trans*-methylation of oxidized duplexes leads to a significant elevation of the BII conformation for the down strand non-oxidized guanines (by 29% for M^3^G^4^//C^9^X^10^ and 35% for C^3^X^4^//M^9^G^10^) and minor increase for oxoG (by 8–10%) against the oxidized-only counterparts. In comparison with methylated-only duplexes, oxidized/trans-methylated CpG sites showed a considerable increase in BII conformation for the oxoG position (41% for C^3^X^4^//M^9^G^10^ and 54% for M^3^G^4^//C^9^X^10^). Clustering of oxoG with a *cis*-mC resulted in minor increases in BII conformation for oxoG (by 5–7%) with respect to the methylated only duplexes while showing modest decrease (by 8%) or no change in BII with respect to oxidized-only analogs. This decrease in BII conformation upon methylation can be attributed to reshaping of the free energy profiles as reported previously (51). Some dinucleotide steps other than oxoG (e.g. C9pG10) also showed a higher propensity for BII conformation as pyrimidine-purine base pair steps in B-DNA are intrinsically flexible (52–55).

**Table 4.** BII % per phosphate step for the target CpG cites in MD simulations

	BII % per Phosphate Step					
Duplex	G2pC3	C3pG4	G4pA5	T8pC9	C9pG10	G10pC11
C^3^G^4^//C^9^G^10^	30	34	46	5	47	25
C^3^X^4^//C^9^G^10^	18	3	79	9	53	24
C^3^G^4^//C^9^X^10^	16	31	49	7	9	69
M^3^G^4^//C^9^G^10^	1	7	72	3	54	23
C^3^G^4^//M^9^G^10^	15	38	48	2	12	56
M^3^G^4^//M^9^G^10^	1	8	72	0	7	60
M^3^G^4^//C^9^X^10^	2	5	78	3	3	77
C^3^X^4^//M^9^G^10^	15	1	89	7	10	59
M^3^X^4^//C^9^G^10^	5	0	79	7	51	22
C^3^G^4^//M^9^X^10^	20	34	51	0	2	61
M^3^X^4^//M^9^G^10^	1	0	89	3	10	54
M^3^G^4^//M^9^X^10^	2	9	71	0	1	86

In order to reveal possible causes of the context-independent elevated BII occurrence 3′ to oxoG nucleotides, we have analyzed several key types of non-covalent interactions including stacking between base pairs as well as base-to-sugar electrostatic and steric interactions within oxoG nucleotides. The O8…O4′ distance in all oxoG4 and oxoG10 nucleotides had the value of 3.73 ± 0.05 Å (average ± standard deviation) whereas the corresponding distances in non-oxidized G4 and G10 nucleotides (H8…O4′) were significantly shorter at 3.41 ± 0.08 Å. Our NMR structures report a highly similar pattern: 3.62 ± 0.15 Å for O8…O4′ (in oxoG) and 3.25 ± 0.11 Å for H8…O4′ (in G4 and G10). This wider separation in oxoG was earlier proposed as a means to alleviate the electrostatic repulsion between partially negatively charged O8 and O4′ atoms. (56,57) In addition, we compared the intra-nucleotide base-to-sugar distances, specifically O8…H2′ (in oxoG) and H8…H2′ (in guanines). For each such pair of atoms, we measured the average of the shortest 1% of the distance values from the free MD trajectories. For the O8…H2′ pairs in oxoG this value was 2.25 ± 0.01 Å whereas for the non-oxidized G4 and G10 nucleotides the H8…H2′ value was significantly lower at 1.98 ± 0.01 Å. These distances together with the atomic radii of H (1.29 Å) and O (1.66 Å) suggest that a replacement of H8 with O8 upon guanine oxidation creates a potential for base-to-sugar steric clash involving the O8 and H2′ atoms.

### Effects of oxoG/mC clustering on the internal dynamics of CpG sites

To assess the dominant modes of motion of each modified CpG site, we employed principal component analysis (PCA). In PCA, the dimensionality of the simulation data is reduced by orthogonal transformations to a covariance matrix which is then decomposed to set of orthogonal eigenvectors called principal components (PCs). The PCs correspond to concerted atomic motions, which can be ranked based on the amplitudes of structural variance: dominant movements are described by eigenvectors with the largest eigenvalues (58–61). Motions of similar nature and amplitude will have similar eigenvector projections and eigenvalues.

**Figure 5.**
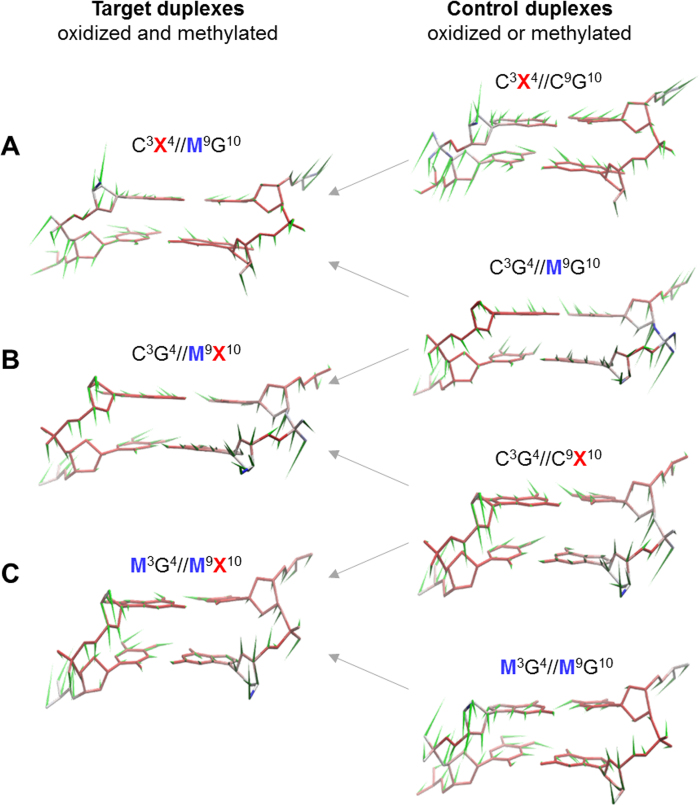
The porcupine plots representing the internal motions for principal component 1 (PC1) of the target CpG site molecular dynamics for selected samples. oxoG in duplex labels (X) is colored in red. mC in duplex labels (M) is colored in blue. Target duplexes described in the text are on the left and labeled A, B, and C; the respective controls—on the right. Arrows indicate the control/target relatedness.

We applied PCA to all heavy atoms of the target C3pG4 site and determined the dominant motional modes (Supplementary Figure S14). In all the duplexes, the first principal component (PC1) dominates the internal motions (26–38% of the structural variance), while the first five modes (PC1–PC5) contribute >69% of the variance. Notably, PC1 projections exhibit minimal overlap between the duplexes, indicating that the most dominant dynamic movements have been altered with the incorporation and clustering of the modifications in distinct sequence contexts (Supplementary Figure S15). In contrast, PC2–5 display greater overlap and projection similarities across the duplexes highlighting similarities in motion (Supplementary Figure S15). Therefore, the rest of our analysis describes the effect of clustered oxidation and methylation on the concerted motions of PC1 (Figure 5, Supplementary Figure S15). The ‘porcupine plot’ approach (61) allowed us to graphically represent and compare the directions and magnitudes of atomic motions. In addition, we built a clustering tree for the PC1 profiles derived from a complete set of pairwise Kolmogorov distances (62) between all the samples (Supplementary Figure S16). The clustering tree and underlying quantification allow us to corroborate the visual inspection and comparison of the porcupine plots. For the oxidized and *trans-*methylated CpGs (C^3^X^4^//M^9^G^10^), the strand containing mC showed somewhat altered directions of motion in the backbone atoms after methylation of the oxidized site (Figure 5A). On the other hand, oxidation of the methylated CpG site leads to alterations in directions and in many cases in amplitudes of both strands of the C^3^X^4^//M^9^G^10^ CpG (Figure 5A). These observations are in agreement with the fact that samples C^3^X^4^//C^9^G^10^, C^3^G^4^//M^9^G^10^ and C^3^X^4^//M^9^G^10^ are not forming a cluster on the PC1 tree as the respective collective motions are substantially dissimilar (Supplementary Figure S16). The *cis*-methylated CpG sites (C^3^G^4^//M^9^X^10^) demonstrate dynamic movements similar to those of the oxidized-only CpG and mildly different directions of atomic motions with respect to methylated-only CpGs (Figure 5B). The PC1 tree reports that C^3^G^4^//C^9^X^10^, C^3^G^4^//M^9^G^10^ and C^3^G^4^//M^9^X^10^ are forming a cluster supporting the similarity of motions observed in the porcupine plots. The fully methylated and oxidized sample M^3^G^4^//M^9^X^10^ demonstrates high similarity of PC1 motions with those of its fully methylated control within most of the structure: the backbone and the non-oxidized base pair (Figure 5C). Only motions within the oxidized base pair and to a lesser degree within a part of the backbone are similar between M^3^G^4^//M^9^X^10^ and its oxidized control. These observations of the porcupine plots in Figure 5C are in general agreement with PC1s of M^3^G^4^//M^9^X^10^ and M^3^G^4^//M^9^G^10^ being loosely clustered (Supplementary Figure S16). Alternative sequence contexts can either preserve the observed differences in motions (e.g. M^3^G^4^//C^9^X^10^) or result in a reduction of motional differences between the oxidized/methylated versus oxidized-only and methylated-only CpGs (M^3^X^4^//C^9^G^10^ and M^3^X^4^//M^9^G^10^) (Supplementary Figure S17).

Certain types of mC/oxoG clustering display strong effects on the ranges of helical parameters shift and tilt presented by the PC1 pseudo-trajectories (Supplementary Table S8). For instance, the variation of shift for the oxidized/*trans*-methylated M^3^G^4^//C^9^X^10^ CpG (0.56 Å) is much smaller than the corresponding variations for the only oxidized C^3^G^4^//C^9^X^10^ (2.31 Å) and only methylated M^3^G^4^//C^9^G^10^ (3.28 Å) CpGs. A similar reduction in shift variability for PC1 was observed for C^3^X^4^//M^9^G^10^ against C^3^G^4^//M^9^G^10^ and C^3^X^4^//C^9^G^10^. Likewise, full methylation of the C^3^G^4^//C^9^X^10^-containing CpG site led to a noticeable reduction in the variation of shift (C^3^G^4^//C^9^X^10^ vs. M^3^G^4^//M^9^X^10^). This effect is sequence-specific as the full methylation of the C^3^X^4^//C^9^G^10^ CpG does not alter the range of shift. The helical parameter tilt shows dramatic variation reduction upon clustering of oxoG and *trans*- or fully-methylated mC with respect to only-oxidized and only-methylated analogs.

### Effect of oxoG on mC-removing activity of ROS1 DNA glycosylase

To see whether the effects of oxoG on the CpG dinucleotide dynamics may be of biological significance, we addressed the ability of ROS1 DNA glycosylase to remove mC from oxoG-modified CpG dinucleotides. ROS1 is a plant enzyme, biochemically characterized from *Arabidopsis* (63) and tobacco (64), which promotes active demethylation of mCpG and mCpNpG sites. We have cloned and purified the catalytically active fragment of tobacco ROS1 (NtROS1cat) and tested its activity in a full set of possible combinations of one or two mCs and none or one oxoG in the CpG context. The substrates were not Drew–Dickerson duplexes, which are too short for the enzyme to recognize efficiently, but 32-mer duplexes containing a target CpG dinucleotide in the middle. Since ROS1 is a slowly acting enzyme (63), Michaelis–Menten kinetics cannot be used to characterize it, and we have used pseudo-single-turnover conditions ([E]_0_ >> [S]_0_) to follow the rate of cleavage of the radioactively labeled substrate. It should be kept in mind that when only one strand is labeled, the cleavage extent depends not only on the cleavage rate but also on the initial orientation of the bound enzyme recognizing the CpG dinucleotide: the apparent cleavage rate and maximal product concentration will decrease if the oligonucleotide is bound in the orientation with the unlabeled strand probed and cleaved by the enzyme. To account for this, we have applied the binding bias coefficient ranging from 0 (enzyme binds only to unlabeled strand and never moves to the labeled strand) to 1 (enzyme binds to the labeled strand or eventually moves to it over the reaction time followed).

**Figure 6.**
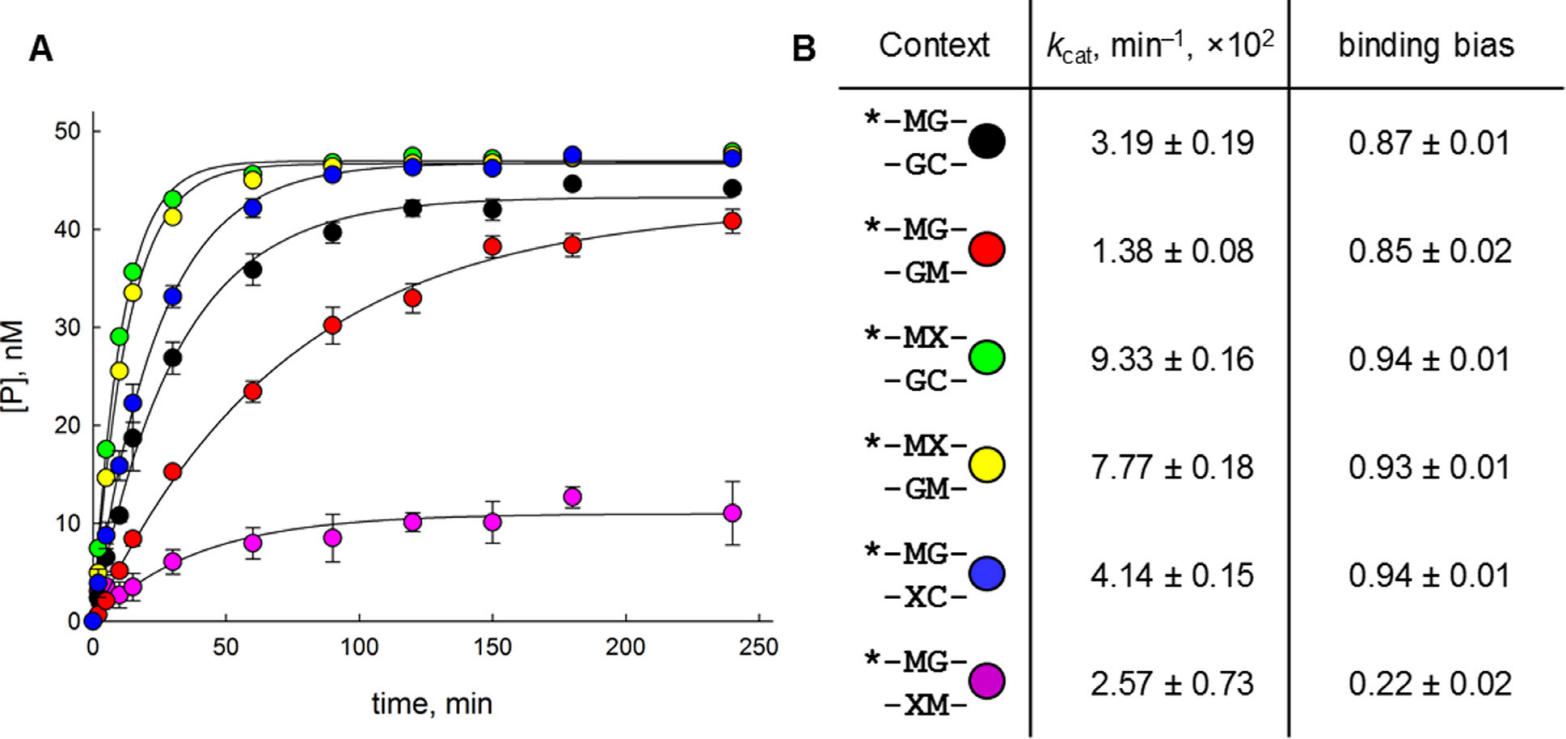
(**A**) time courses of product accumulation during the excision of mC from modified CpG contexts by NtROS1cat. Black, *MG//CG; red, *MG//MG; green, *MX//CG; yellow, *MX//MG; blue, *MG//CX; magenta, *MG//MX. Mean ± S.E.M. of three independent experiments is shown (in some cases, the error bars are fully covered by the symbols). (**B**) Kinetic parameters derived by fitting the data in Panel A.

In all cases except for the *MG//MX substrate, the binding bias coefficient was close to 1, indicating that the enzyme was able to excise mC from the labeled strand almost completely independently of the initial binding orientation (Figure 6). The orientation effect here may be partially reflected in the apparent rate constant (*k*_cat_) values: for example, the *MG//MG substrate is processed about twice as slow as *MG//CG, possibly because about half of the first substrate is bound with the non-labeled strand in the active site of the enzyme, in which case the labeled strand can be cleaved only after the release of the duplex and its re-binding in the opposite orientation. The introduction of oxoG next to mC in hemi- or fully methylated DNA increased the apparent cleavage rate constant 3–5.5-fold and only slightly increased the binding bias (compare *MG//CG with *MX//CG or *MG//MG with *MX//MG). In stark contrast, the introduction of oxoG opposite to mC had a moderate effect on *k*_cat_ (1.3–1.9-fold increase) but totally different consequences for binding bias depending on the methylation status. Whereas in the hemimethylated dinucleotide (*MG//CX) almost all substrate was processed, the labeled strand in the fully methylated substrate (*MG//MX) was significantly more resistant to cleavage than in the absence of oxoG (Figure 6). Comparing the data for *MG//MG, *MX//MG and *MG//MX, one can suggest that ROS1 indeed preferentially binds the oxoG-containing strand in the fully methylated oxidized CpG dinucleotide.

## DISCUSSION

This work centers around the question of how clustering of two chemical modifications of DNA bases—enzymatic cytosine methylation (mC) occurring as part of epigenetic regulation and guanine oxidation (oxoG) viewed primarily as a type of DNA damage—affects the biophysical properties of duplex DNA. As frequency estimates suggest that there may be hundreds of oxidized methylated CpG sites in the human cell (65), this question is biologically relevant. Moreover, it has been reported that such clustering influences the enzymatic activity of DNA repair and genome defense systems, including 8-oxoguanine DNA glycosylase OGG1, AP endonuclease APEX1, and enzymes involved in CpG epigenetic methylation and demethylation (8,65,66). In this work, we have combined solution NMR, molecular dynamics, and UV melting techniques to follow the structural and dynamic consequences of guanine oxidation in CpG dinucleotides with various degree of methylation. Further, we addressed the biochemical effect of such damage by studying the kinetics of ROS1, a DNA glycosylase that removes mC from DNA.

Individually, both mC and oxoG are known to affect duplex stability and dynamics (5,21–30). The position of 5-methyl and 8-oxo chemical moieties in close proximities within the major groove, together with the steric and ion-dipole interactions between 8-oxo and the sugar–phosphate moiety of the oxoG nucleotide, might be expected to disturb the conformational dynamics of methylated and oxidized CpG sites compared with either unmodified or individually modified DNA. Indeed, we observe, both experimentally and computationally, that oxidation of some methylated duplexes leads to noticeable alterations in DNA motion with respect to the methylated-only controls. On the other hand, methylation of cytosine within oxidized CpG sites also alters DNA dynamics, albeit to a lesser degree than the effects of oxoG on methylated DNA. On a macroscopic level, these changes translate into thermodynamic effects in which duplex destabilization by oxoG depends, sometimes non-additively, on the presence of mC in the CpG site.

In general, oxoG, in agreement with the literature data, destabilized dsDNA, with a moderate positional effect in the degree of destabilization likely caused by the nucleotide context. Unexpectedly, the destabilizing effect of oxoG was significantly less pronounced in the fully methylated context (M^3^G^4^//M^9^X^10^ and M^3^X^4^//M^9^G^10^). Also, in this context, the effect of modifications on the duplex stability was non-additive, implying structural interactions between the modified bases. Structurally, oxidation of guanine within CpG resulted in the preference for BII phosphate conformation 3′ to oxoG in all methylation contexts. The dynamics of CpG bases were affected in generally expected ways: oxidation of G intensified motions while methylation of C suppressed the dynamics. Exceptions to these effects were oxidation in *cis*-hemimethylated (C^3^G^4^//M^9^X^10^) and fully methylated CpG (M^3^G^4^//M^9^X^10^) resulting in lowered base-pair opening rates for non-oxidized G4 in comparison with methylated only duplexes (C^3^G^4^//M^9^G^10^ and M^3^G^4^//M^9^G^10^, respectively). Analysis of dominant motional modes from molecular dynamics simulations showed that the amplitudes and directions of atomic motions are altered to the highest extent in the oxidized and *trans*-hemimethylated (C^3^X^4^//M^9^G^10^) and fully-methylated (M^3^G^4^//M^9^X^10^) duplexes. Since the dominant motional modes describe collective motions where contributions of individual atoms are combined and impossible to isolate, we also analyzed variations of the helical parameters specific to those motions to obtain a more conventional description of the effects. Analysis of PC1 pseudo-trajectories yielded that variations of certain helical parameters (e.g. shift and tilt) were dramatically reduced in oxidized, *trans*-hemimethylated, and fully methylated duplexes versus oxidized-only and methylated-only controls. Based on our analysis of the base-to-sugar intra-nucleotide distances and an earlier proposal regarding base-to-sugar electrostatic repulsion between O8 and O4′ (56,57), we hypothesize that O8…H2′ steric clash, caused by widening the O8…O4′ distance, can trigger BI → BII transitions 3′ to oxoG.

We also have addressed possible consequences of G oxidation in a methylated CpG context using a plant epigenetic demethylation enzyme, ROS1 5-methylcytosine DNA glycosylase, as a model. Our enzyme kinetics data indicate that ROS1 binds the oxoG-containing strand in the fully methylated oxidized CpG dinucleotide with about fourfold preference over the non-oxidized strand, possibly to avoid mutagenic repair after mC excision with nucleotide incorporation opposite oxoG. In the hemimethylated substrate, the preference for an oxoG-containing substrate is less pronounced but also evident. The lack of a crystal structure of ROS1 with its target DNA prevents direct analysis of the reasons for this oxoG effect; however, since the catalysis step (*k*_cat_) is affected to a lesser extent, it is likely that the preference for oxoG at least partly resides in the initial binding step. This might be either due to DNA destabilization or the dynamics effects within the oxidized methylated CpG site. The use of intrinsic DNA dynamics for initial recognition of a specific DNA target was demonstrated for uracil-DNA glycosylase (67,68) and may be a general principle for this class of enzymes that detect non-canonical bases or base pairs. In a more general framework of protein–ligand recognition, the concept of conformational selection holds that the progress towards a ‘final’ complex, be it a pre-catalytic enzyme–substrate complex or a stable complex of a non-catalytic protein with its ligand, relies on selection of preformed conformers rather than on active distortion of the interacting partners (69,70). Consequently, a shift in the distribution of conformers at the CpG site and the time scale of their interconversion will influence their binding by regulatory proteins. It remains to be seen whether other proteins recognizing methylated CpG dinucleotides, such as methyl-binding domain (MBD-) containing proteins or TET family 5-methylcytosine dioxygenases, are affected by guanine oxidation.

To the best of our knowledge, this study provides the first three-dimensional structure, dynamics and stability analysis of duplex DNA containing two clustered natural base modifications of different origin: base damage (oxoG) and an epigenetic marker (mC). Comparable analyses were carried out with two identical modifications (e.g., abasic sites, mC and 6-methyladenine) clustered within duplex DNA (22,27,71–73). In these cases, the effect of two modifications on the structure and dynamics was also non-additive and, at least for 6-methyladenine, a single modification was more distorting than were two modifications together. An abasic site opposite bulky planar systems such as pyrene or benzo[*a*]pyrene–guanine adduct (74,75) also influence each other's behavior but here the differences with singly modified DNA are obviously driven by the available space inside the base stack rather than by DNA motion. Also, a combination of two abasic sites (76) and oxoG and abasic site separated by two intact base pairs was investigated (77,78), with no obvious crosstalk detected between the lesions, making the combination essentially of non-clustered nature.

In summary, we conclude that clustering of two base modifications (mC and oxoG) within duplex CpG DNA results in a number of local stability, structure and dynamics effects, which could modify the interactions of such DNA with enzymes specifically recognizing methylated CpG dinucleotides and thus have important biological consequences.

## DATA AVAILABILITY

The solution NMR structures of modified DNA duplexes reported in this manuscript (Table 1) are available via the Protein Data Bank (PDB) and linked entries in Biological Magnetic Resonance Data Bank (BMRB). The following PDB entries are available for all the 11 modified DNA duplexes discussed in this work: 5IV1, 5IZP, 5L06, 5L2G, 6ALT, 5UZ1, 5TRN, 6ALU, 5UZ3, 6ALS, 5UZ2.

## Supplementary Material

Supplementary Data
