# Supplementary material for: Oxidative damage to epigenetically methylated sites affects DNA stability, dynamics and enzymatic demethylation
Source: Nucleic Acids Res. 2018 Oct 5;46(20):10827–39. doi: 10.1093/nar/gky893 (PMC6237784; doi:10.1093/nar/gky893)
Supplement: Supplementary Data [file gky893_supplemental_files.pptx]

## Slide 1
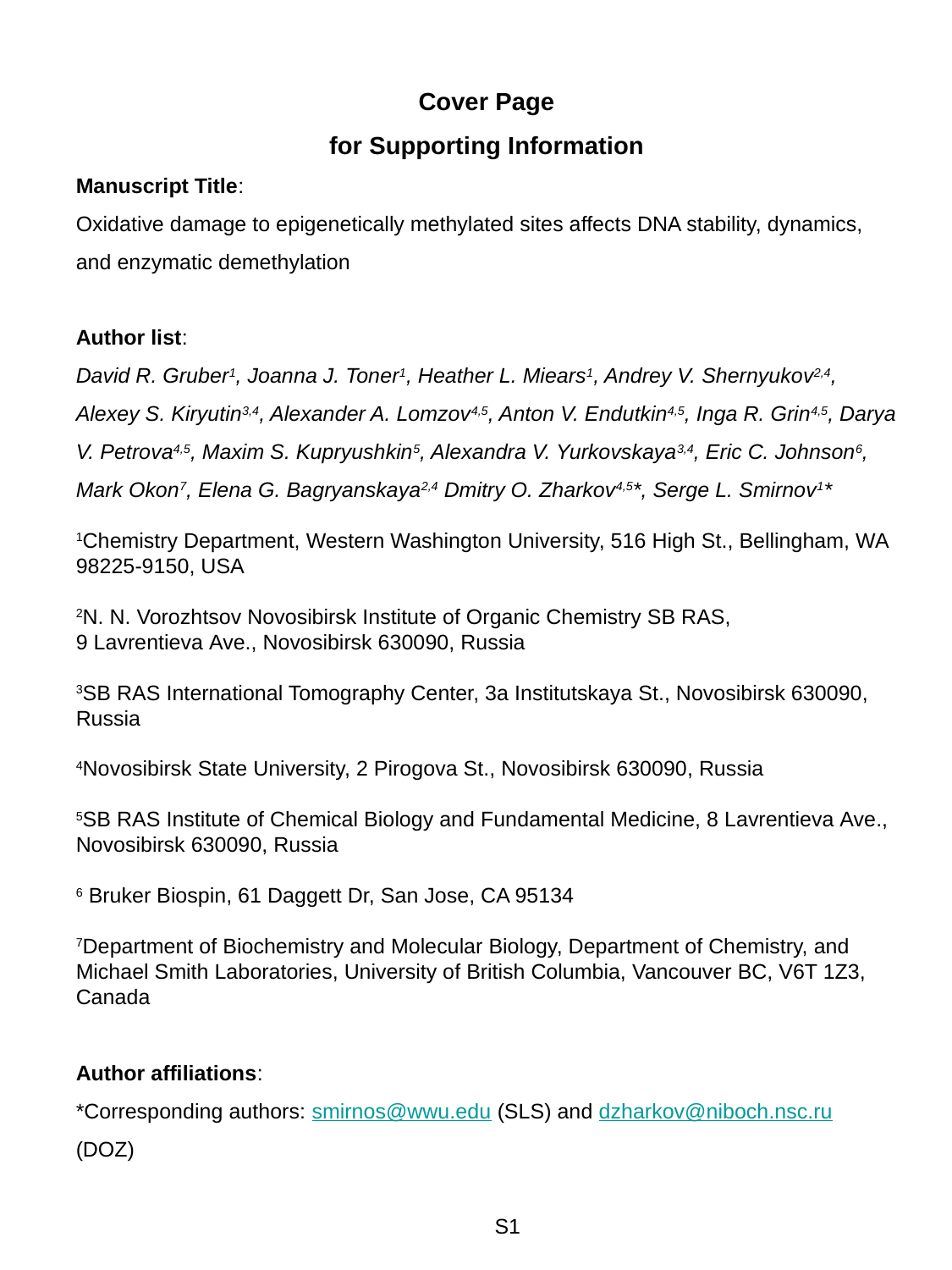

Cover Page
for Supporting Information
Manuscript Title:
Oxidative damage to epigenetically methylated sites affects DNA stability, dynamics, and enzymatic demethylation
Author list:
David R. Gruber1, Joanna J. Toner1, Heather L. Miears1, Andrey V. Shernyukov2,4, Alexey S. Kiryutin3,4, Alexander A. Lomzov4,5, Anton V. Endutkin4,5, Inga R. Grin4,5, Darya V. Petrova4,5, Maxim S. Kupryushkin5, Alexandra V. Yurkovskaya3,4, Eric C. Johnson6, Mark Okon7, Elena G. Bagryanskaya2,4 Dmitry O. Zharkov4,5*, Serge L. Smirnov1*
1Chemistry Department, Western Washington University, 516 High St., Bellingham, WA 98225-9150, USA
2N. N. Vorozhtsov Novosibirsk Institute of Organic Chemistry SB RAS, 9 Lavrentieva Ave., Novosibirsk 630090, Russia
3SB RAS International Tomography Center, 3a Institutskaya St., Novosibirsk 630090, Russia
4Novosibirsk State University, 2 Pirogova St., Novosibirsk 630090, Russia
5SB RAS Institute of Chemical Biology and Fundamental Medicine, 8 Lavrentieva Ave., Novosibirsk 630090, Russia
6 Bruker Biospin, 61 Daggett Dr, San Jose, CA 95134
7Department of Biochemistry and Molecular Biology, Department of Chemistry, and Michael Smith Laboratories, University of British Columbia, Vancouver BC, V6T 1Z3, Canada
Author affiliations:
*Corresponding authors: smirnos@wwu.edu (SLS) and dzharkov@niboch.nsc.ru (DOZ)
S1

## Slide 2
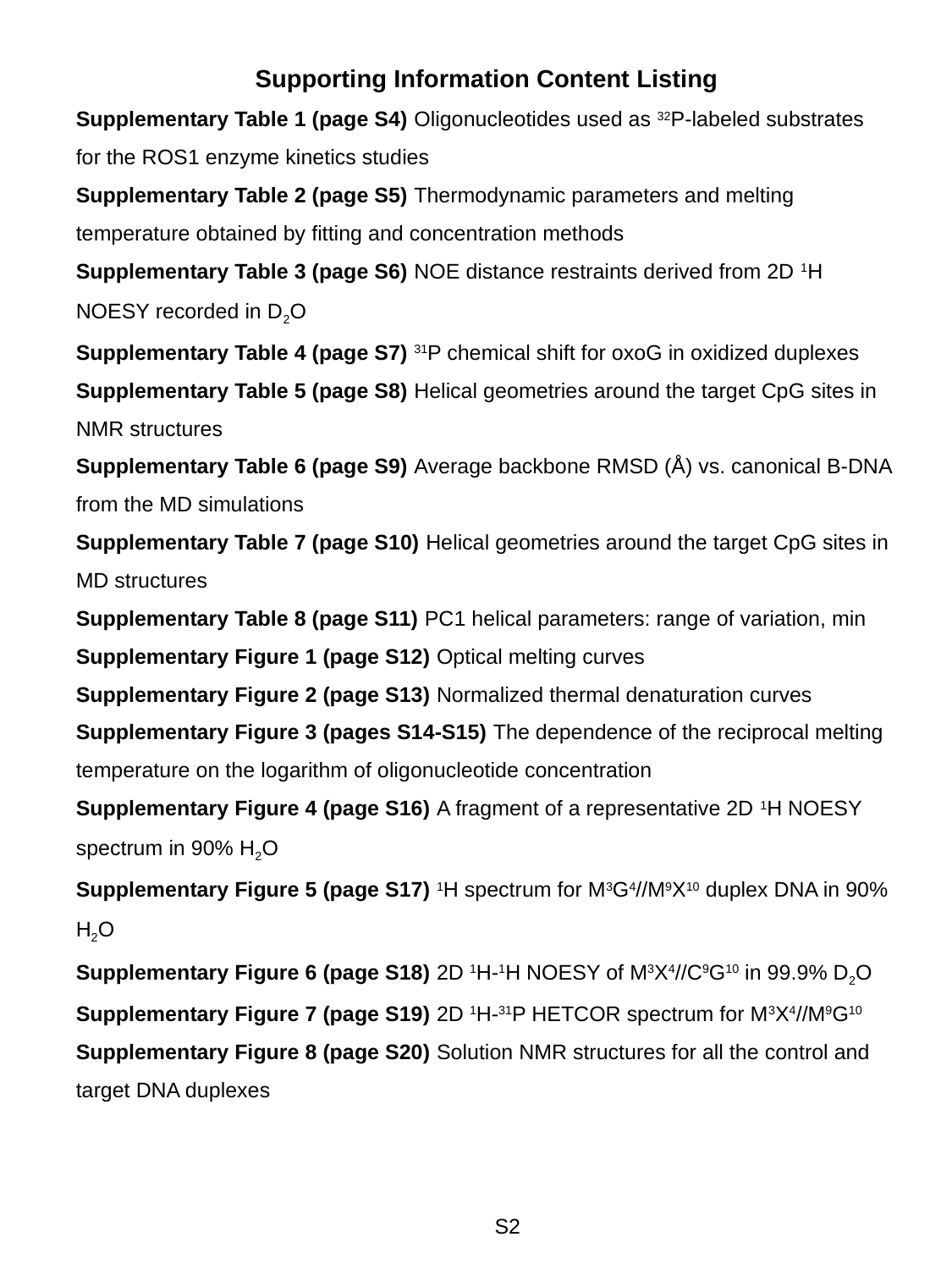

Supporting Information Content Listing
Supplementary Table 1 (page S4) Oligonucleotides used as 32P-labeled substrates
for the ROS1 enzyme kinetics studies
Supplementary Table 2 (page S5) Thermodynamic parameters and melting temperature obtained by fitting and concentration methods
Supplementary Table 3 (page S6) NOE distance restraints derived from 2D 1H NOESY recorded in D2O
Supplementary Table 4 (page S7) 31P chemical shift for oxoG in oxidized duplexes
Supplementary Table 5 (page S8) Helical geometries around the target CpG sites in NMR structures
Supplementary Table 6 (page S9) Average backbone RMSD (Å) vs. canonical B-DNA from the MD simulations
Supplementary Table 7 (page S10) Helical geometries around the target CpG sites in MD structures
Supplementary Table 8 (page S11) PC1 helical parameters: range of variation, min Supplementary Figure 1 (page S12) Optical melting curves
Supplementary Figure 2 (page S13) Normalized thermal denaturation curves
Supplementary Figure 3 (pages S14-S15) The dependence of the reciprocal melting temperature on the logarithm of oligonucleotide concentration
Supplementary Figure 4 (page S16) A fragment of a representative 2D 1H NOESY spectrum in 90% H2O
Supplementary Figure 5 (page S17) 1H spectrum for M3G4//M9X10 duplex DNA in 90% H2O
Supplementary Figure 6 (page S18) 2D 1H-1H NOESY of M3X4//C9G10 in 99.9% D2O
Supplementary Figure 7 (page S19) 2D 1H-31P HETCOR spectrum for M3X4//M9G10
Supplementary Figure 8 (page S20) Solution NMR structures for all the control and target DNA duplexes
S2

## Slide 3
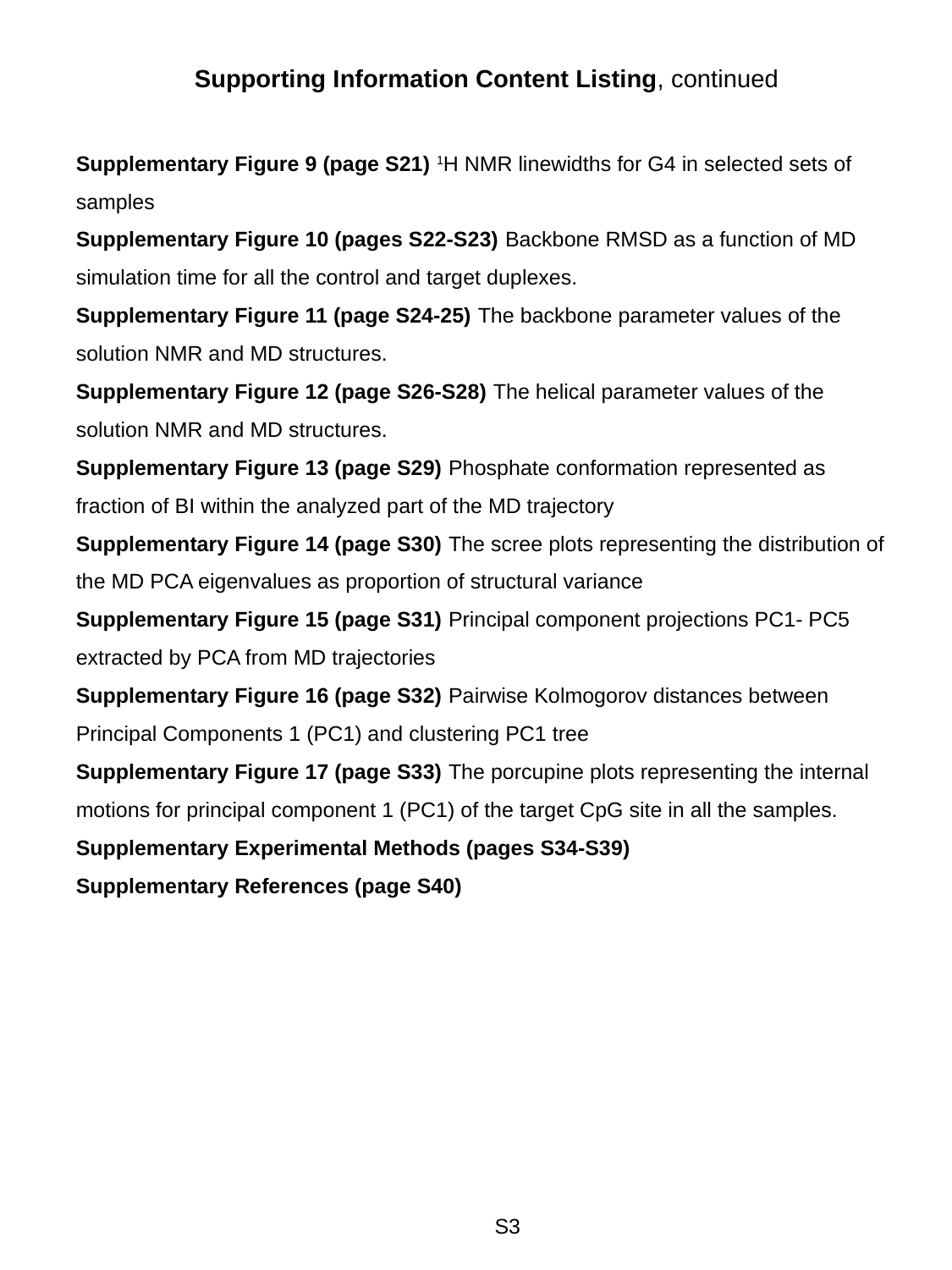

Supporting Information Content Listing, continued
Supplementary Figure 9 (page S21) 1H NMR linewidths for G4 in selected sets of samples
Supplementary Figure 10 (pages S22-S23) Backbone RMSD as a function of MD simulation time for all the control and target duplexes.
Supplementary Figure 11 (page S24-25) The backbone parameter values of the solution NMR and MD structures.
Supplementary Figure 12 (page S26-S28) The helical parameter values of the solution NMR and MD structures.
Supplementary Figure 13 (page S29) Phosphate conformation represented as fraction of BI within the analyzed part of the MD trajectory
Supplementary Figure 14 (page S30) The scree plots representing the distribution of the MD PCA eigenvalues as proportion of structural variance
Supplementary Figure 15 (page S31) Principal component projections PC1- PC5 extracted by PCA from MD trajectories
Supplementary Figure 16 (page S32) Pairwise Kolmogorov distances between Principal Components 1 (PC1) and clustering PC1 tree
Supplementary Figure 17 (page S33) The porcupine plots representing the internal motions for principal component 1 (PC1) of the target CpG site in all the samples.
Supplementary Experimental Methods (pages S34-S39)
Supplementary References (page S40)
S3

## Slide 4
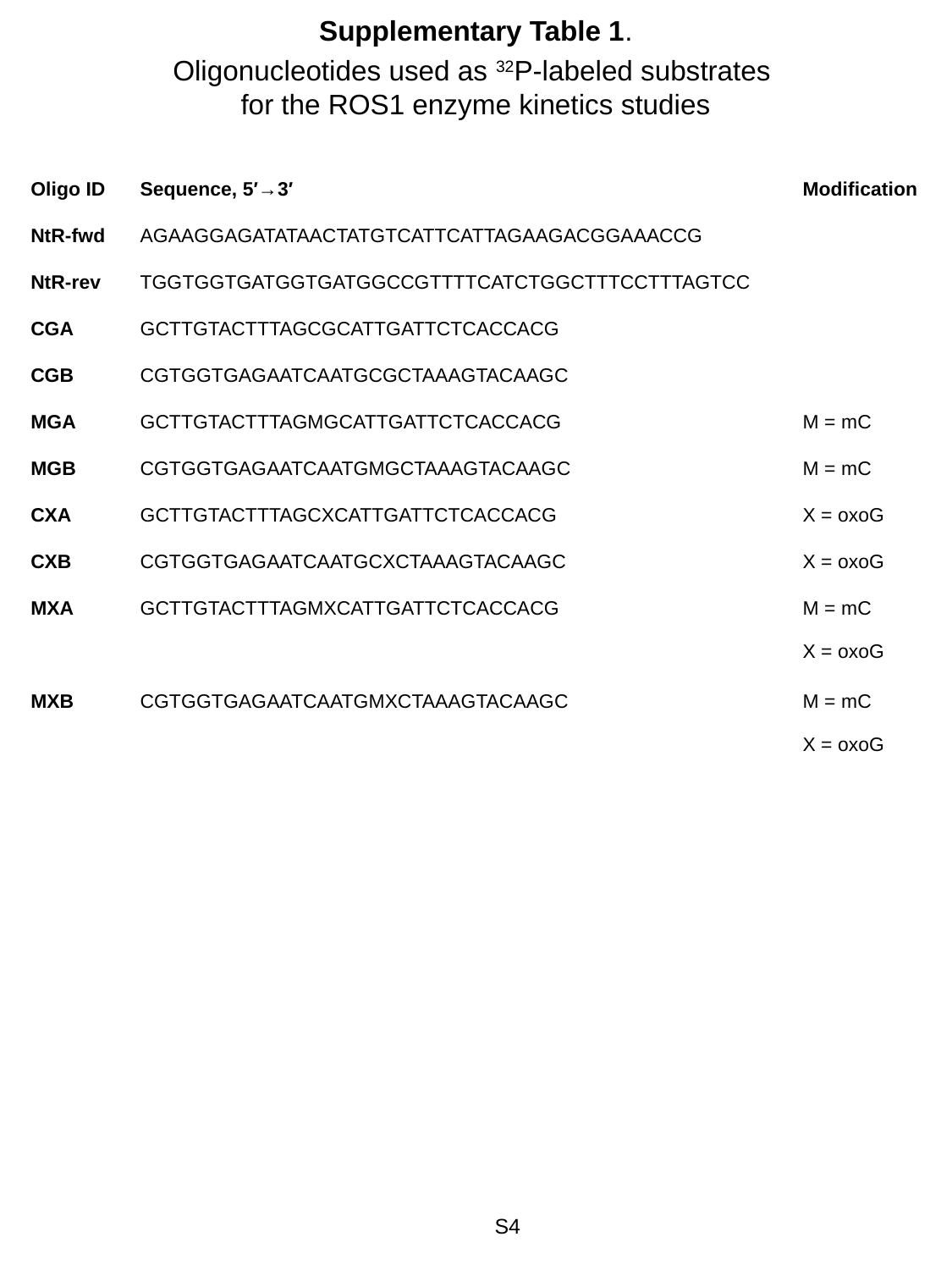

Supplementary Table 1.
Oligonucleotides used as 32P-labeled substrates
for the ROS1 enzyme kinetics studies
| Oligo ID | Sequence, 5′→3′ | Modification |
| --- | --- | --- |
| NtR-fwd | AGAAGGAGATATAACTATGTCATTCATTAGAAGACGGAAACCG | |
| NtR-rev | TGGTGGTGATGGTGATGGCCGTTTTCATCTGGCTTTCCTTTAGTCC | |
| CGA | GCTTGTACTTTAGCGCATTGATTCTCACCACG | |
| CGB | CGTGGTGAGAATCAATGCGCTAAAGTACAAGC | |
| MGA | GCTTGTACTTTAGMGCATTGATTCTCACCACG | M = mC |
| MGB | CGTGGTGAGAATCAATGMGCTAAAGTACAAGC | M = mC |
| CXA | GCTTGTACTTTAGCXCATTGATTCTCACCACG | X = oxoG |
| CXB | CGTGGTGAGAATCAATGCXCTAAAGTACAAGC | X = oxoG |
| MXA | GCTTGTACTTTAGMXCATTGATTCTCACCACG | M = mC X = oxoG |
| MXB | CGTGGTGAGAATCAATGMXCTAAAGTACAAGC | M = mC X = oxoG |
S4

## Slide 5
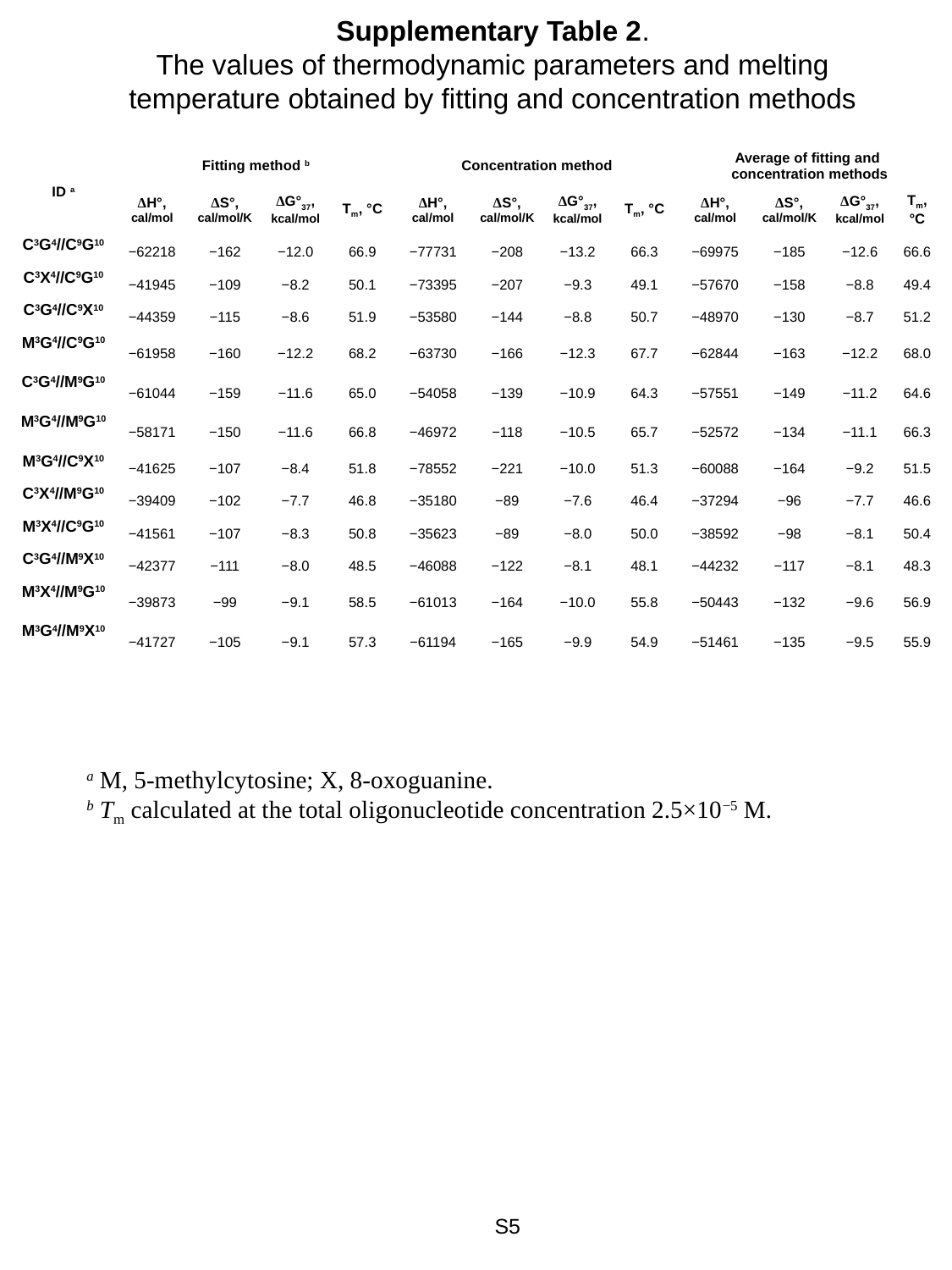

Supplementary Table 2.
The values of thermodynamic parameters and melting temperature obtained by fitting and concentration methods
| ID a | Fitting method b | | | | Concentration method | | | | Average of fitting and concentration methods | | | |
| --- | --- | --- | --- | --- | --- | --- | --- | --- | --- | --- | --- | --- |
| | ΔH°, cal/mol | ΔS°, cal/mol/K | ΔG°37, kcal/mol | Tm, °C | ΔH°, cal/mol | ΔS°, cal/mol/K | ΔG°37, kcal/mol | Tm, °C | ΔH°, cal/mol | ΔS°, cal/mol/K | ΔG°37, kcal/mol | Tm, °C |
| C3G4//C9G10 | −62218 | −162 | −12.0 | 66.9 | −77731 | −208 | −13.2 | 66.3 | −69975 | −185 | −12.6 | 66.6 |
| C3X4//C9G10 | −41945 | −109 | −8.2 | 50.1 | −73395 | −207 | −9.3 | 49.1 | −57670 | −158 | −8.8 | 49.4 |
| C3G4//C9X10 | −44359 | −115 | −8.6 | 51.9 | −53580 | −144 | −8.8 | 50.7 | −48970 | −130 | −8.7 | 51.2 |
| M3G4//C9G10 | −61958 | −160 | −12.2 | 68.2 | −63730 | −166 | −12.3 | 67.7 | −62844 | −163 | −12.2 | 68.0 |
| C3G4//M9G10 | −61044 | −159 | −11.6 | 65.0 | −54058 | −139 | −10.9 | 64.3 | −57551 | −149 | −11.2 | 64.6 |
| M3G4//M9G10 | −58171 | −150 | −11.6 | 66.8 | −46972 | −118 | −10.5 | 65.7 | −52572 | −134 | −11.1 | 66.3 |
| M3G4//C9X10 | −41625 | −107 | −8.4 | 51.8 | −78552 | −221 | −10.0 | 51.3 | −60088 | −164 | −9.2 | 51.5 |
| C3X4//M9G10 | −39409 | −102 | −7.7 | 46.8 | −35180 | −89 | −7.6 | 46.4 | −37294 | −96 | −7.7 | 46.6 |
| M3X4//C9G10 | −41561 | −107 | −8.3 | 50.8 | −35623 | −89 | −8.0 | 50.0 | −38592 | −98 | −8.1 | 50.4 |
| C3G4//M9X10 | −42377 | −111 | −8.0 | 48.5 | −46088 | −122 | −8.1 | 48.1 | −44232 | −117 | −8.1 | 48.3 |
| M3X4//M9G10 | −39873 | −99 | −9.1 | 58.5 | −61013 | −164 | −10.0 | 55.8 | −50443 | −132 | −9.6 | 56.9 |
| M3G4//M9X10 | −41727 | −105 | −9.1 | 57.3 | −61194 | −165 | −9.9 | 54.9 | −51461 | −135 | −9.5 | 55.9 |
a M, 5-methylcytosine; X, 8-oxoguanine.
b Tm calculated at the total oligonucleotide concentration 2.5×10^−5^ M.
S5

## Slide 6
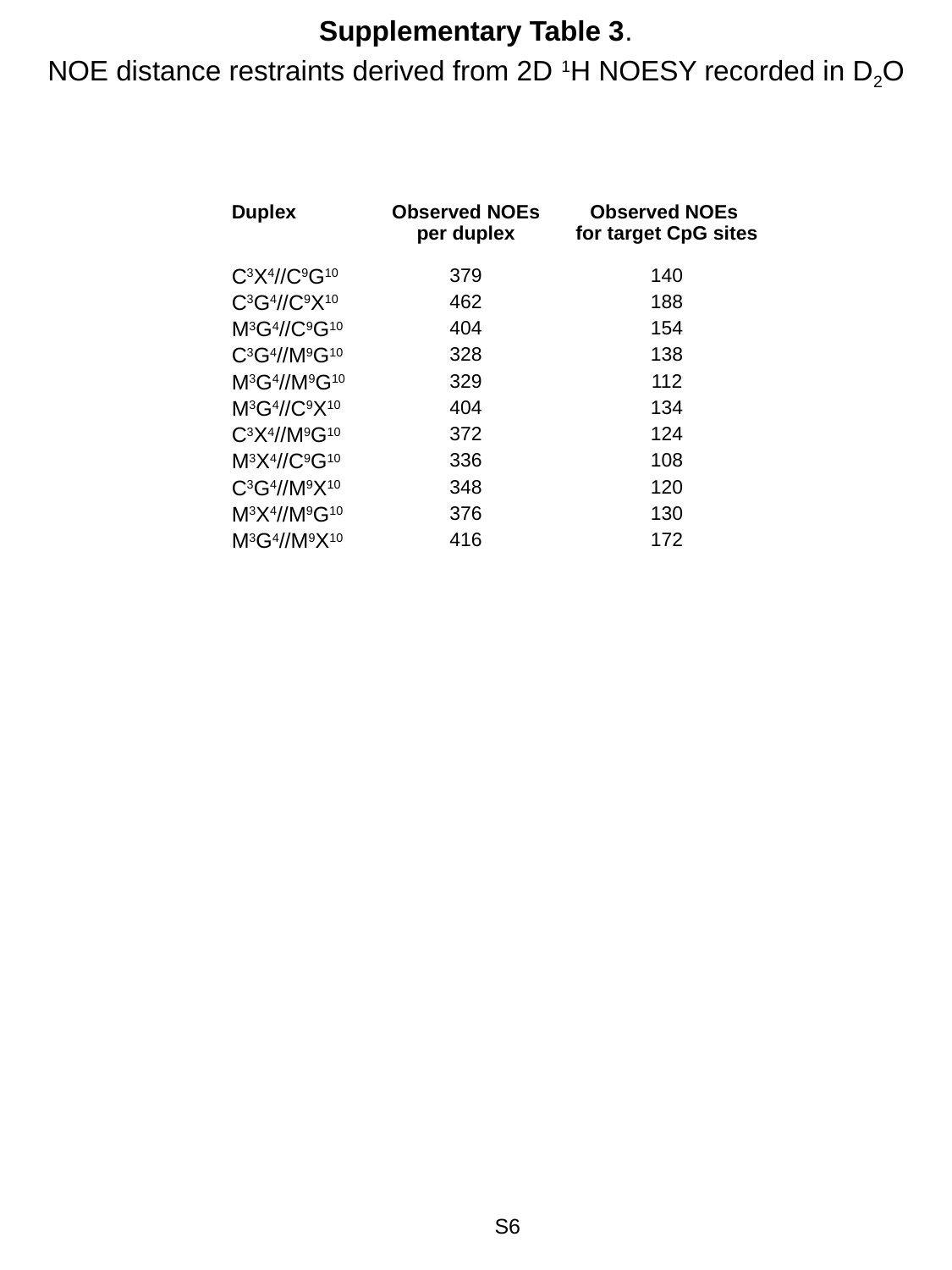

Supplementary Table 3.
NOE distance restraints derived from 2D 1H NOESY recorded in D2O
| Duplex | Observed NOEs per duplex | Observed NOEs for target CpG sites |
| --- | --- | --- |
| C3X4//C9G10 | 379 | 140 |
| C3G4//C9X10 | 462 | 188 |
| M3G4//C9G10 | 404 | 154 |
| C3G4//M9G10 | 328 | 138 |
| M3G4//M9G10 | 329 | 112 |
| M3G4//C9X10 | 404 | 134 |
| C3X4//M9G10 | 372 | 124 |
| M3X4//C9G10 | 336 | 108 |
| C3G4//M9X10 | 348 | 120 |
| M3X4//M9G10 | 376 | 130 |
| M3G4//M9X10 | 416 | 172 |
S6

## Slide 7
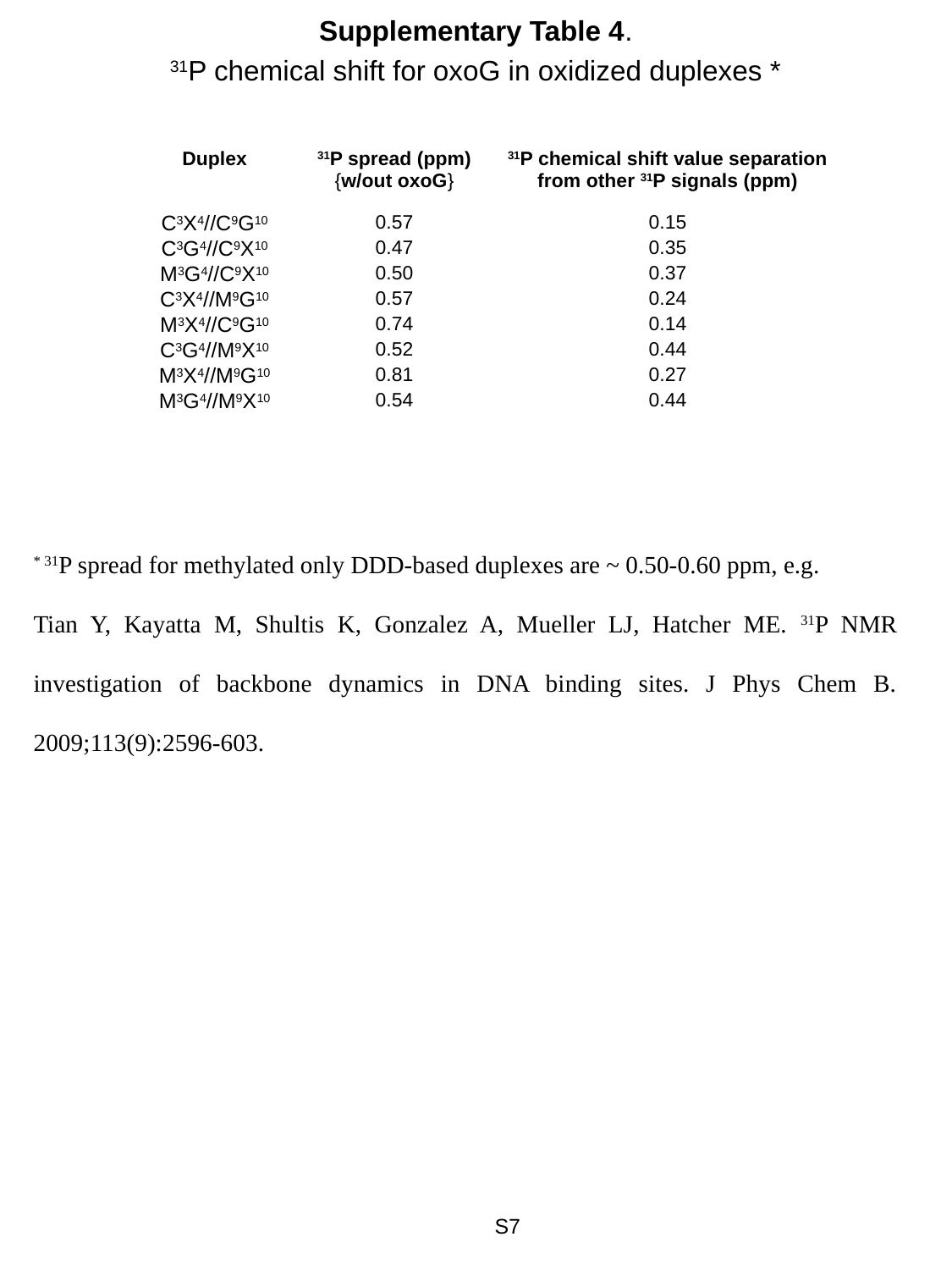

Supplementary Table 4.
31P chemical shift for oxoG in oxidized duplexes *
| Duplex | 31P spread (ppm) {w/out oxoG} | 31P chemical shift value separation from other 31P signals (ppm) |
| --- | --- | --- |
| C3X4//C9G10 | 0.57 | 0.15 |
| C3G4//C9X10 | 0.47 | 0.35 |
| M3G4//C9X10 | 0.50 | 0.37 |
| C3X4//M9G10 | 0.57 | 0.24 |
| M3X4//C9G10 | 0.74 | 0.14 |
| C3G4//M9X10 | 0.52 | 0.44 |
| M3X4//M9G10 | 0.81 | 0.27 |
| M3G4//M9X10 | 0.54 | 0.44 |
* 31P spread for methylated only DDD-based duplexes are ~ 0.50-0.60 ppm, e.g.
Tian Y, Kayatta M, Shultis K, Gonzalez A, Mueller LJ, Hatcher ME. 31P NMR investigation of backbone dynamics in DNA binding sites. J Phys Chem B. 2009;113(9):2596-603.
S7

## Slide 8
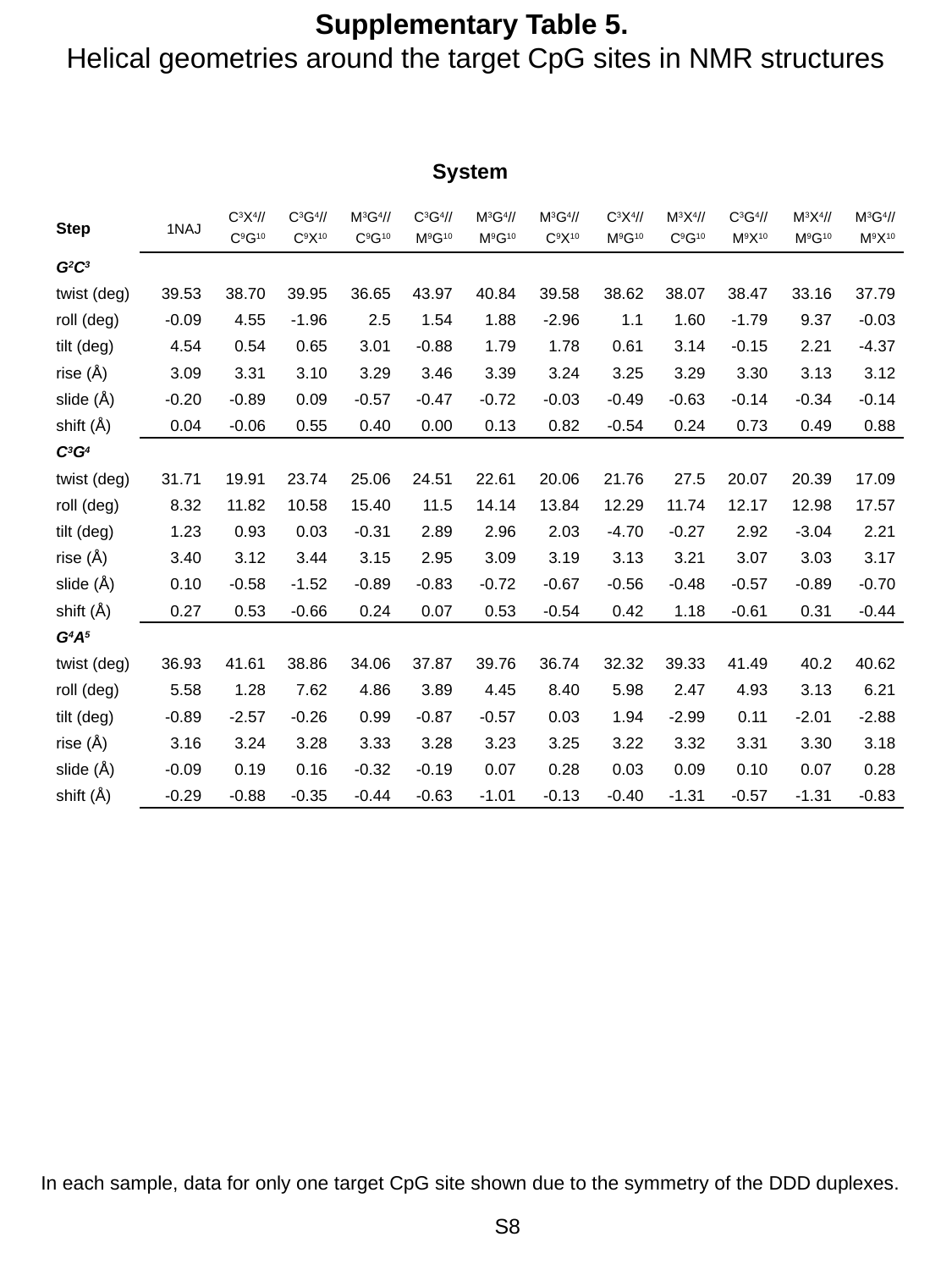

Supplementary Table 5.
Helical geometries around the target CpG sites in NMR structures
System
| Step | 1NAJ | C3X4// C9G10 | C3G4// C9X10 | M3G4// C9G10 | C3G4// M9G10 | M3G4// M9G10 | M3G4// C9X10 | C3X4// M9G10 | M3X4// C9G10 | C3G4// M9X10 | M3X4// M9G10 | M3G4// M9X10 |
| --- | --- | --- | --- | --- | --- | --- | --- | --- | --- | --- | --- | --- |
| G2C3 | | | | | | | | | | | | |
| twist (deg) | 39.53 | 38.70 | 39.95 | 36.65 | 43.97 | 40.84 | 39.58 | 38.62 | 38.07 | 38.47 | 33.16 | 37.79 |
| roll (deg) | -0.09 | 4.55 | -1.96 | 2.5 | 1.54 | 1.88 | -2.96 | 1.1 | 1.60 | -1.79 | 9.37 | -0.03 |
| tilt (deg) | 4.54 | 0.54 | 0.65 | 3.01 | -0.88 | 1.79 | 1.78 | 0.61 | 3.14 | -0.15 | 2.21 | -4.37 |
| rise (Å) | 3.09 | 3.31 | 3.10 | 3.29 | 3.46 | 3.39 | 3.24 | 3.25 | 3.29 | 3.30 | 3.13 | 3.12 |
| slide (Å) | -0.20 | -0.89 | 0.09 | -0.57 | -0.47 | -0.72 | -0.03 | -0.49 | -0.63 | -0.14 | -0.34 | -0.14 |
| shift (Å) | 0.04 | -0.06 | 0.55 | 0.40 | 0.00 | 0.13 | 0.82 | -0.54 | 0.24 | 0.73 | 0.49 | 0.88 |
| C3G4 | | | | | | | | | | | | |
| twist (deg) | 31.71 | 19.91 | 23.74 | 25.06 | 24.51 | 22.61 | 20.06 | 21.76 | 27.5 | 20.07 | 20.39 | 17.09 |
| roll (deg) | 8.32 | 11.82 | 10.58 | 15.40 | 11.5 | 14.14 | 13.84 | 12.29 | 11.74 | 12.17 | 12.98 | 17.57 |
| tilt (deg) | 1.23 | 0.93 | 0.03 | -0.31 | 2.89 | 2.96 | 2.03 | -4.70 | -0.27 | 2.92 | -3.04 | 2.21 |
| rise (Å) | 3.40 | 3.12 | 3.44 | 3.15 | 2.95 | 3.09 | 3.19 | 3.13 | 3.21 | 3.07 | 3.03 | 3.17 |
| slide (Å) | 0.10 | -0.58 | -1.52 | -0.89 | -0.83 | -0.72 | -0.67 | -0.56 | -0.48 | -0.57 | -0.89 | -0.70 |
| shift (Å) | 0.27 | 0.53 | -0.66 | 0.24 | 0.07 | 0.53 | -0.54 | 0.42 | 1.18 | -0.61 | 0.31 | -0.44 |
| G4A5 | | | | | | | | | | | | |
| twist (deg) | 36.93 | 41.61 | 38.86 | 34.06 | 37.87 | 39.76 | 36.74 | 32.32 | 39.33 | 41.49 | 40.2 | 40.62 |
| roll (deg) | 5.58 | 1.28 | 7.62 | 4.86 | 3.89 | 4.45 | 8.40 | 5.98 | 2.47 | 4.93 | 3.13 | 6.21 |
| tilt (deg) | -0.89 | -2.57 | -0.26 | 0.99 | -0.87 | -0.57 | 0.03 | 1.94 | -2.99 | 0.11 | -2.01 | -2.88 |
| rise (Å) | 3.16 | 3.24 | 3.28 | 3.33 | 3.28 | 3.23 | 3.25 | 3.22 | 3.32 | 3.31 | 3.30 | 3.18 |
| slide (Å) | -0.09 | 0.19 | 0.16 | -0.32 | -0.19 | 0.07 | 0.28 | 0.03 | 0.09 | 0.10 | 0.07 | 0.28 |
| shift (Å) | -0.29 | -0.88 | -0.35 | -0.44 | -0.63 | -1.01 | -0.13 | -0.40 | -1.31 | -0.57 | -1.31 | -0.83 |
In each sample, data for only one target CpG site shown due to the symmetry of the DDD duplexes.
S8

## Slide 9
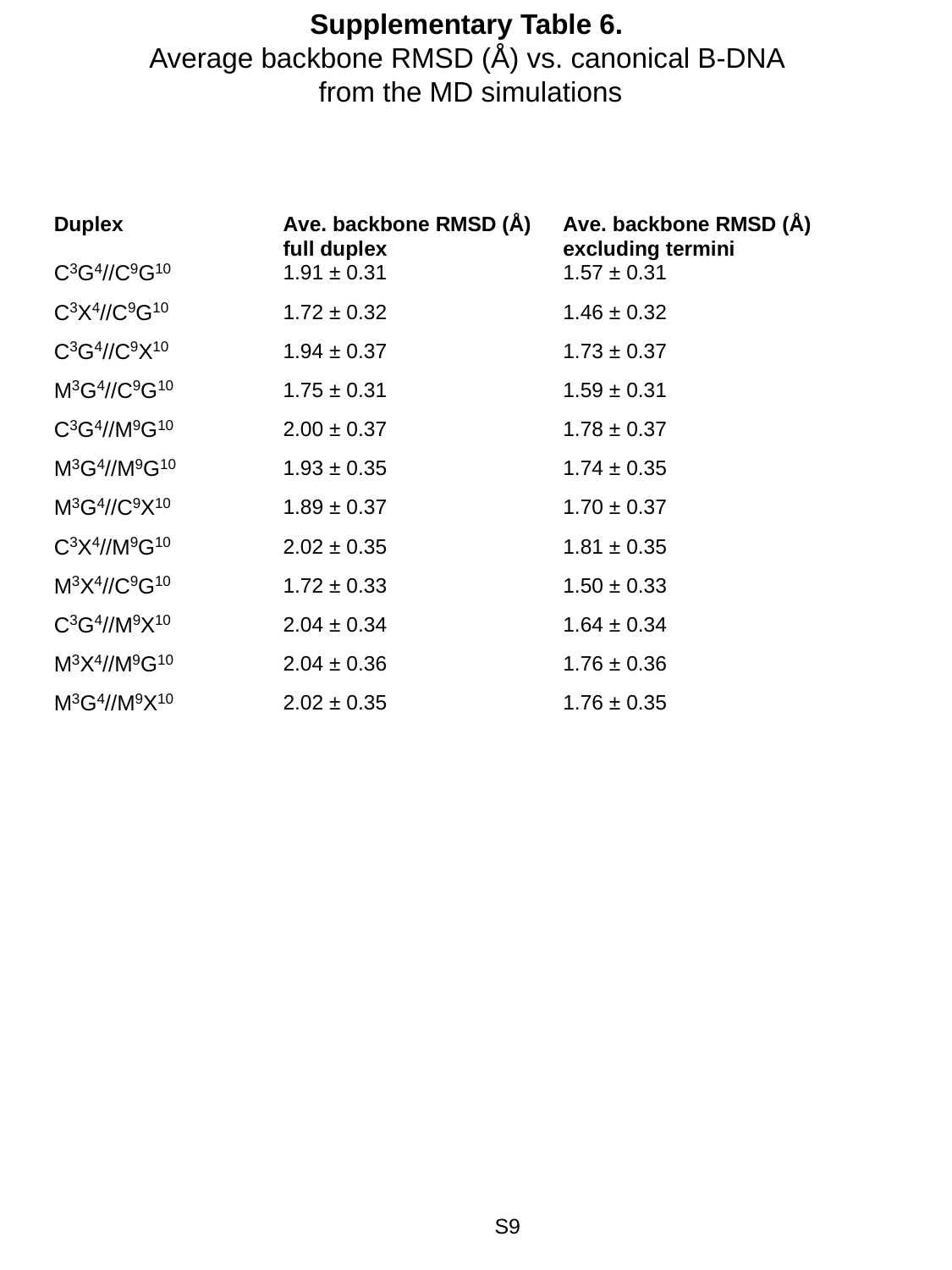

Supplementary Table 6.
Average backbone RMSD (Å) vs. canonical B-DNA
from the MD simulations
S9

## Slide 10
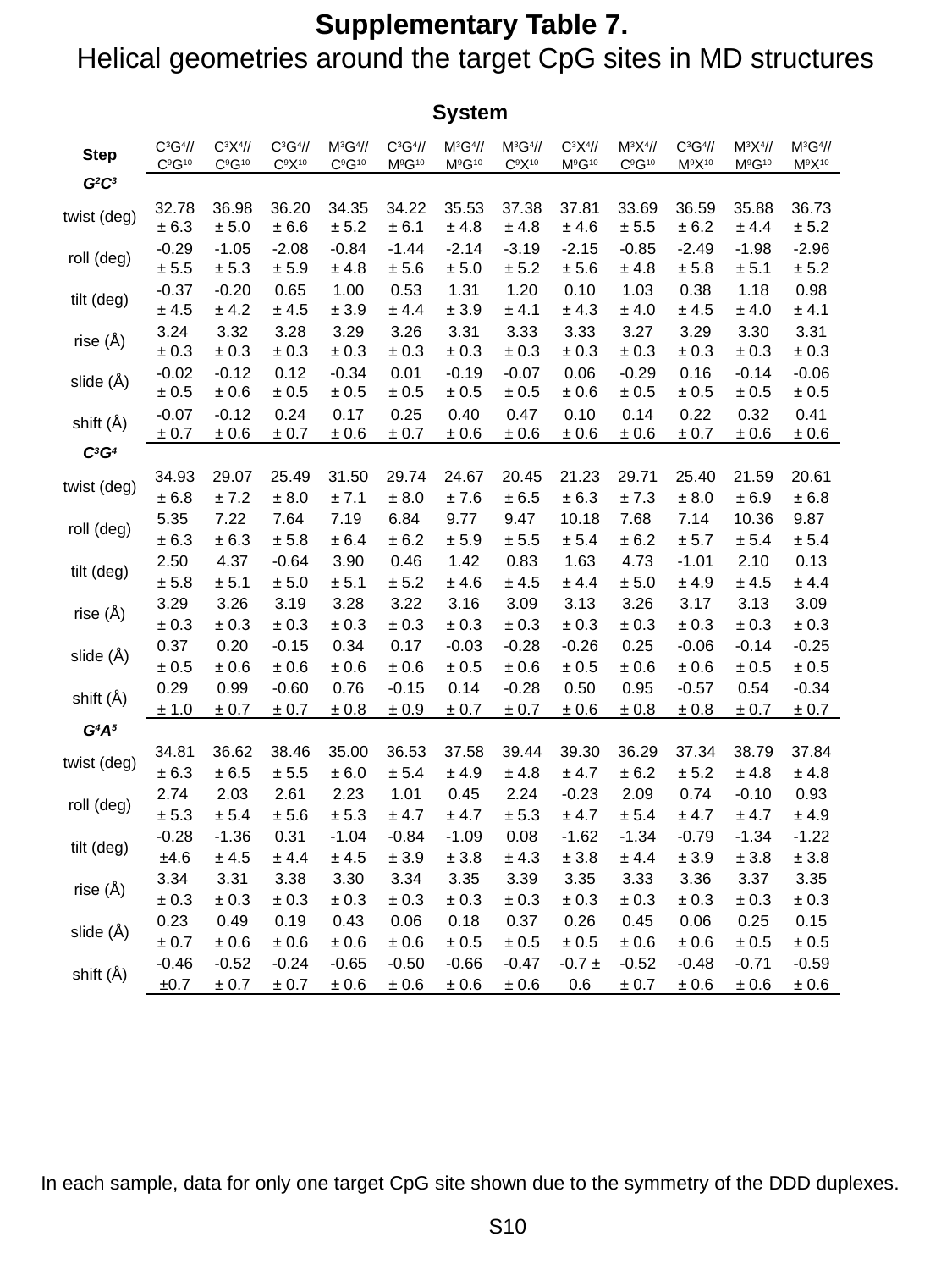

Supplementary Table 7.
Helical geometries around the target CpG sites in MD structures
System
| Step | C3G4// C9G10 | C3X4// C9G10 | C3G4// C9X10 | M3G4// C9G10 | C3G4// M9G10 | M3G4// M9G10 | M3G4// C9X10 | C3X4// M9G10 | M3X4// C9G10 | C3G4// M9X10 | M3X4// M9G10 | M3G4// M9X10 |
| --- | --- | --- | --- | --- | --- | --- | --- | --- | --- | --- | --- | --- |
| G2C3 | | | | | | | | | | | | |
| twist (deg) | 32.78 ± 6.3 | 36.98 ± 5.0 | 36.20 ± 6.6 | 34.35 ± 5.2 | 34.22 ± 6.1 | 35.53 ± 4.8 | 37.38 ± 4.8 | 37.81 ± 4.6 | 33.69 ± 5.5 | 36.59 ± 6.2 | 35.88 ± 4.4 | 36.73 ± 5.2 |
| roll (deg) | -0.29 ± 5.5 | -1.05 ± 5.3 | -2.08 ± 5.9 | -0.84 ± 4.8 | -1.44 ± 5.6 | -2.14 ± 5.0 | -3.19 ± 5.2 | -2.15 ± 5.6 | -0.85 ± 4.8 | -2.49 ± 5.8 | -1.98 ± 5.1 | -2.96 ± 5.2 |
| tilt (deg) | -0.37 ± 4.5 | -0.20 ± 4.2 | 0.65 ± 4.5 | 1.00 ± 3.9 | 0.53 ± 4.4 | 1.31 ± 3.9 | 1.20 ± 4.1 | 0.10 ± 4.3 | 1.03 ± 4.0 | 0.38 ± 4.5 | 1.18 ± 4.0 | 0.98 ± 4.1 |
| rise (Å) | 3.24 ± 0.3 | 3.32 ± 0.3 | 3.28 ± 0.3 | 3.29 ± 0.3 | 3.26 ± 0.3 | 3.31 ± 0.3 | 3.33 ± 0.3 | 3.33 ± 0.3 | 3.27 ± 0.3 | 3.29 ± 0.3 | 3.30 ± 0.3 | 3.31 ± 0.3 |
| slide (Å) | -0.02 ± 0.5 | -0.12 ± 0.6 | 0.12 ± 0.5 | -0.34 ± 0.5 | 0.01 ± 0.5 | -0.19 ± 0.5 | -0.07 ± 0.5 | 0.06 ± 0.6 | -0.29 ± 0.5 | 0.16 ± 0.5 | -0.14 ± 0.5 | -0.06 ± 0.5 |
| shift (Å) | -0.07 ± 0.7 | -0.12 ± 0.6 | 0.24 ± 0.7 | 0.17 ± 0.6 | 0.25 ± 0.7 | 0.40 ± 0.6 | 0.47 ± 0.6 | 0.10 ± 0.6 | 0.14 ± 0.6 | 0.22 ± 0.7 | 0.32 ± 0.6 | 0.41 ± 0.6 |
| C3G4 | | | | | | | | | | | | |
| twist (deg) | 34.93 ± 6.8 | 29.07 ± 7.2 | 25.49 ± 8.0 | 31.50 ± 7.1 | 29.74 ± 8.0 | 24.67 ± 7.6 | 20.45 ± 6.5 | 21.23 ± 6.3 | 29.71 ± 7.3 | 25.40 ± 8.0 | 21.59 ± 6.9 | 20.61 ± 6.8 |
| roll (deg) | 5.35 ± 6.3 | 7.22 ± 6.3 | 7.64 ± 5.8 | 7.19 ± 6.4 | 6.84 ± 6.2 | 9.77 ± 5.9 | 9.47 ± 5.5 | 10.18 ± 5.4 | 7.68 ± 6.2 | 7.14 ± 5.7 | 10.36 ± 5.4 | 9.87 ± 5.4 |
| tilt (deg) | 2.50 ± 5.8 | 4.37 ± 5.1 | -0.64 ± 5.0 | 3.90 ± 5.1 | 0.46 ± 5.2 | 1.42 ± 4.6 | 0.83 ± 4.5 | 1.63 ± 4.4 | 4.73 ± 5.0 | -1.01 ± 4.9 | 2.10 ± 4.5 | 0.13 ± 4.4 |
| rise (Å) | 3.29 ± 0.3 | 3.26 ± 0.3 | 3.19 ± 0.3 | 3.28 ± 0.3 | 3.22 ± 0.3 | 3.16 ± 0.3 | 3.09 ± 0.3 | 3.13 ± 0.3 | 3.26 ± 0.3 | 3.17 ± 0.3 | 3.13 ± 0.3 | 3.09 ± 0.3 |
| slide (Å) | 0.37 ± 0.5 | 0.20 ± 0.6 | -0.15 ± 0.6 | 0.34 ± 0.6 | 0.17 ± 0.6 | -0.03 ± 0.5 | -0.28 ± 0.6 | -0.26 ± 0.5 | 0.25 ± 0.6 | -0.06 ± 0.6 | -0.14 ± 0.5 | -0.25 ± 0.5 |
| shift (Å) | 0.29 ± 1.0 | 0.99 ± 0.7 | -0.60 ± 0.7 | 0.76 ± 0.8 | -0.15 ± 0.9 | 0.14 ± 0.7 | -0.28 ± 0.7 | 0.50 ± 0.6 | 0.95 ± 0.8 | -0.57 ± 0.8 | 0.54 ± 0.7 | -0.34 ± 0.7 |
| G4A5 | | | | | | | | | | | | |
| twist (deg) | 34.81 ± 6.3 | 36.62 ± 6.5 | 38.46 ± 5.5 | 35.00 ± 6.0 | 36.53 ± 5.4 | 37.58 ± 4.9 | 39.44 ± 4.8 | 39.30 ± 4.7 | 36.29 ± 6.2 | 37.34 ± 5.2 | 38.79 ± 4.8 | 37.84 ± 4.8 |
| roll (deg) | 2.74 ± 5.3 | 2.03 ± 5.4 | 2.61 ± 5.6 | 2.23 ± 5.3 | 1.01 ± 4.7 | 0.45 ± 4.7 | 2.24 ± 5.3 | -0.23 ± 4.7 | 2.09 ± 5.4 | 0.74 ± 4.7 | -0.10 ± 4.7 | 0.93 ± 4.9 |
| tilt (deg) | -0.28 ±4.6 | -1.36 ± 4.5 | 0.31 ± 4.4 | -1.04 ± 4.5 | -0.84 ± 3.9 | -1.09 ± 3.8 | 0.08 ± 4.3 | -1.62 ± 3.8 | -1.34 ± 4.4 | -0.79 ± 3.9 | -1.34 ± 3.8 | -1.22 ± 3.8 |
| rise (Å) | 3.34 ± 0.3 | 3.31 ± 0.3 | 3.38 ± 0.3 | 3.30 ± 0.3 | 3.34 ± 0.3 | 3.35 ± 0.3 | 3.39 ± 0.3 | 3.35 ± 0.3 | 3.33 ± 0.3 | 3.36 ± 0.3 | 3.37 ± 0.3 | 3.35 ± 0.3 |
| slide (Å) | 0.23 ± 0.7 | 0.49 ± 0.6 | 0.19 ± 0.6 | 0.43 ± 0.6 | 0.06 ± 0.6 | 0.18 ± 0.5 | 0.37 ± 0.5 | 0.26 ± 0.5 | 0.45 ± 0.6 | 0.06 ± 0.6 | 0.25 ± 0.5 | 0.15 ± 0.5 |
| shift (Å) | -0.46 ±0.7 | -0.52 ± 0.7 | -0.24 ± 0.7 | -0.65 ± 0.6 | -0.50 ± 0.6 | -0.66 ± 0.6 | -0.47 ± 0.6 | -0.7 ± 0.6 | -0.52 ± 0.7 | -0.48 ± 0.6 | -0.71 ± 0.6 | -0.59 ± 0.6 |
In each sample, data for only one target CpG site shown due to the symmetry of the DDD duplexes.
S10

## Slide 11
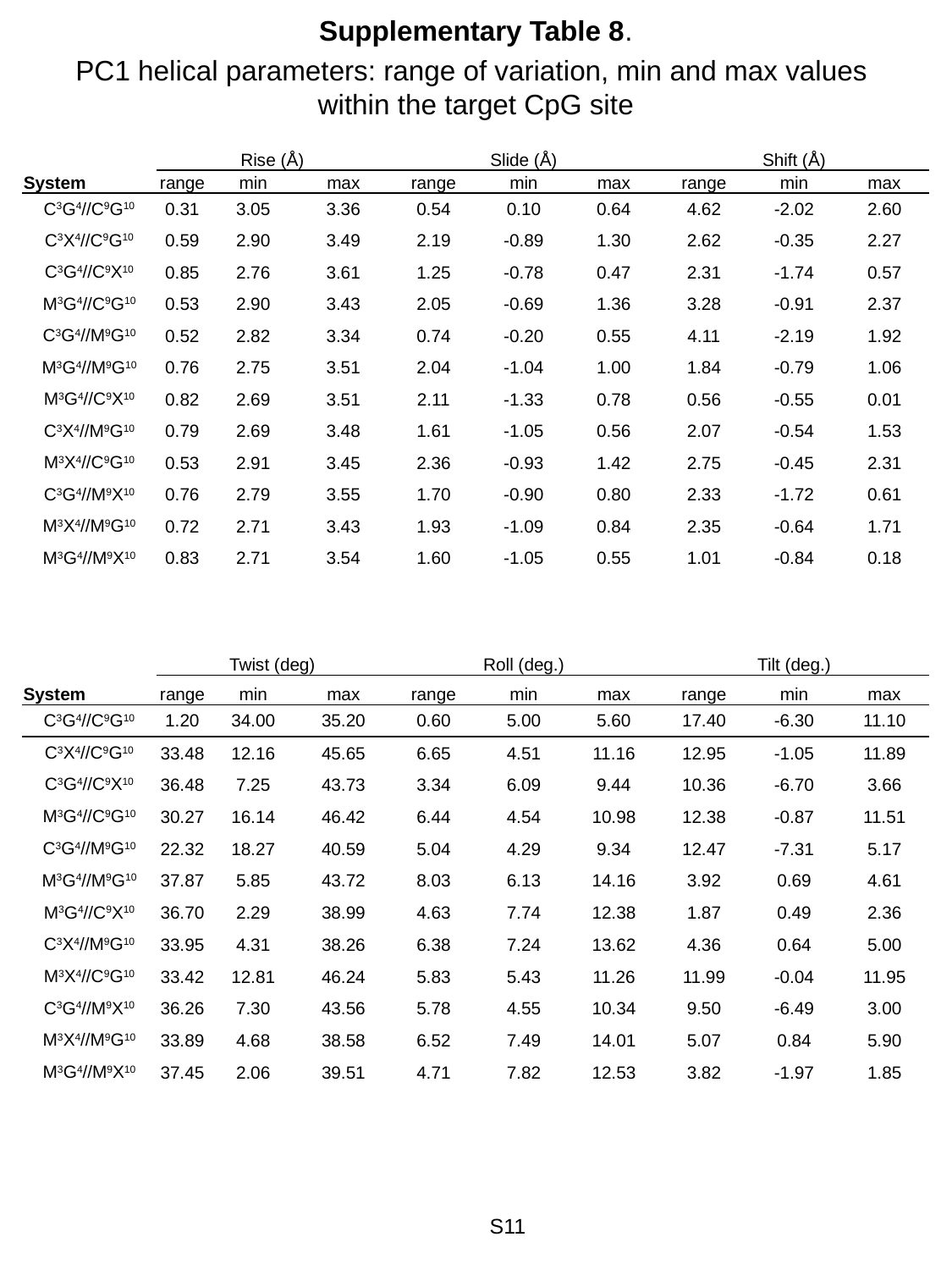

Supplementary Table 8.
PC1 helical parameters: range of variation, min and max values
within the target CpG site
| | Rise (Å) | | | Slide (Å) | | | Shift (Å) | | |
| --- | --- | --- | --- | --- | --- | --- | --- | --- | --- |
| System | range | min | max | range | min | max | range | min | max |
| C3G4//C9G10 | 0.31 | 3.05 | 3.36 | 0.54 | 0.10 | 0.64 | 4.62 | -2.02 | 2.60 |
| C3X4//C9G10 | 0.59 | 2.90 | 3.49 | 2.19 | -0.89 | 1.30 | 2.62 | -0.35 | 2.27 |
| C3G4//C9X10 | 0.85 | 2.76 | 3.61 | 1.25 | -0.78 | 0.47 | 2.31 | -1.74 | 0.57 |
| M3G4//C9G10 | 0.53 | 2.90 | 3.43 | 2.05 | -0.69 | 1.36 | 3.28 | -0.91 | 2.37 |
| C3G4//M9G10 | 0.52 | 2.82 | 3.34 | 0.74 | -0.20 | 0.55 | 4.11 | -2.19 | 1.92 |
| M3G4//M9G10 | 0.76 | 2.75 | 3.51 | 2.04 | -1.04 | 1.00 | 1.84 | -0.79 | 1.06 |
| M3G4//C9X10 | 0.82 | 2.69 | 3.51 | 2.11 | -1.33 | 0.78 | 0.56 | -0.55 | 0.01 |
| C3X4//M9G10 | 0.79 | 2.69 | 3.48 | 1.61 | -1.05 | 0.56 | 2.07 | -0.54 | 1.53 |
| M3X4//C9G10 | 0.53 | 2.91 | 3.45 | 2.36 | -0.93 | 1.42 | 2.75 | -0.45 | 2.31 |
| C3G4//M9X10 | 0.76 | 2.79 | 3.55 | 1.70 | -0.90 | 0.80 | 2.33 | -1.72 | 0.61 |
| M3X4//M9G10 | 0.72 | 2.71 | 3.43 | 1.93 | -1.09 | 0.84 | 2.35 | -0.64 | 1.71 |
| M3G4//M9X10 | 0.83 | 2.71 | 3.54 | 1.60 | -1.05 | 0.55 | 1.01 | -0.84 | 0.18 |
| | Twist (deg) | | | Roll (deg.) | | | Tilt (deg.) | | |
| --- | --- | --- | --- | --- | --- | --- | --- | --- | --- |
| System | range | min | max | range | min | max | range | min | max |
| C3G4//C9G10 | 1.20 | 34.00 | 35.20 | 0.60 | 5.00 | 5.60 | 17.40 | -6.30 | 11.10 |
| C3X4//C9G10 | 33.48 | 12.16 | 45.65 | 6.65 | 4.51 | 11.16 | 12.95 | -1.05 | 11.89 |
| C3G4//C9X10 | 36.48 | 7.25 | 43.73 | 3.34 | 6.09 | 9.44 | 10.36 | -6.70 | 3.66 |
| M3G4//C9G10 | 30.27 | 16.14 | 46.42 | 6.44 | 4.54 | 10.98 | 12.38 | -0.87 | 11.51 |
| C3G4//M9G10 | 22.32 | 18.27 | 40.59 | 5.04 | 4.29 | 9.34 | 12.47 | -7.31 | 5.17 |
| M3G4//M9G10 | 37.87 | 5.85 | 43.72 | 8.03 | 6.13 | 14.16 | 3.92 | 0.69 | 4.61 |
| M3G4//C9X10 | 36.70 | 2.29 | 38.99 | 4.63 | 7.74 | 12.38 | 1.87 | 0.49 | 2.36 |
| C3X4//M9G10 | 33.95 | 4.31 | 38.26 | 6.38 | 7.24 | 13.62 | 4.36 | 0.64 | 5.00 |
| M3X4//C9G10 | 33.42 | 12.81 | 46.24 | 5.83 | 5.43 | 11.26 | 11.99 | -0.04 | 11.95 |
| C3G4//M9X10 | 36.26 | 7.30 | 43.56 | 5.78 | 4.55 | 10.34 | 9.50 | -6.49 | 3.00 |
| M3X4//M9G10 | 33.89 | 4.68 | 38.58 | 6.52 | 7.49 | 14.01 | 5.07 | 0.84 | 5.90 |
| M3G4//M9X10 | 37.45 | 2.06 | 39.51 | 4.71 | 7.82 | 12.53 | 3.82 | -1.97 | 1.85 |
S11

## Slide 12
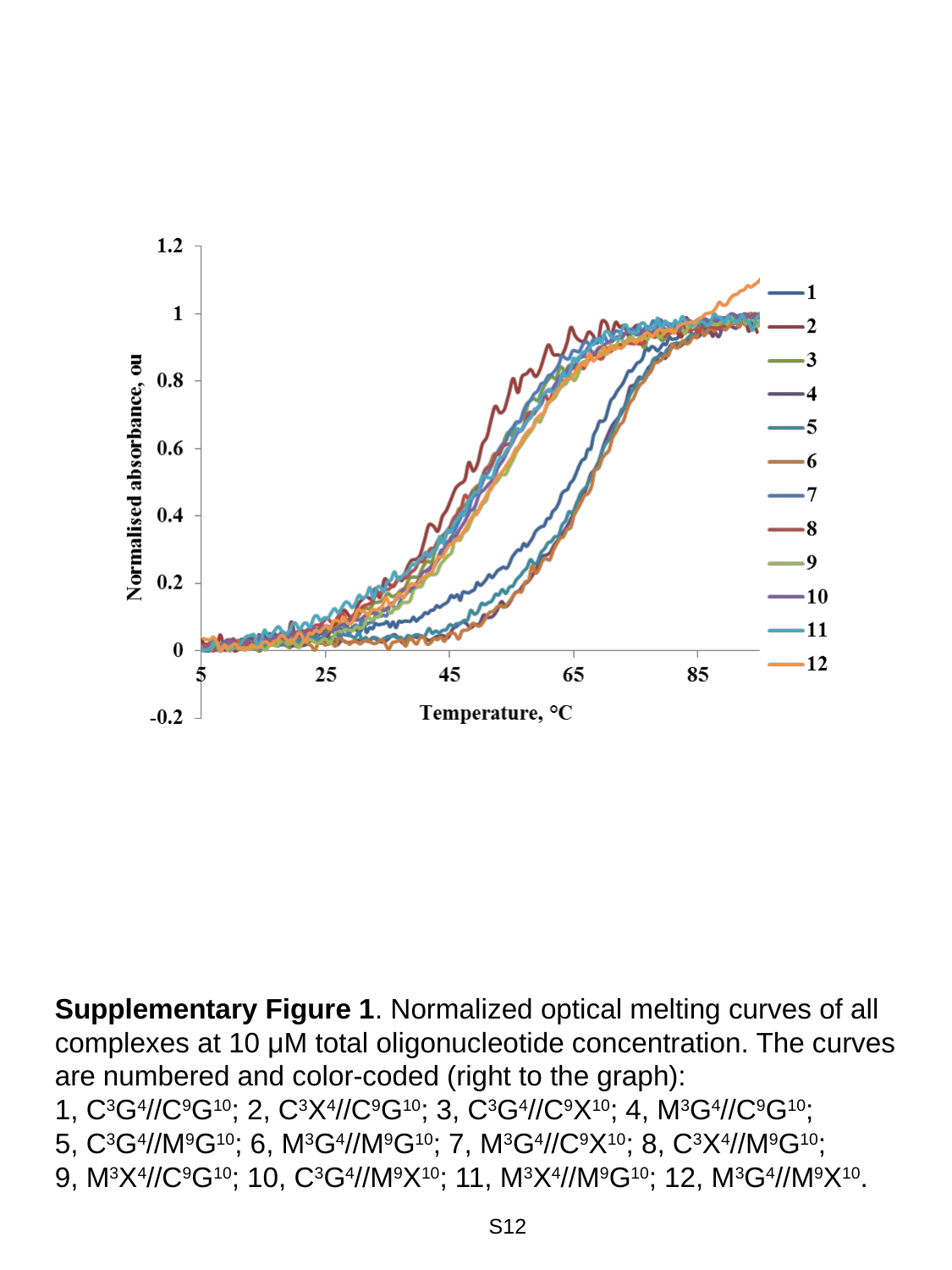

Supplementary Figure 1. Normalized optical melting curves of all complexes at 10 μM total oligonucleotide concentration. The curves are numbered and color-coded (right to the graph):
1, C3G4//C9G10; 2, C3X4//C9G10; 3, C3G4//C9X10; 4, M3G4//C9G10;
5, C3G4//M9G10; 6, M3G4//M9G10; 7, M3G4//C9X10; 8, C3X4//M9G10;
9, M3X4//C9G10; 10, C3G4//M9X10; 11, M3X4//M9G10; 12, M3G4//M9X10.
S12

## Slide 13
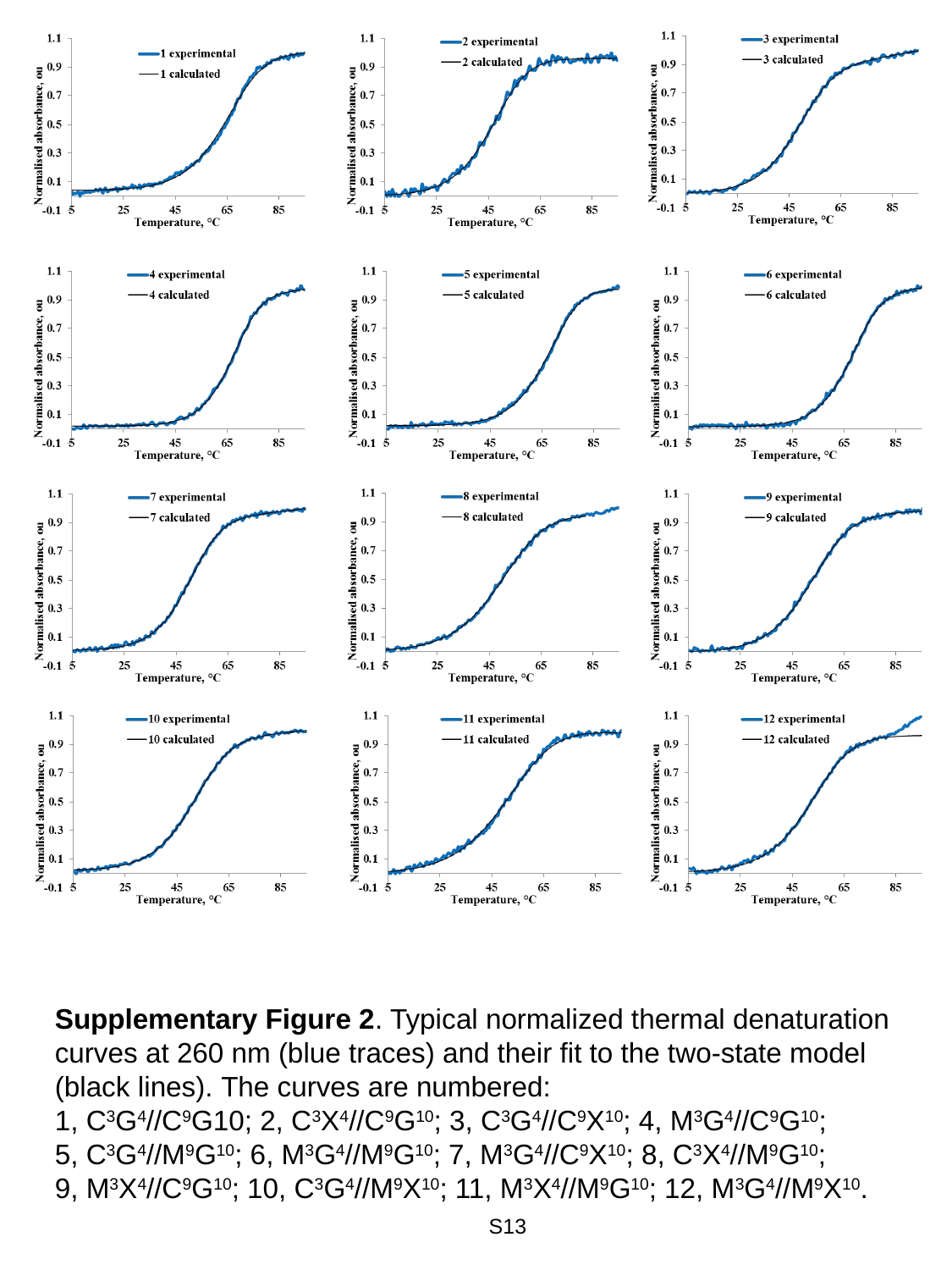

Supplementary Figure 2. Typical normalized thermal denaturation curves at 260 nm (blue traces) and their fit to the two-state model (black lines). The curves are numbered:
1, C3G4//C9G10; 2, C3X4//C9G10; 3, C3G4//C9X10; 4, M3G4//C9G10;
5, C3G4//M9G10; 6, M3G4//M9G10; 7, M3G4//C9X10; 8, C3X4//M9G10;
9, M3X4//C9G10; 10, C3G4//M9X10; 11, M3X4//M9G10; 12, M3G4//M9X10.
S13

## Slide 14
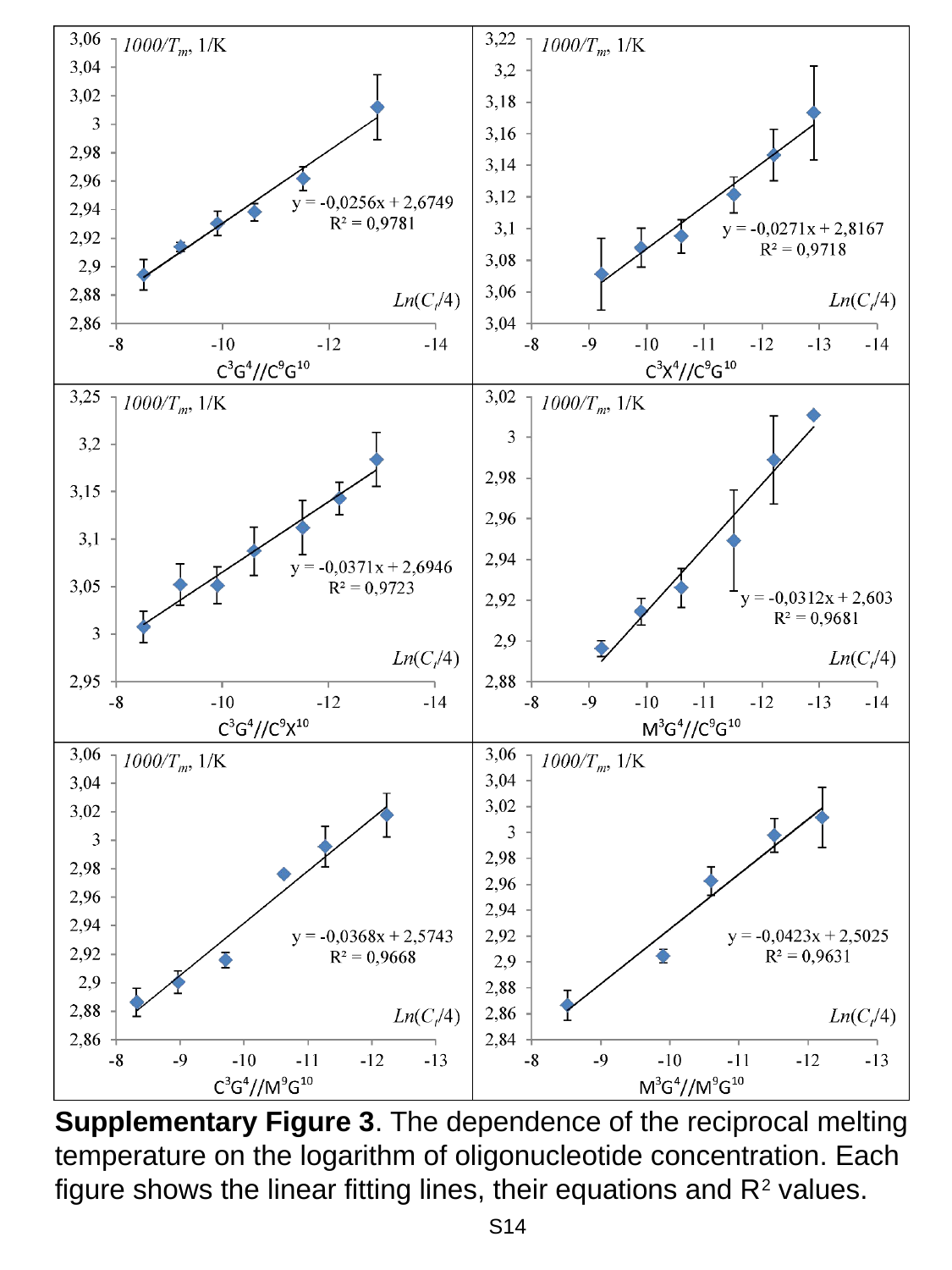

Supplementary Figure 3. The dependence of the reciprocal melting temperature on the logarithm of oligonucleotide concentration. Each figure shows the linear fitting lines, their equations and R2 values.
S14

## Slide 15
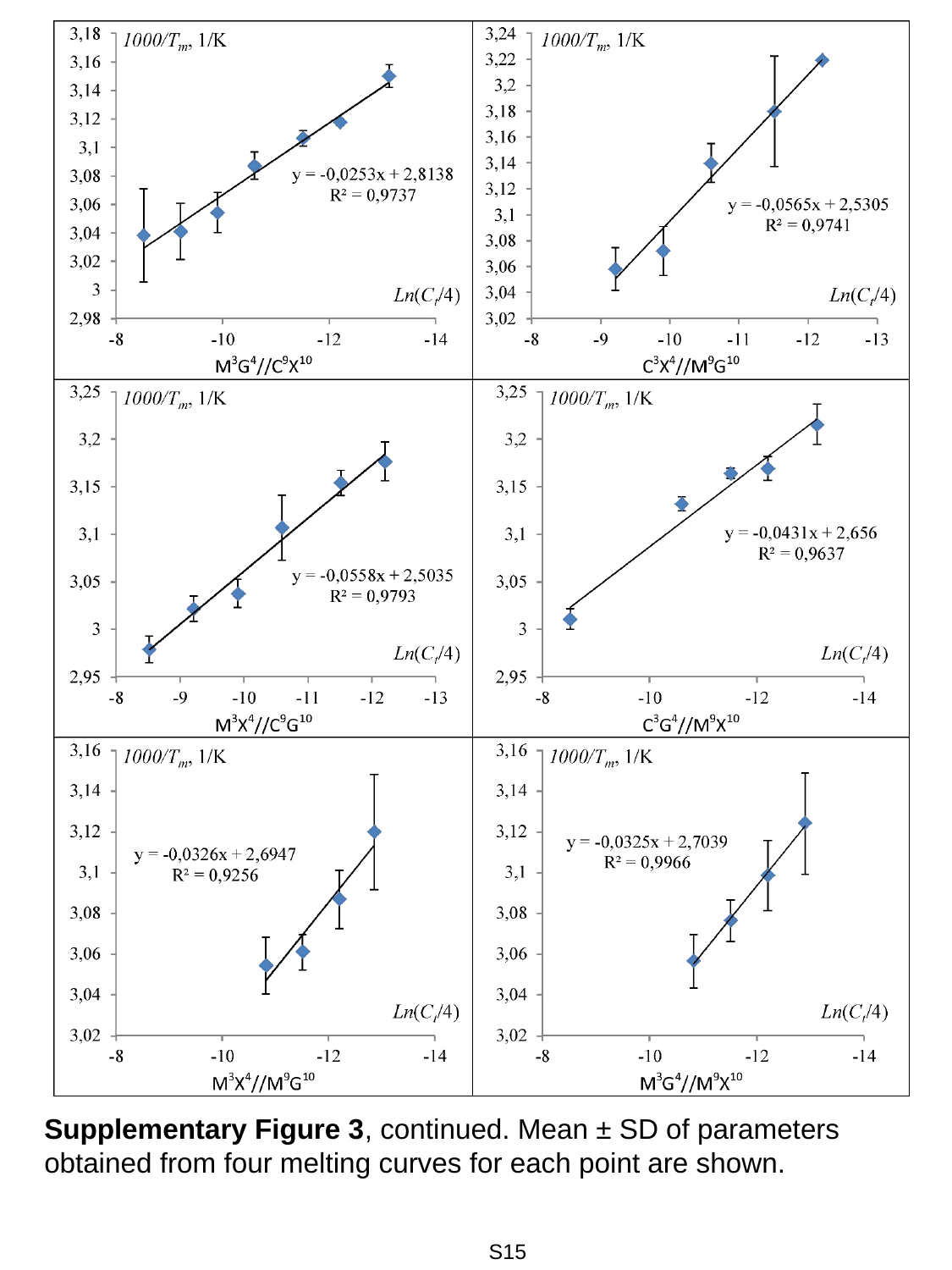

Supplementary Figure 3, continued. Mean ± SD of parameters obtained from four melting curves for each point are shown.
S15

## Slide 16
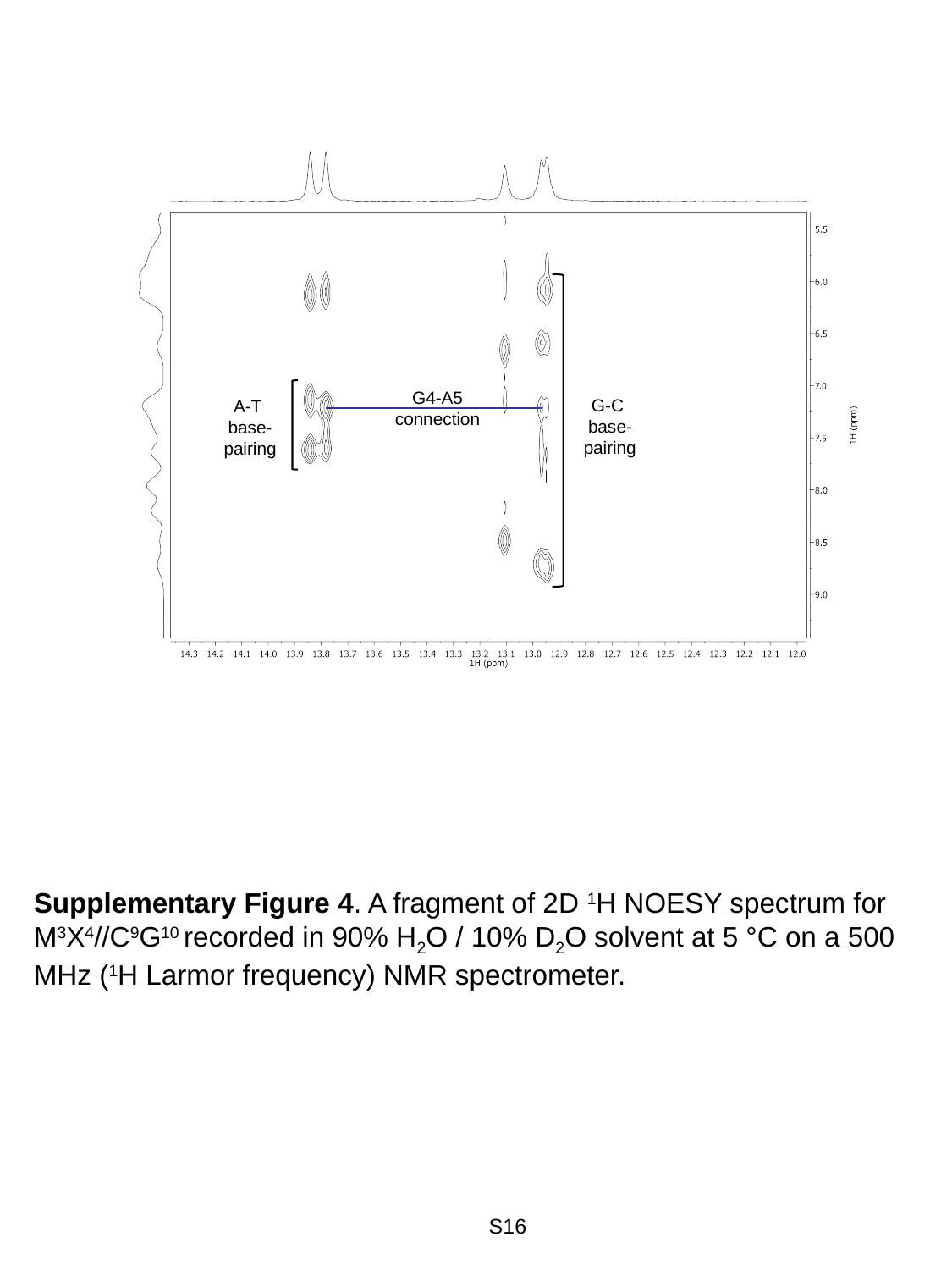

G4-A5 connection
G-C
base-pairing
A-T
base-pairing
Supplementary Figure 4. A fragment of 2D 1H NOESY spectrum for M3X4//C9G10 recorded in 90% H2O / 10% D2O solvent at 5 °C on a 500 MHz (1H Larmor frequency) NMR spectrometer.
S16

## Slide 17
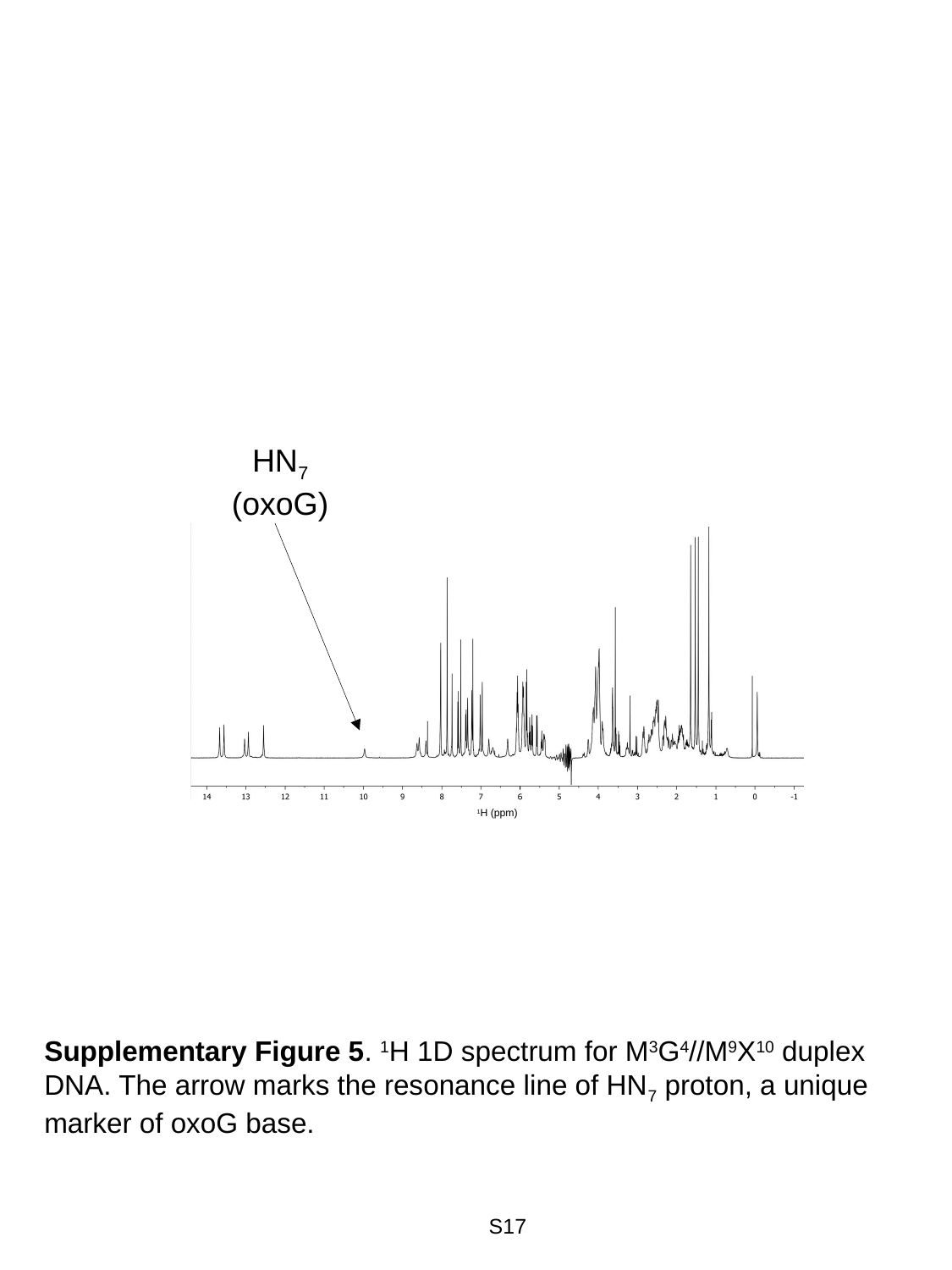

HN7 (oxoG)
1H (ppm)
Supplementary Figure 5. 1H 1D spectrum for M3G4//M9X10 duplex DNA. The arrow marks the resonance line of HN7 proton, a unique marker of oxoG base.
S17

## Slide 18
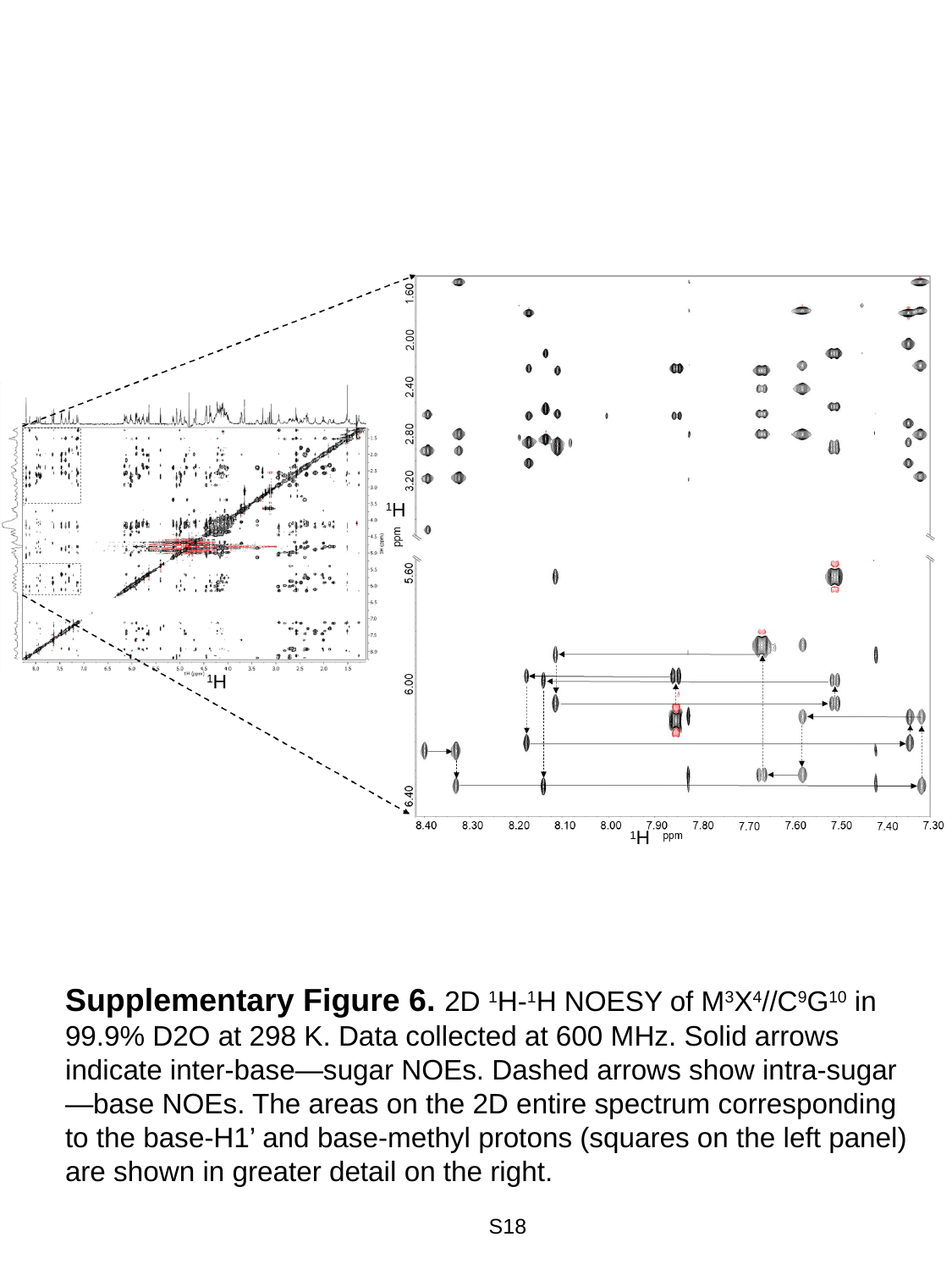

1H
1H
1H
Supplementary Figure 6. 2D 1H-1H NOESY of M3X4//C9G10 in 99.9% D2O at 298 K. Data collected at 600 MHz. Solid arrows indicate inter-base—sugar NOEs. Dashed arrows show intra-sugar—base NOEs. The areas on the 2D entire spectrum corresponding to the base-H1’ and base-methyl protons (squares on the left panel) are shown in greater detail on the right.
S18

## Slide 19
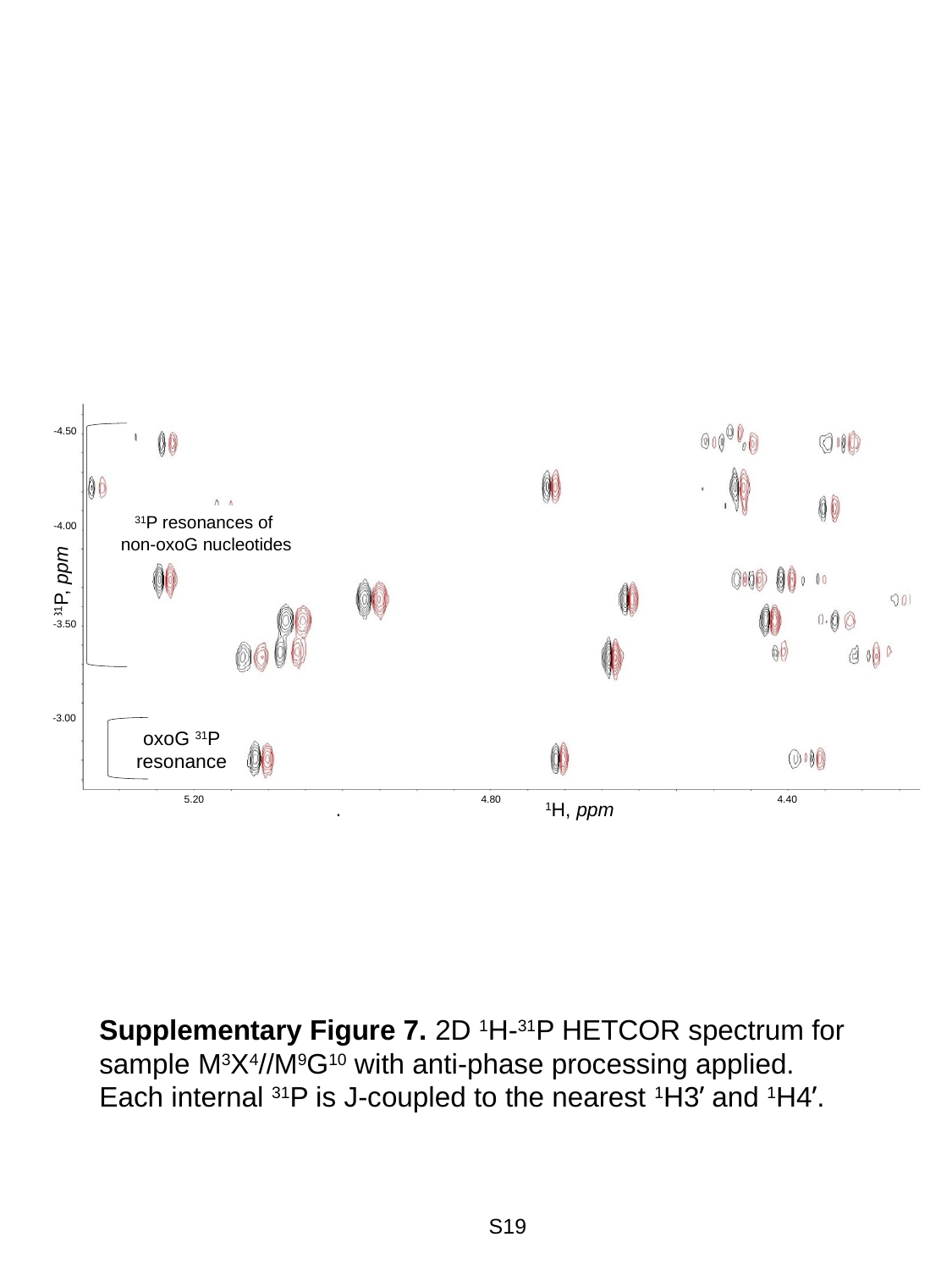

-4.50
31P resonances of
non-oxoG nucleotides
-4.00
31P, ppm
-3.50
-3.00
oxoG 31P
resonance
5.20
4.80
4.40
1H, ppm
.
Supplementary Figure 7. 2D 1H-31P HETCOR spectrum for sample M3X4//M9G10 with anti-phase processing applied. Each internal 31P is J-coupled to the nearest 1H3’ and 1H4’.
S19

## Slide 20
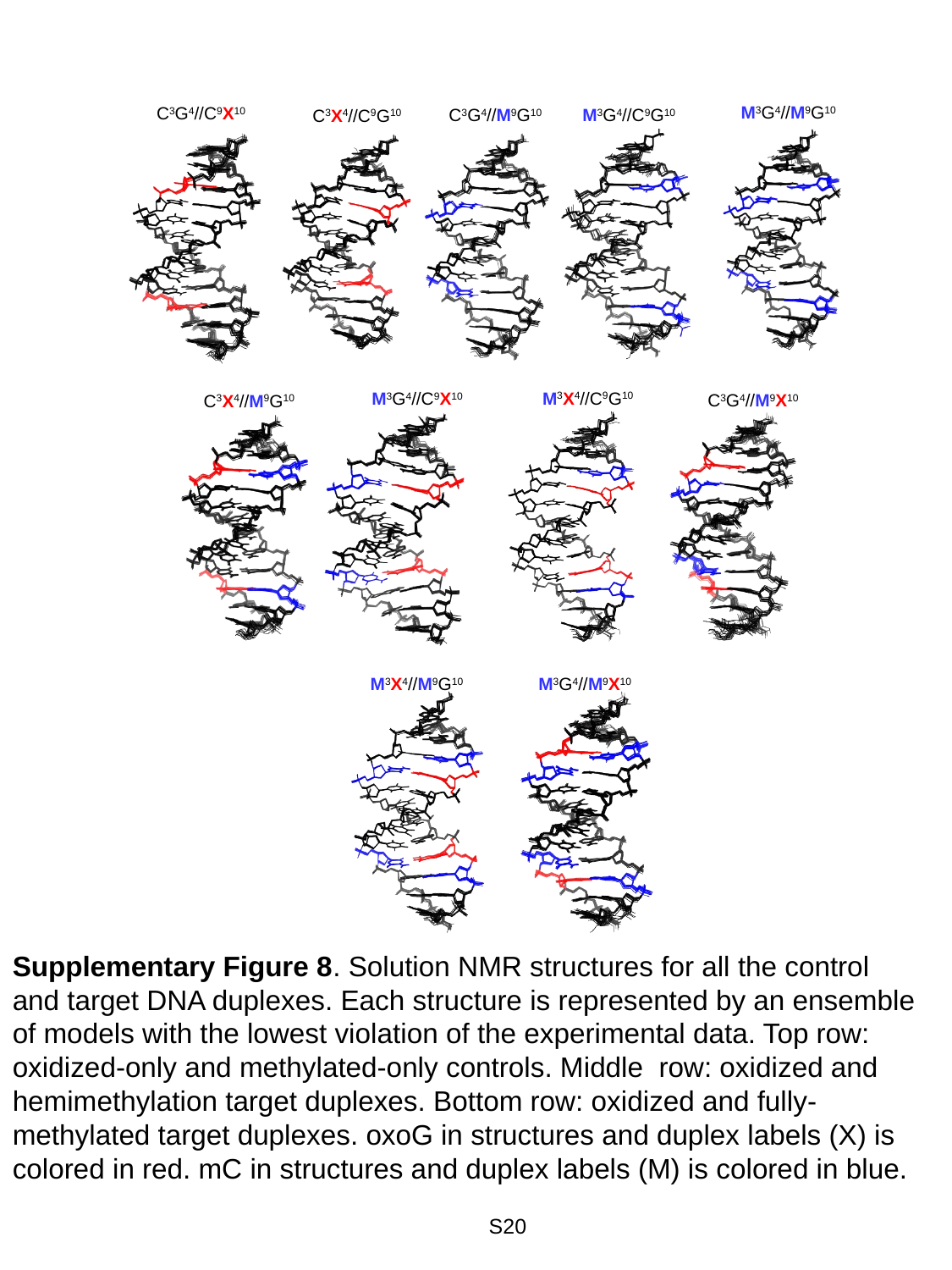

M3G4//M9G10
C3G4//C9X10
C3G4//M9G10
M3G4//C9G10
C3X4//C9G10
M3X4//C9G10
M3G4//C9X10
C3G4//M9X10
C3X4//M9G10
M3G4//M9X10
M3X4//M9G10
Supplementary Figure 8. Solution NMR structures for all the control and target DNA duplexes. Each structure is represented by an ensemble of models with the lowest violation of the experimental data. Top row: oxidized-only and methylated-only controls. Middle row: oxidized and hemimethylation target duplexes. Bottom row: oxidized and fully-methylated target duplexes. oxoG in structures and duplex labels (X) is colored in red. mC in structures and duplex labels (M) is colored in blue.
S20

## Slide 21
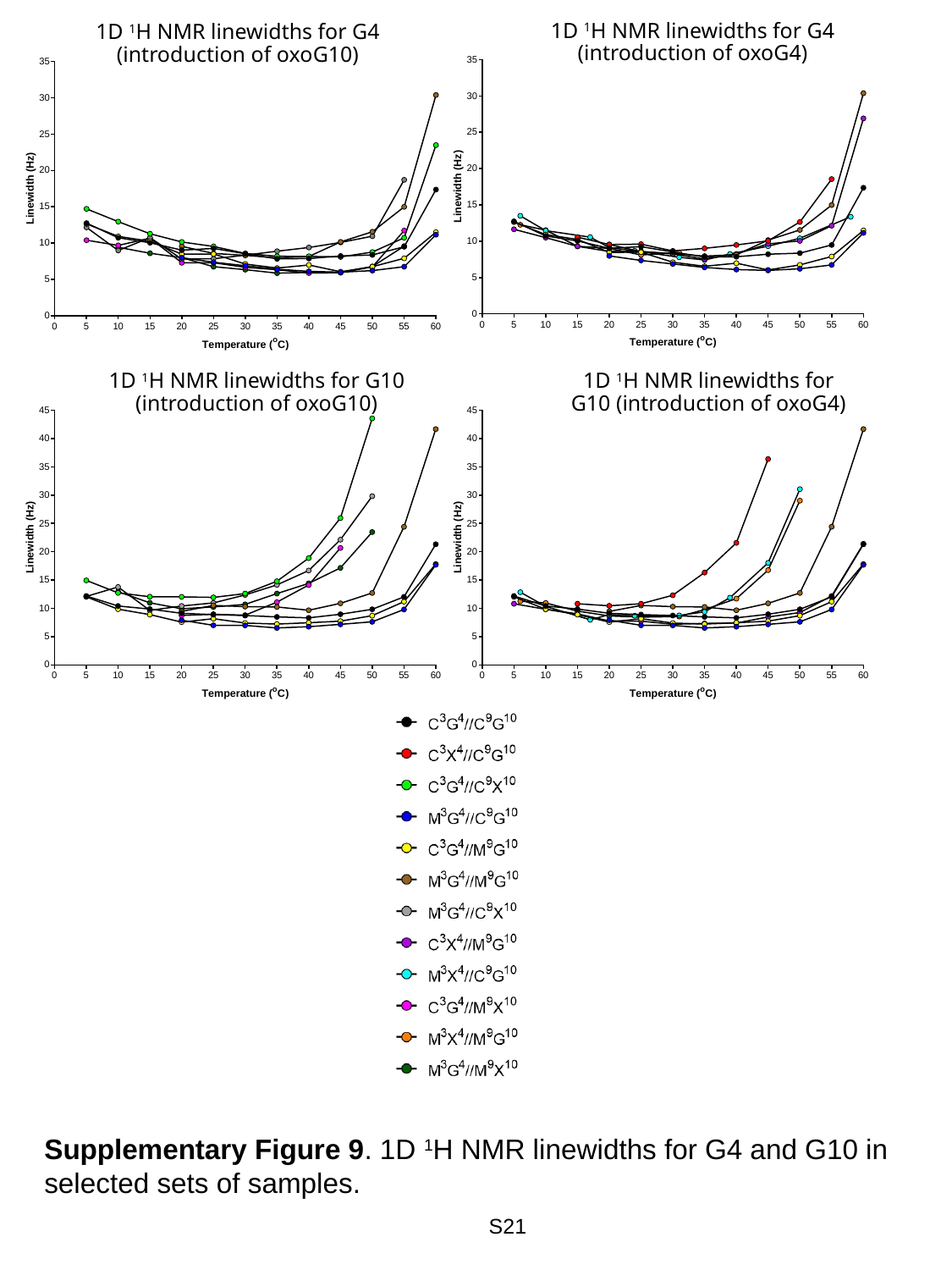

1D 1H NMR linewidths for G4 (introduction of oxoG10)
1D 1H NMR linewidths for G4 (introduction of oxoG4)
1D 1H NMR linewidths for G10 (introduction of oxoG10)
1D 1H NMR linewidths for G10 (introduction of oxoG4)
Supplementary Figure 9. 1D 1H NMR linewidths for G4 and G10 in selected sets of samples.
S21

## Slide 22
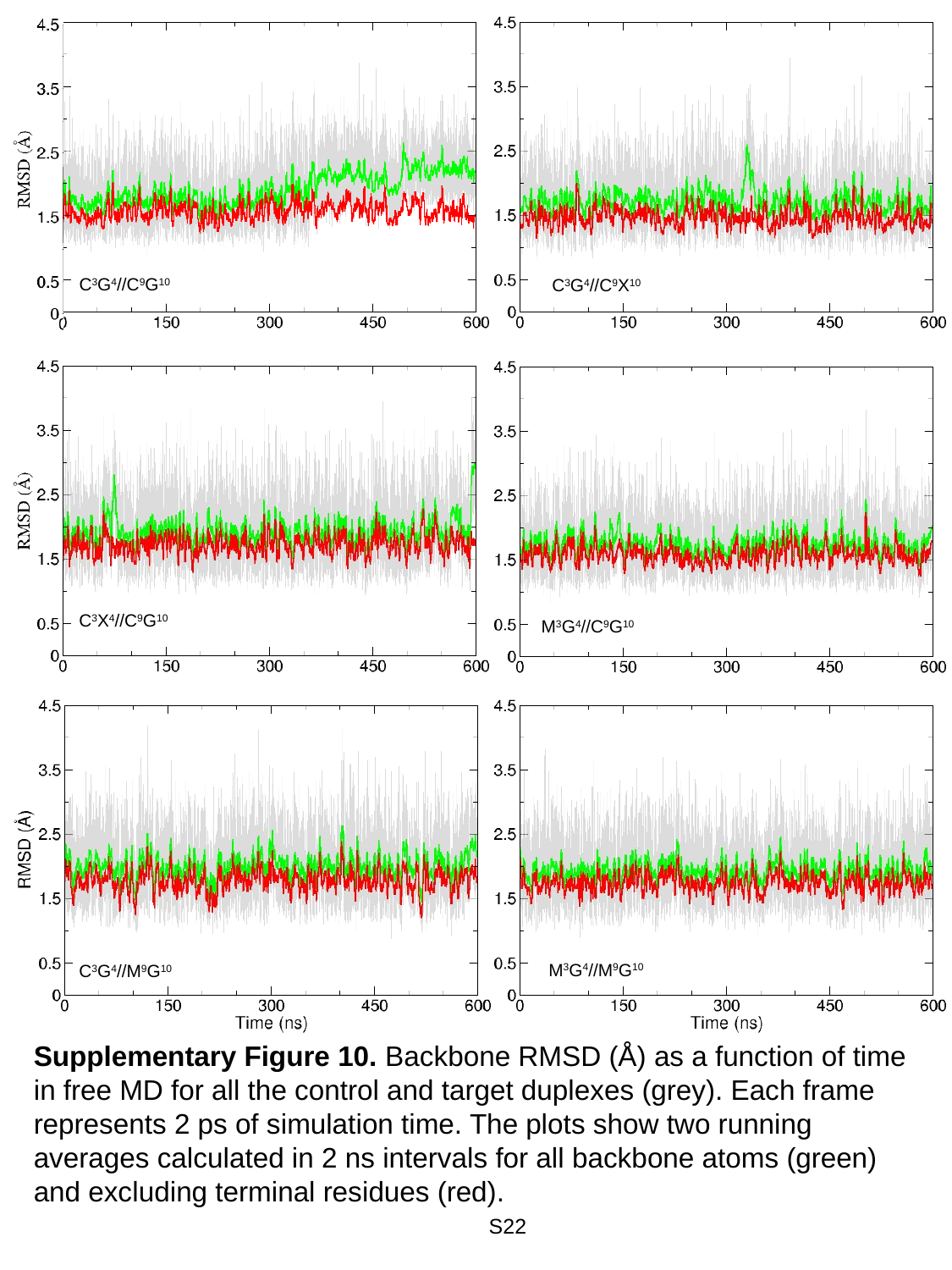

C3G4//C9G10
C3G4//C9X10
C3X4//C9G10
M3G4//C9G10
M3G4//M9G10
C3G4//M9G10
Supplementary Figure 10. Backbone RMSD (Å) as a function of time in free MD for all the control and target duplexes (grey). Each frame represents 2 ps of simulation time. The plots show two running averages calculated in 2 ns intervals for all backbone atoms (green) and excluding terminal residues (red).
S22

## Slide 23
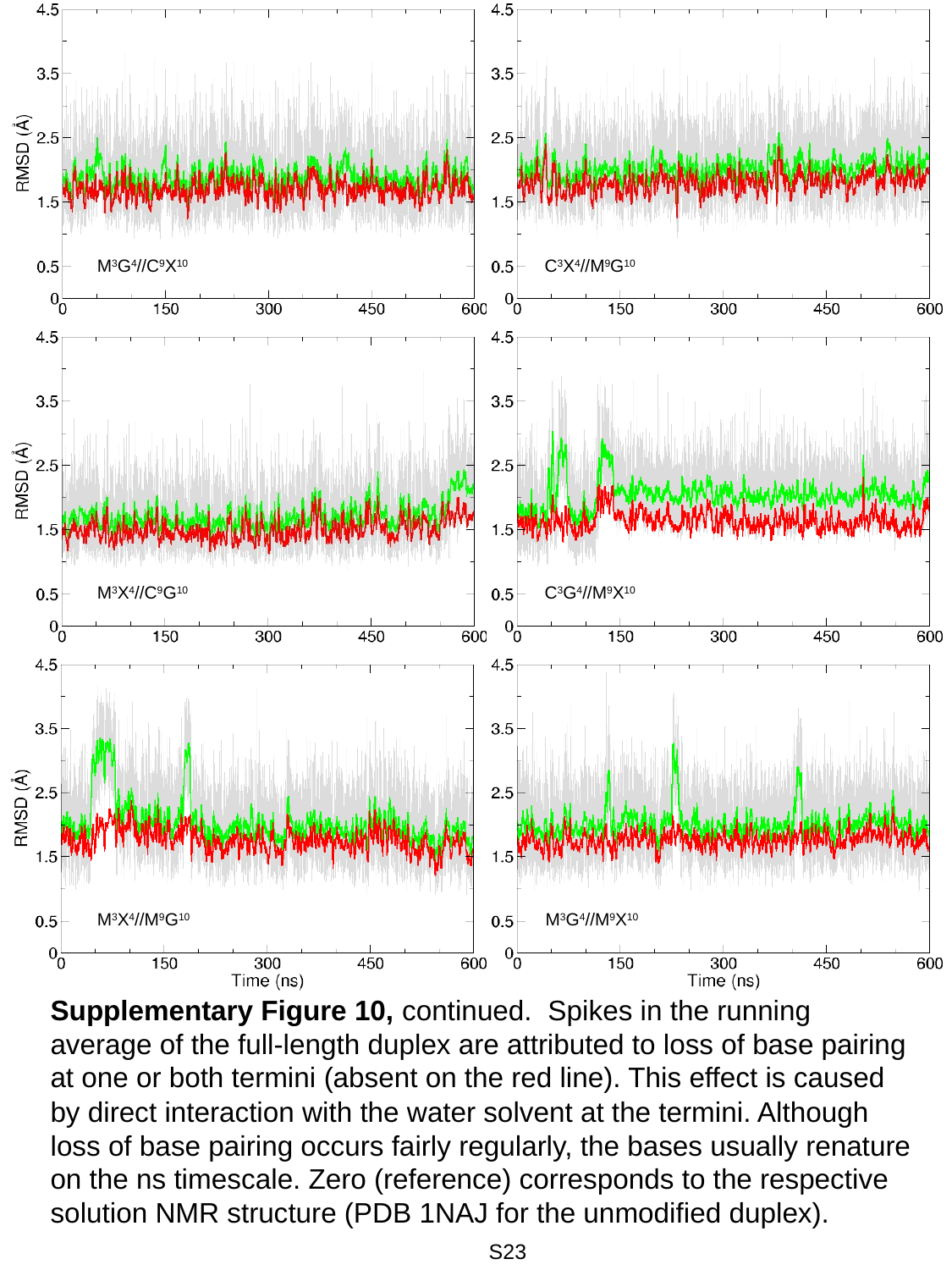

C3X4//M9G10
M3G4//C9X10
M3X4//C9G10
C3G4//M9X10
M3G4//M9X10
M3X4//M9G10
Supplementary Figure 10, continued. Spikes in the running average of the full-length duplex are attributed to loss of base pairing at one or both termini (absent on the red line). This effect is caused by direct interaction with the water solvent at the termini. Although loss of base pairing occurs fairly regularly, the bases usually renature on the ns timescale. Zero (reference) corresponds to the respective solution NMR structure (PDB 1NAJ for the unmodified duplex).
S23

## Slide 24
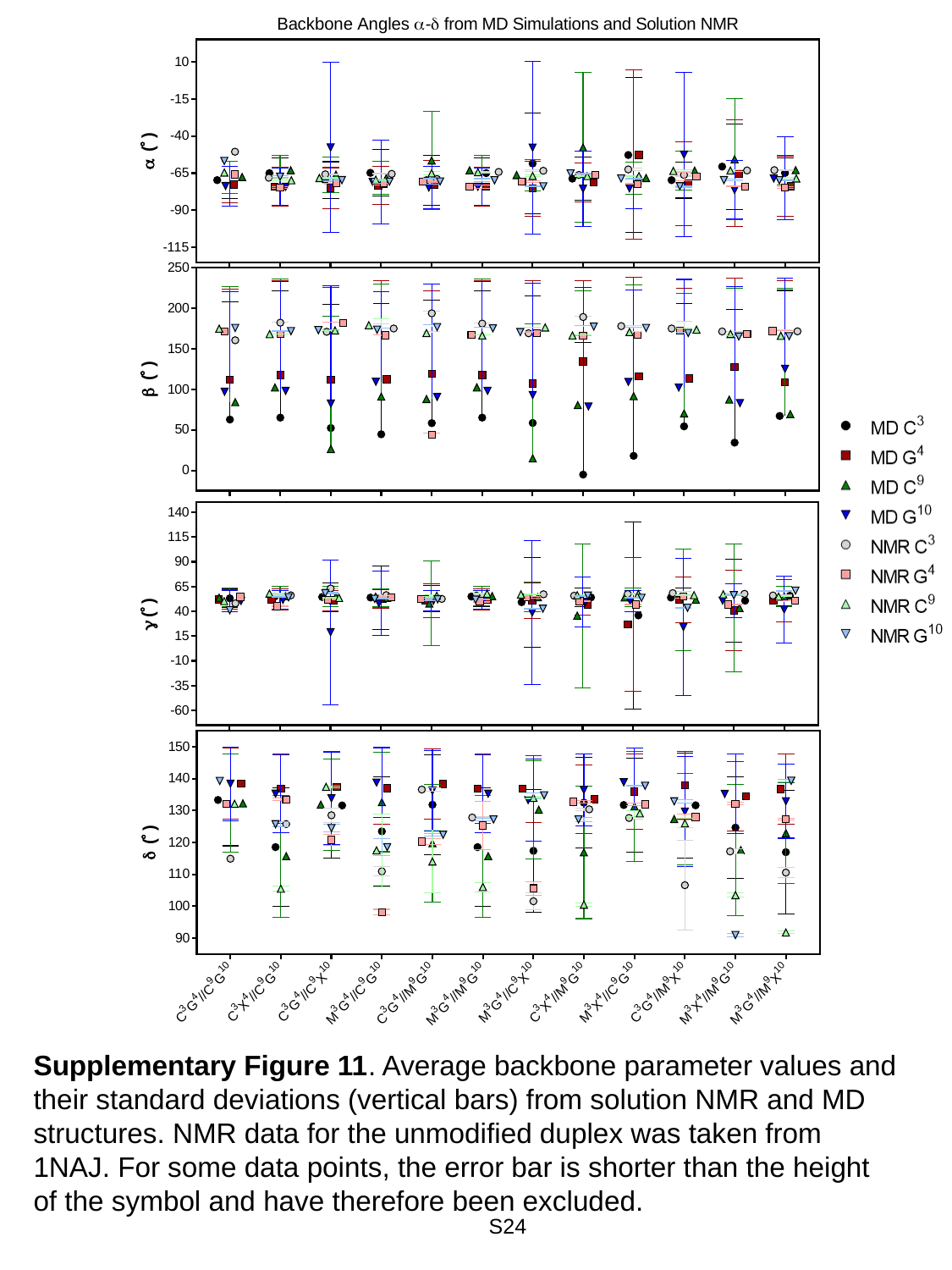

Supplementary Figure 11. Average backbone parameter values and their standard deviations (vertical bars) from solution NMR and MD structures. NMR data for the unmodified duplex was taken from 1NAJ. For some data points, the error bar is shorter than the height of the symbol and have therefore been excluded.
S24

## Slide 25
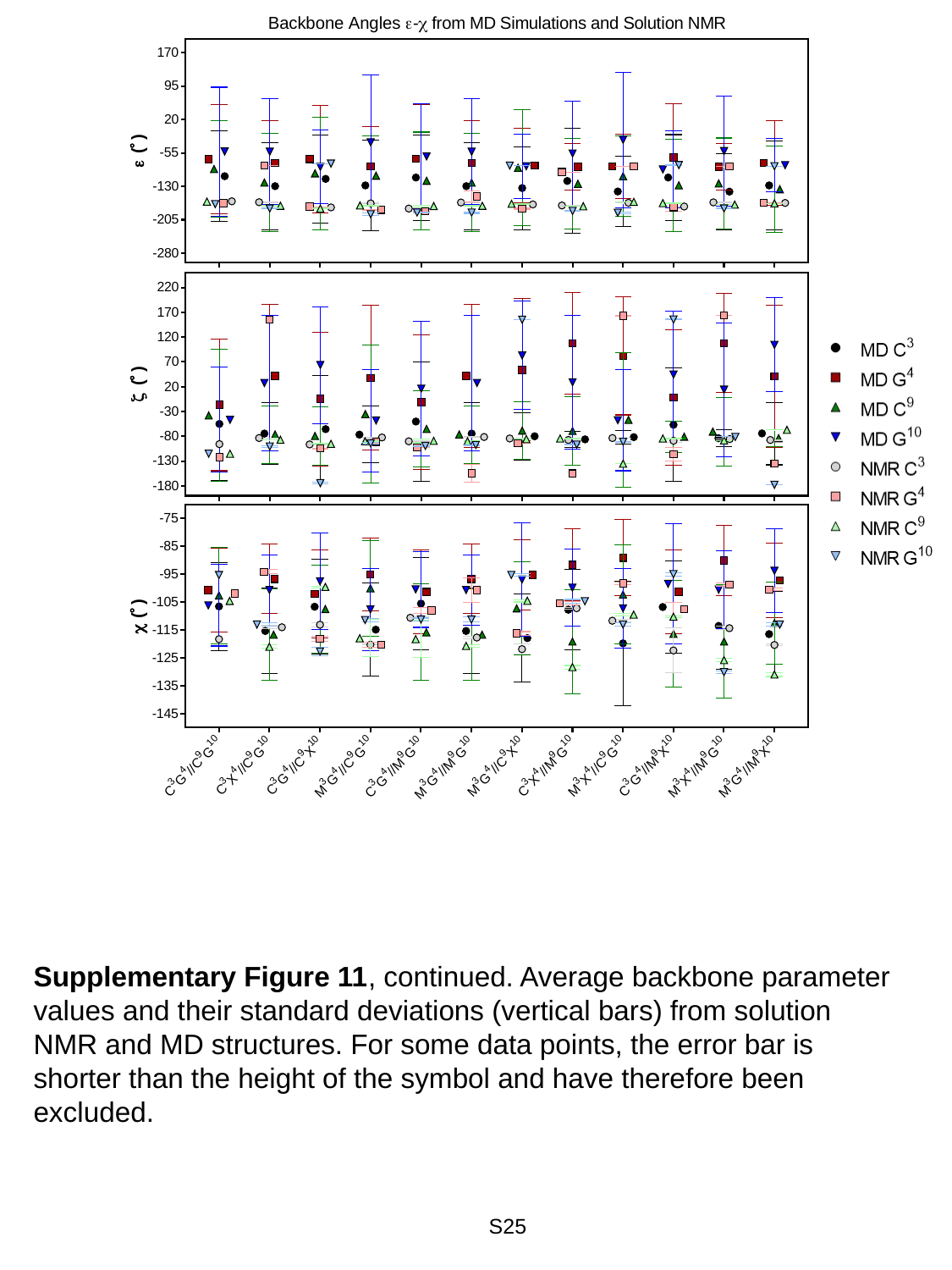

Supplementary Figure 11, continued. Average backbone parameter values and their standard deviations (vertical bars) from solution NMR and MD structures. For some data points, the error bar is shorter than the height of the symbol and have therefore been excluded.
S25

## Slide 26
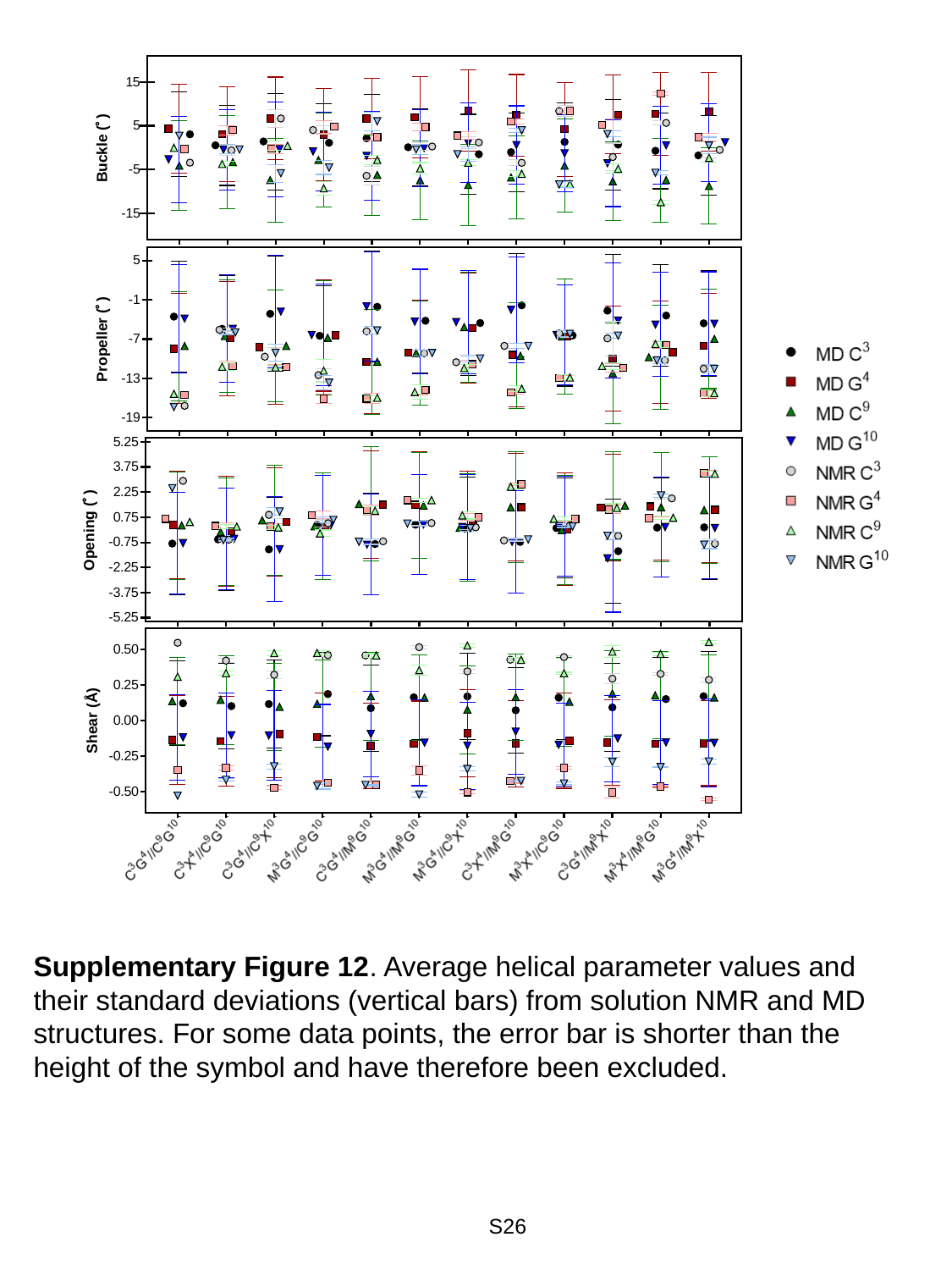

Supplementary Figure 12. Average helical parameter values and their standard deviations (vertical bars) from solution NMR and MD structures. For some data points, the error bar is shorter than the height of the symbol and have therefore been excluded.
S26

## Slide 27
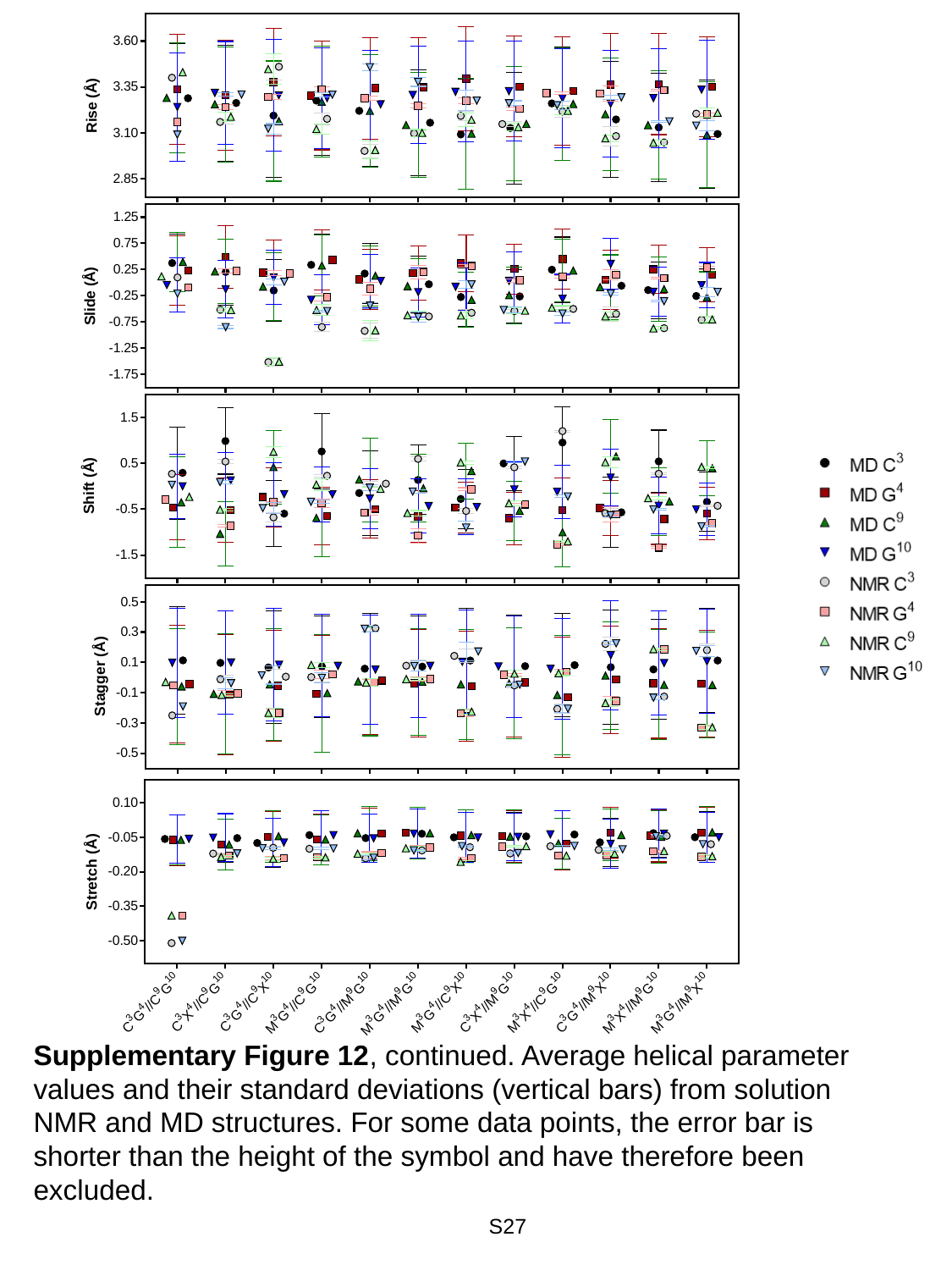

Supplementary Figure 12, continued. Average helical parameter values and their standard deviations (vertical bars) from solution NMR and MD structures. For some data points, the error bar is shorter than the height of the symbol and have therefore been excluded.
S27

## Slide 28
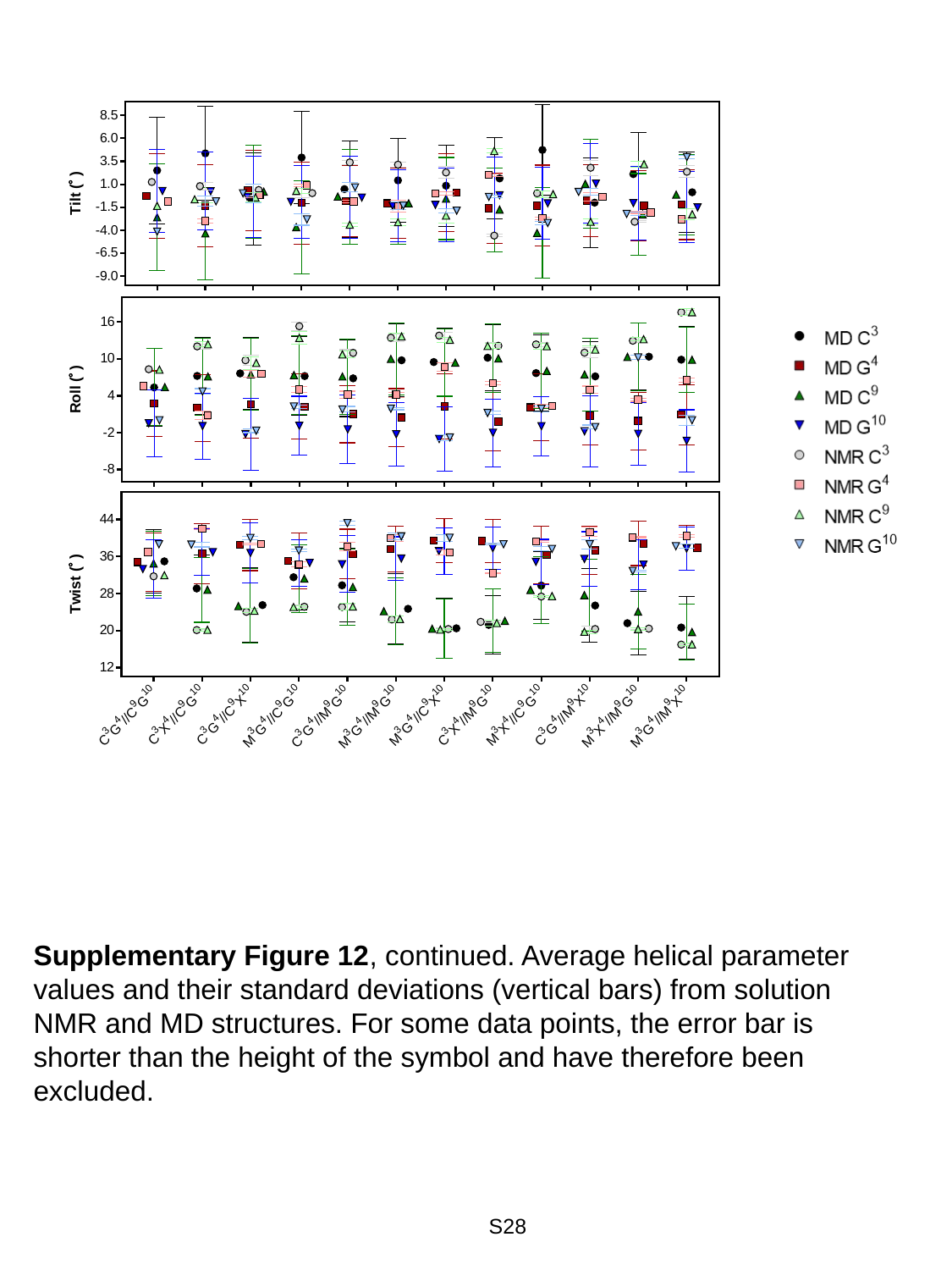

Supplementary Figure 12, continued. Average helical parameter values and their standard deviations (vertical bars) from solution NMR and MD structures. For some data points, the error bar is shorter than the height of the symbol and have therefore been excluded.
S28

## Slide 29
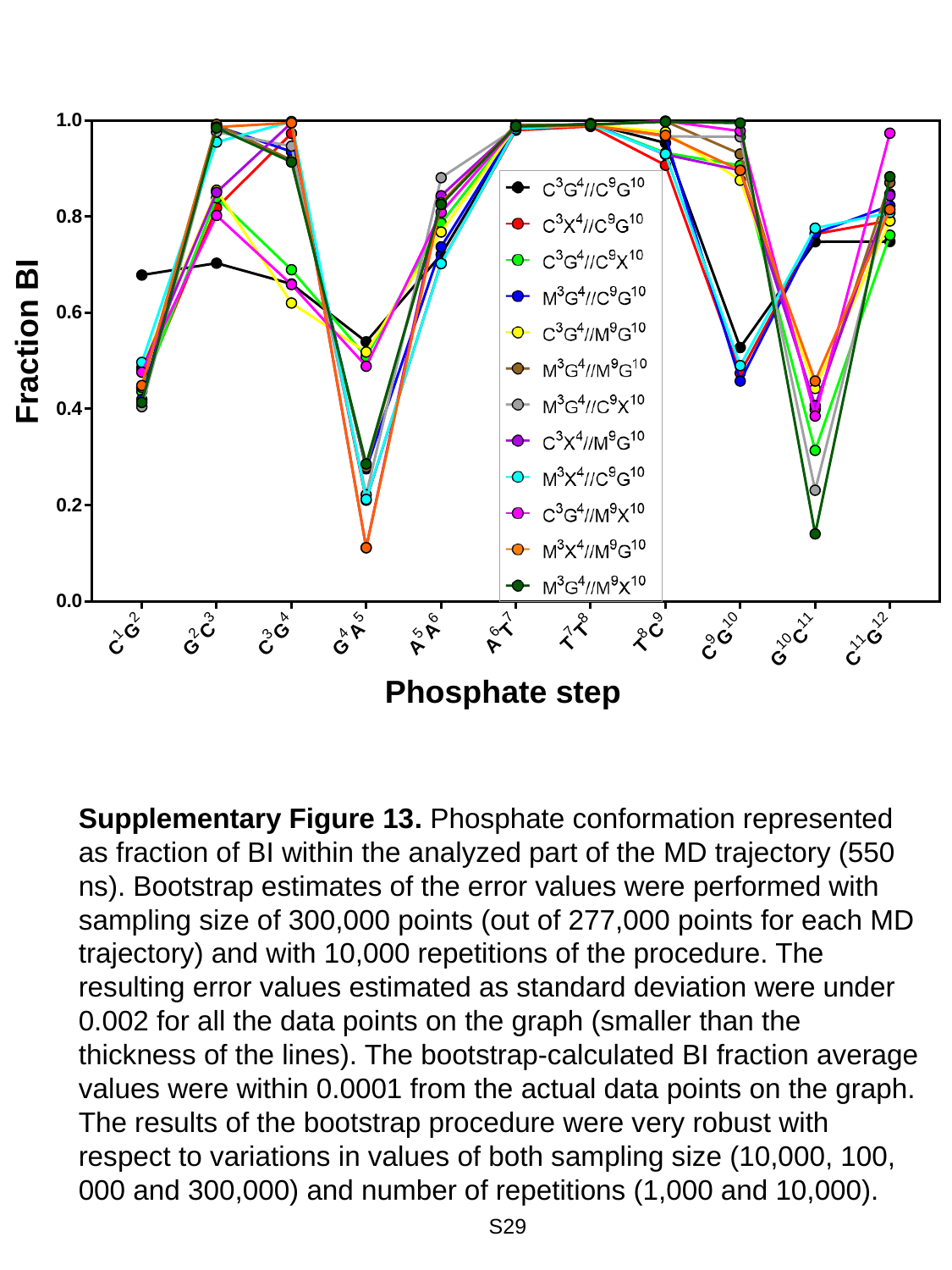

Fraction BI
Phosphate step
Supplementary Figure 13. Phosphate conformation represented as fraction of BI within the analyzed part of the MD trajectory (550 ns). Bootstrap estimates of the error values were performed with sampling size of 300,000 points (out of 277,000 points for each MD trajectory) and with 10,000 repetitions of the procedure. The resulting error values estimated as standard deviation were under 0.002 for all the data points on the graph (smaller than the thickness of the lines). The bootstrap-calculated BI fraction average values were within 0.0001 from the actual data points on the graph. The results of the bootstrap procedure were very robust with respect to variations in values of both sampling size (10,000, 100, 000 and 300,000) and number of repetitions (1,000 and 10,000).
S29

## Slide 30
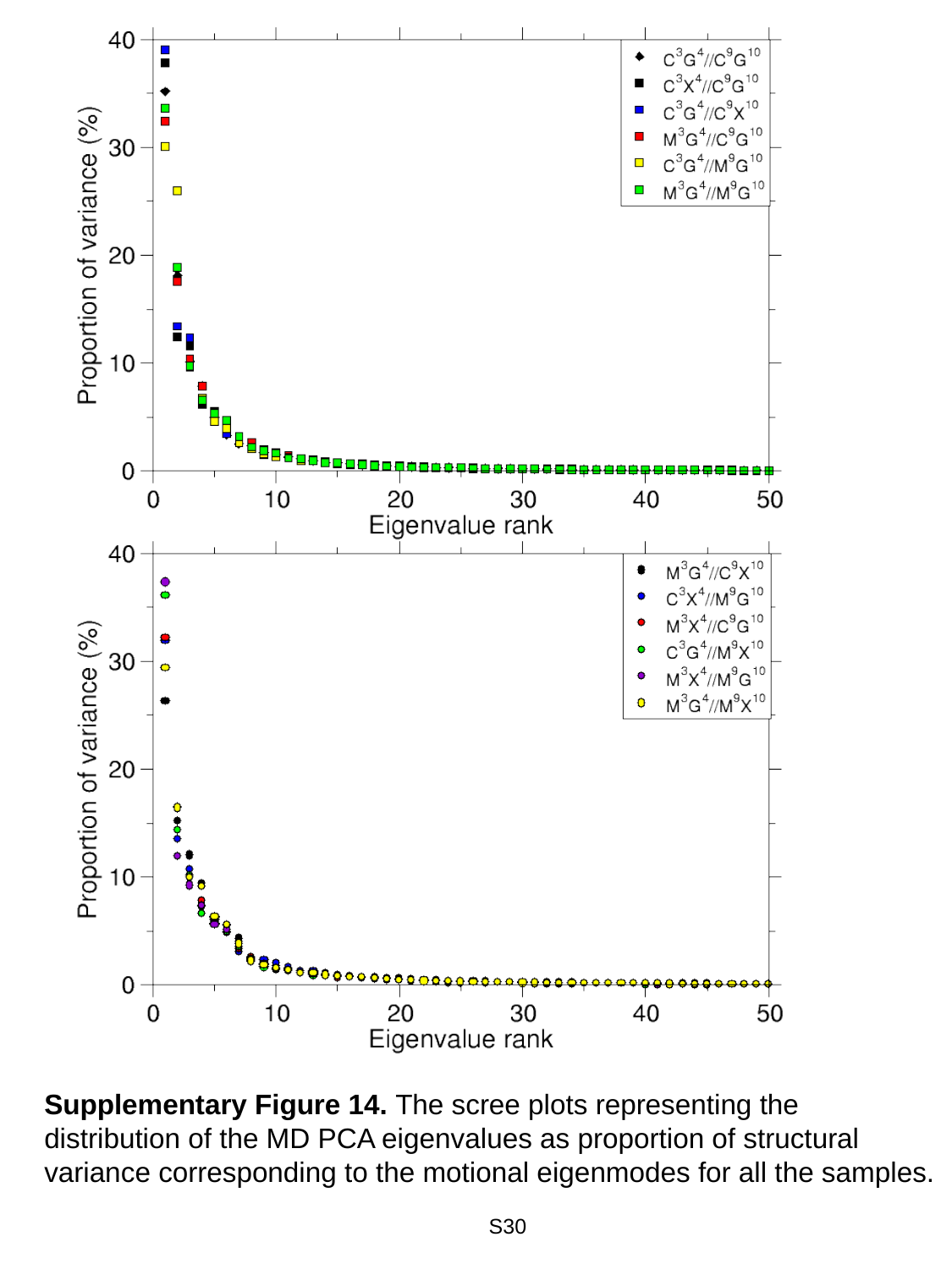

Supplementary Figure 14. The scree plots representing the distribution of the MD PCA eigenvalues as proportion of structural variance corresponding to the motional eigenmodes for all the samples.
S30

## Slide 31
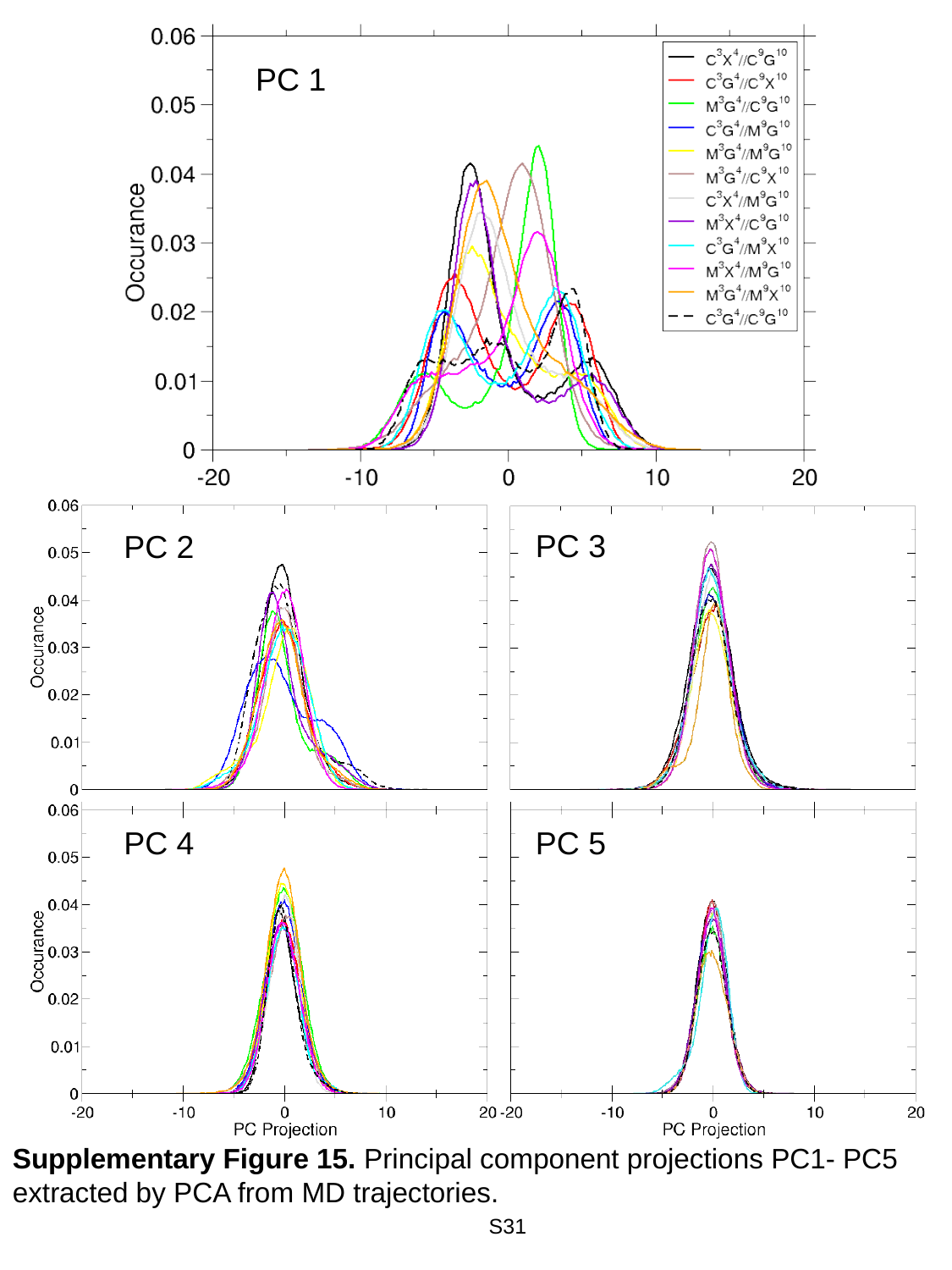

PC 1
PC 3
PC 2
PC 4
PC 5
Supplementary Figure 15. Principal component projections PC1- PC5 extracted by PCA from MD trajectories.
S31

## Slide 32
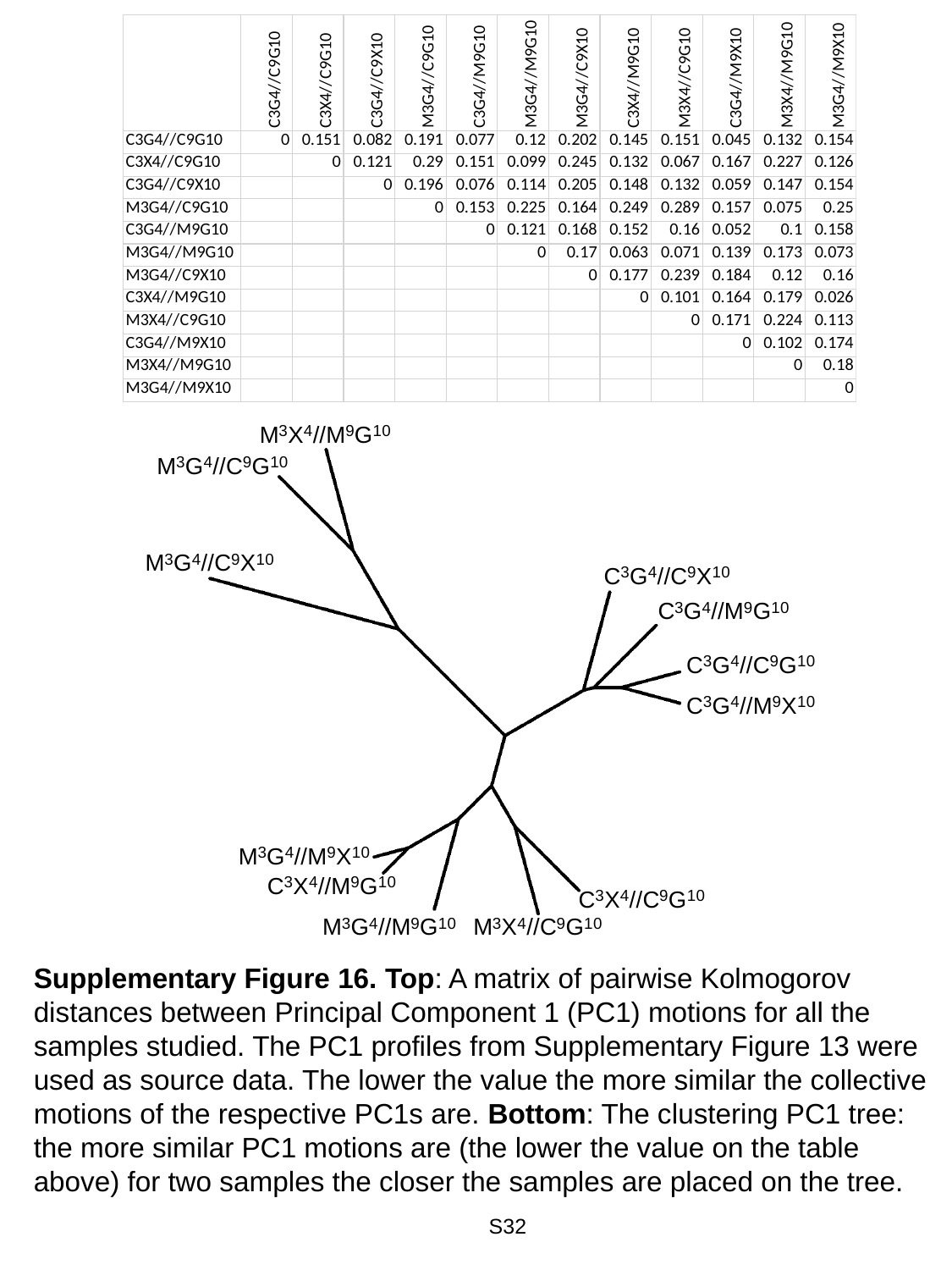

Supplementary Figure 16. Top: A matrix of pairwise Kolmogorov distances between Principal Component 1 (PC1) motions for all the samples studied. The PC1 profiles from Supplementary Figure 13 were used as source data. The lower the value the more similar the collective motions of the respective PC1s are. Bottom: The clustering PC1 tree: the more similar PC1 motions are (the lower the value on the table above) for two samples the closer the samples are placed on the tree.
S32

## Slide 33
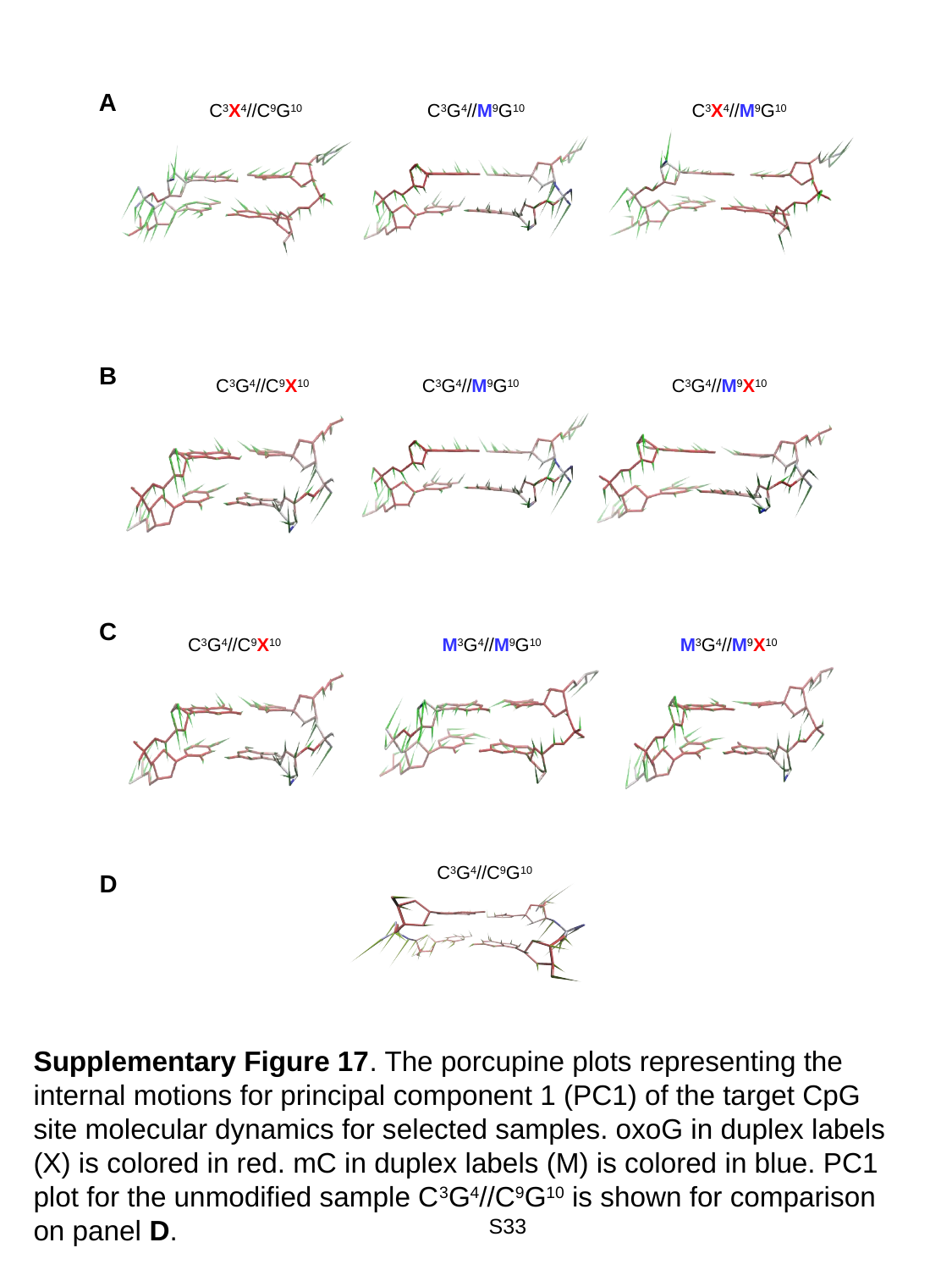

A
C3X4//C9G10
C3G4//M9G10
C3X4//M9G10
B
C3G4//C9X10
C3G4//M9G10
C3G4//M9X10
C
C3G4//C9X10
M3G4//M9G10
M3G4//M9X10
C3G4//C9G10
D
Supplementary Figure 17. The porcupine plots representing the internal motions for principal component 1 (PC1) of the target CpG site molecular dynamics for selected samples. oxoG in duplex labels (X) is colored in red. mC in duplex labels (M) is colored in blue. PC1 plot for the unmodified sample C3G4//C9G10 is shown for comparison on panel D.
S33

## Slide 34
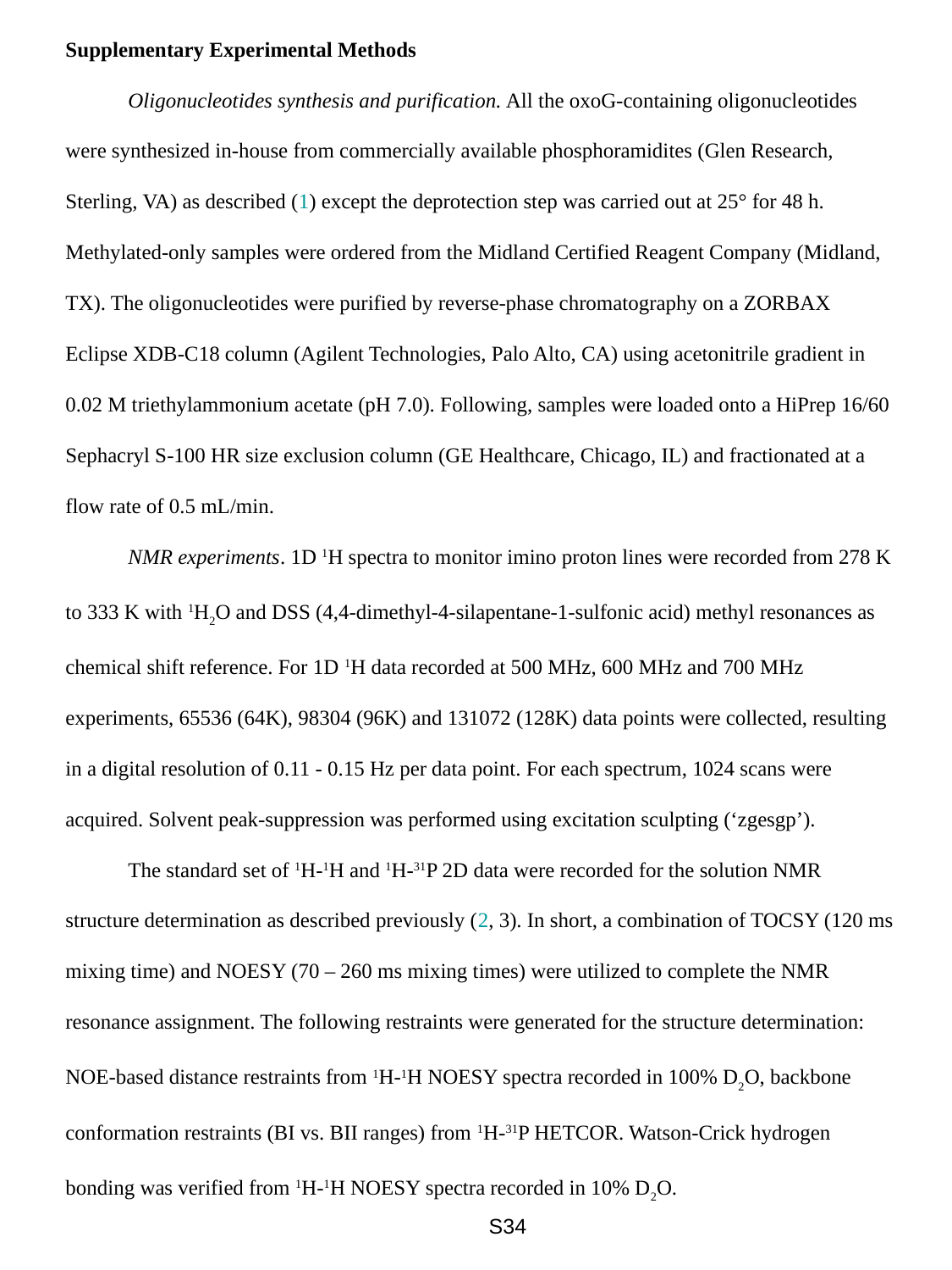

Supplementary Experimental Methods
Oligonucleotides synthesis and purification. All the oxoG-containing oligonucleotides were synthesized in-house from commercially available phosphoramidites (Glen Research, Sterling, VA) as described (1) except the deprotection step was carried out at 25° for 48 h. Methylated-only samples were ordered from the Midland Certified Reagent Company (Midland, TX). The oligonucleotides were purified by reverse-phase chromatography on a ZORBAX Eclipse XDB-C18 column (Agilent Technologies, Palo Alto, CA) using acetonitrile gradient in 0.02 M triethylammonium acetate (pH 7.0). Following, samples were loaded onto a HiPrep 16/60 Sephacryl S-100 HR size exclusion column (GE Healthcare, Chicago, IL) and fractionated at a flow rate of 0.5 mL/min.
NMR experiments. 1D 1H spectra to monitor imino proton lines were recorded from 278 K to 333 K with 1H2O and DSS (4,4-dimethyl-4-silapentane-1-sulfonic acid) methyl resonances as chemical shift reference. For 1D 1H data recorded at 500 MHz, 600 MHz and 700 MHz experiments, 65536 (64K), 98304 (96K) and 131072 (128K) data points were collected, resulting in a digital resolution of 0.11 - 0.15 Hz per data point. For each spectrum, 1024 scans were acquired. Solvent peak-suppression was performed using excitation sculpting (‘zgesgp’).
The standard set of 1H-1H and 1H-31P 2D data were recorded for the solution NMR structure determination as described previously (2, 3). In short, a combination of TOCSY (120 ms mixing time) and NOESY (70 – 260 ms mixing times) were utilized to complete the NMR resonance assignment. The following restraints were generated for the structure determination: NOE-based distance restraints from 1H-1H NOESY spectra recorded in 100% D2O, backbone conformation restraints (BI vs. BII ranges) from 1H-31P HETCOR. Watson-Crick hydrogen bonding was verified from 1H-1H NOESY spectra recorded in 10% D2O.
S34

## Slide 35
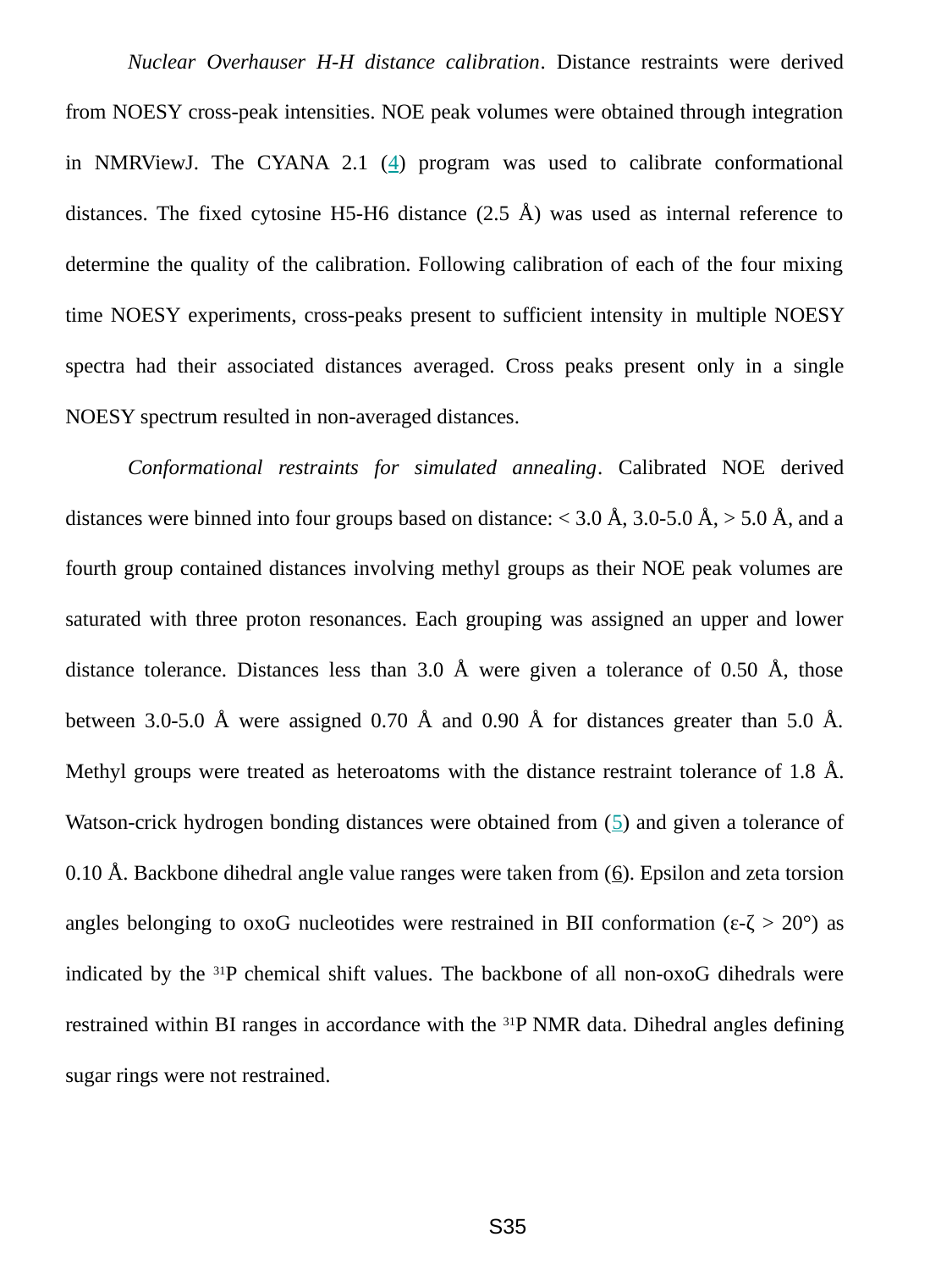

Nuclear Overhauser H-H distance calibration. Distance restraints were derived from NOESY cross-peak intensities. NOE peak volumes were obtained through integration in NMRViewJ. The CYANA 2.1 (4) program was used to calibrate conformational distances. The fixed cytosine H5-H6 distance (2.5 Å) was used as internal reference to determine the quality of the calibration. Following calibration of each of the four mixing time NOESY experiments, cross-peaks present to sufficient intensity in multiple NOESY spectra had their associated distances averaged. Cross peaks present only in a single NOESY spectrum resulted in non-averaged distances.
Conformational restraints for simulated annealing. Calibrated NOE derived distances were binned into four groups based on distance: < 3.0 Å, 3.0-5.0 Å, > 5.0 Å, and a fourth group contained distances involving methyl groups as their NOE peak volumes are saturated with three proton resonances. Each grouping was assigned an upper and lower distance tolerance. Distances less than 3.0 Å were given a tolerance of 0.50 Å, those between 3.0-5.0 Å were assigned 0.70 Å and 0.90 Å for distances greater than 5.0 Å. Methyl groups were treated as heteroatoms with the distance restraint tolerance of 1.8 Å. Watson-crick hydrogen bonding distances were obtained from (5) and given a tolerance of 0.10 Å. Backbone dihedral angle value ranges were taken from (6). Epsilon and zeta torsion angles belonging to oxoG nucleotides were restrained in BII conformation (ε-ζ > 20°) as indicated by the 31P chemical shift values. The backbone of all non-oxoG dihedrals were restrained within BI ranges in accordance with the 31P NMR data. Dihedral angles defining sugar rings were not restrained.
S35

## Slide 36
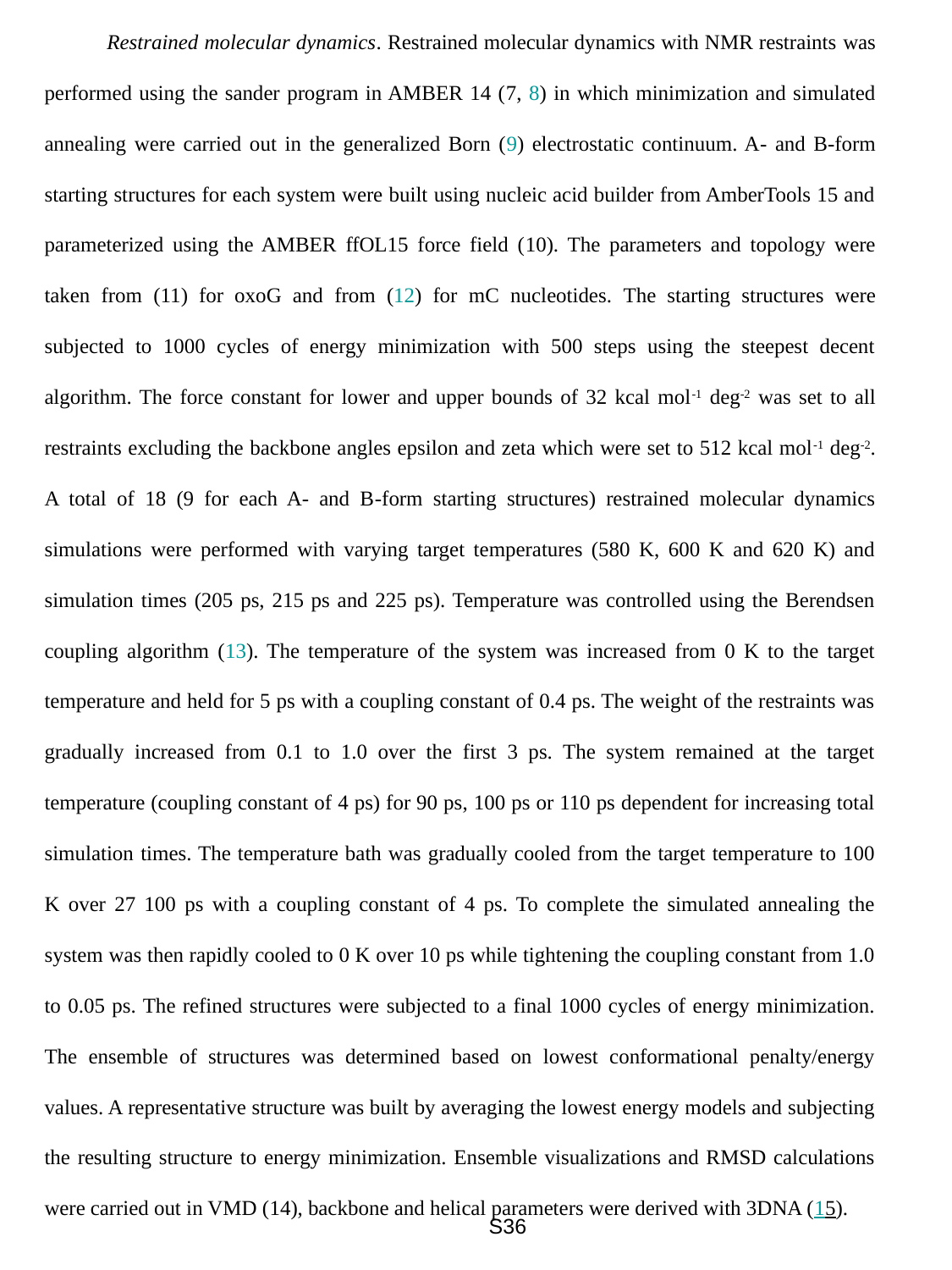

Restrained molecular dynamics. Restrained molecular dynamics with NMR restraints was performed using the sander program in AMBER 14 (7, 8) in which minimization and simulated annealing were carried out in the generalized Born (9) electrostatic continuum. A- and B-form starting structures for each system were built using nucleic acid builder from AmberTools 15 and parameterized using the AMBER ffOL15 force field (10). The parameters and topology were taken from (11) for oxoG and from (12) for mC nucleotides. The starting structures were subjected to 1000 cycles of energy minimization with 500 steps using the steepest decent algorithm. The force constant for lower and upper bounds of 32 kcal mol-1 deg-2 was set to all restraints excluding the backbone angles epsilon and zeta which were set to 512 kcal mol-1 deg-2. A total of 18 (9 for each A- and B-form starting structures) restrained molecular dynamics simulations were performed with varying target temperatures (580 K, 600 K and 620 K) and simulation times (205 ps, 215 ps and 225 ps). Temperature was controlled using the Berendsen coupling algorithm (13). The temperature of the system was increased from 0 K to the target temperature and held for 5 ps with a coupling constant of 0.4 ps. The weight of the restraints was gradually increased from 0.1 to 1.0 over the first 3 ps. The system remained at the target temperature (coupling constant of 4 ps) for 90 ps, 100 ps or 110 ps dependent for increasing total simulation times. The temperature bath was gradually cooled from the target temperature to 100 K over 27 100 ps with a coupling constant of 4 ps. To complete the simulated annealing the system was then rapidly cooled to 0 K over 10 ps while tightening the coupling constant from 1.0 to 0.05 ps. The refined structures were subjected to a final 1000 cycles of energy minimization. The ensemble of structures was determined based on lowest conformational penalty/energy values. A representative structure was built by averaging the lowest energy models and subjecting the resulting structure to energy minimization. Ensemble visualizations and RMSD calculations were carried out in VMD (14), backbone and helical parameters were derived with 3DNA (15).
S36

## Slide 37
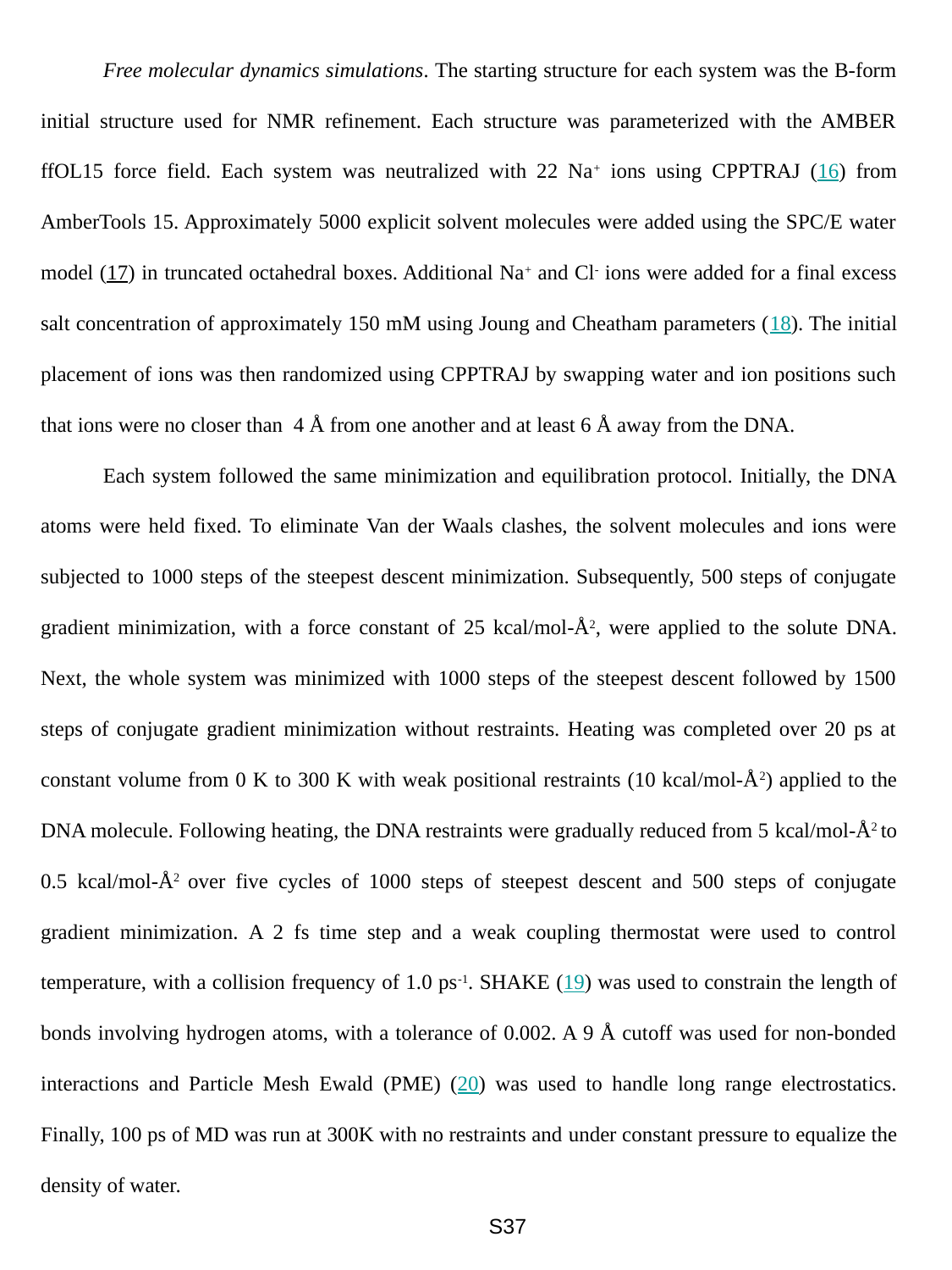

Free molecular dynamics simulations. The starting structure for each system was the B-form initial structure used for NMR refinement. Each structure was parameterized with the AMBER ffOL15 force field. Each system was neutralized with 22 Na+ ions using CPPTRAJ (16) from AmberTools 15. Approximately 5000 explicit solvent molecules were added using the SPC/E water model (17) in truncated octahedral boxes. Additional Na+ and Cl- ions were added for a final excess salt concentration of approximately 150 mM using Joung and Cheatham parameters (18). The initial placement of ions was then randomized using CPPTRAJ by swapping water and ion positions such that ions were no closer than 4 Å from one another and at least 6 Å away from the DNA.
Each system followed the same minimization and equilibration protocol. Initially, the DNA atoms were held fixed. To eliminate Van der Waals clashes, the solvent molecules and ions were subjected to 1000 steps of the steepest descent minimization. Subsequently, 500 steps of conjugate gradient minimization, with a force constant of 25 kcal/mol-Å2, were applied to the solute DNA. Next, the whole system was minimized with 1000 steps of the steepest descent followed by 1500 steps of conjugate gradient minimization without restraints. Heating was completed over 20 ps at constant volume from 0 K to 300 K with weak positional restraints (10 kcal/mol-Å2) applied to the DNA molecule. Following heating, the DNA restraints were gradually reduced from 5 kcal/mol-Å2 to 0.5 kcal/mol-Å2 over five cycles of 1000 steps of steepest descent and 500 steps of conjugate gradient minimization. A 2 fs time step and a weak coupling thermostat were used to control temperature, with a collision frequency of 1.0 ps-1. SHAKE (19) was used to constrain the length of bonds involving hydrogen atoms, with a tolerance of 0.002. A 9 Å cutoff was used for non-bonded interactions and Particle Mesh Ewald (PME) (20) was used to handle long range electrostatics. Finally, 100 ps of MD was run at 300K with no restraints and under constant pressure to equalize the density of water.
S37

## Slide 38
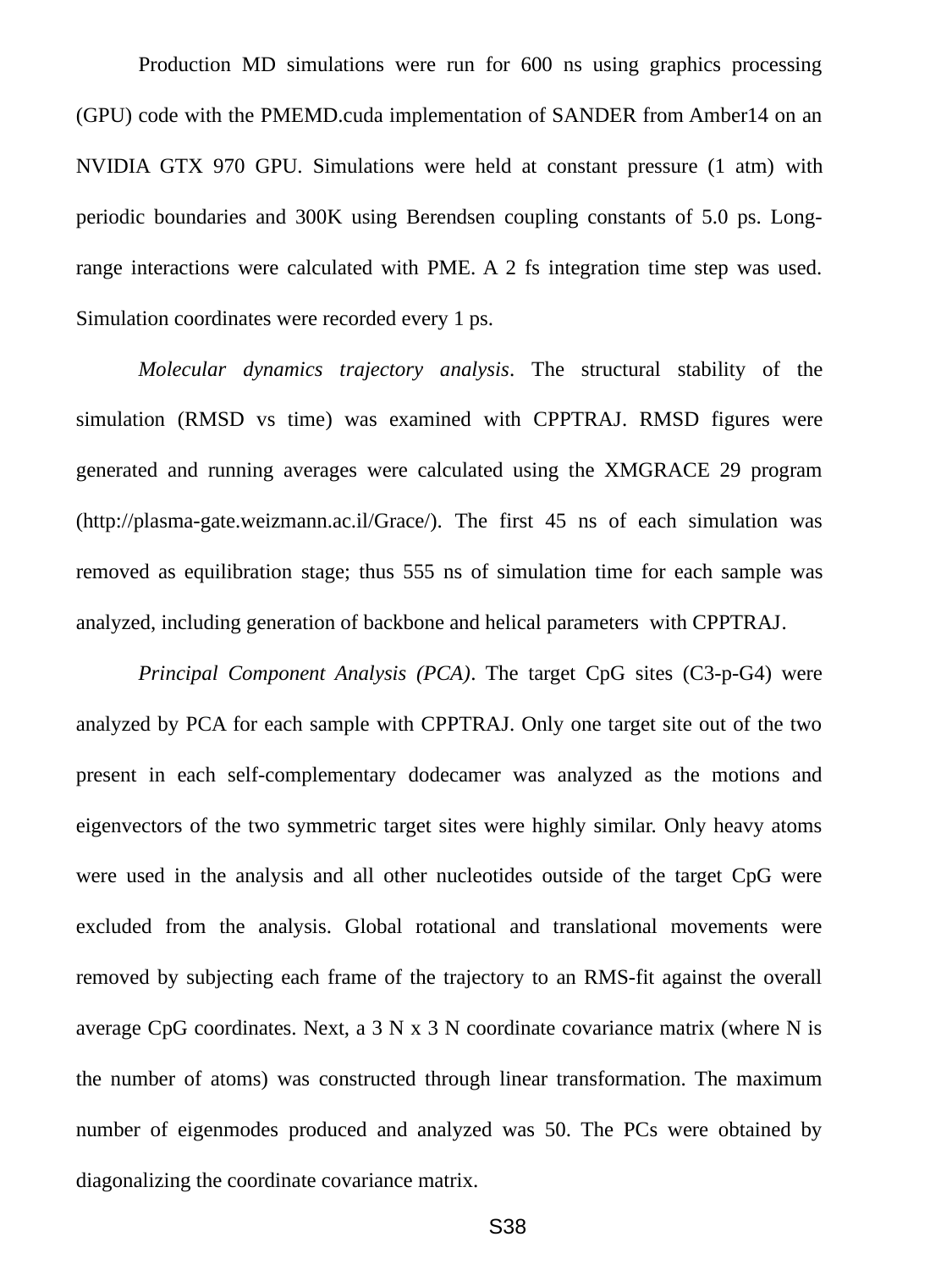

Production MD simulations were run for 600 ns using graphics processing (GPU) code with the PMEMD.cuda implementation of SANDER from Amber14 on an NVIDIA GTX 970 GPU. Simulations were held at constant pressure (1 atm) with periodic boundaries and 300K using Berendsen coupling constants of 5.0 ps. Long-range interactions were calculated with PME. A 2 fs integration time step was used. Simulation coordinates were recorded every 1 ps.
Molecular dynamics trajectory analysis. The structural stability of the simulation (RMSD vs time) was examined with CPPTRAJ. RMSD figures were generated and running averages were calculated using the XMGRACE 29 program (http://plasma-gate.weizmann.ac.il/Grace/). The first 45 ns of each simulation was removed as equilibration stage; thus 555 ns of simulation time for each sample was analyzed, including generation of backbone and helical parameters with CPPTRAJ.
Principal Component Analysis (PCA). The target CpG sites (C3-p-G4) were analyzed by PCA for each sample with CPPTRAJ. Only one target site out of the two present in each self-complementary dodecamer was analyzed as the motions and eigenvectors of the two symmetric target sites were highly similar. Only heavy atoms were used in the analysis and all other nucleotides outside of the target CpG were excluded from the analysis. Global rotational and translational movements were removed by subjecting each frame of the trajectory to an RMS-fit against the overall average CpG coordinates. Next, a 3 N x 3 N coordinate covariance matrix (where N is the number of atoms) was constructed through linear transformation. The maximum number of eigenmodes produced and analyzed was 50. The PCs were obtained by diagonalizing the coordinate covariance matrix.
S38

## Slide 39
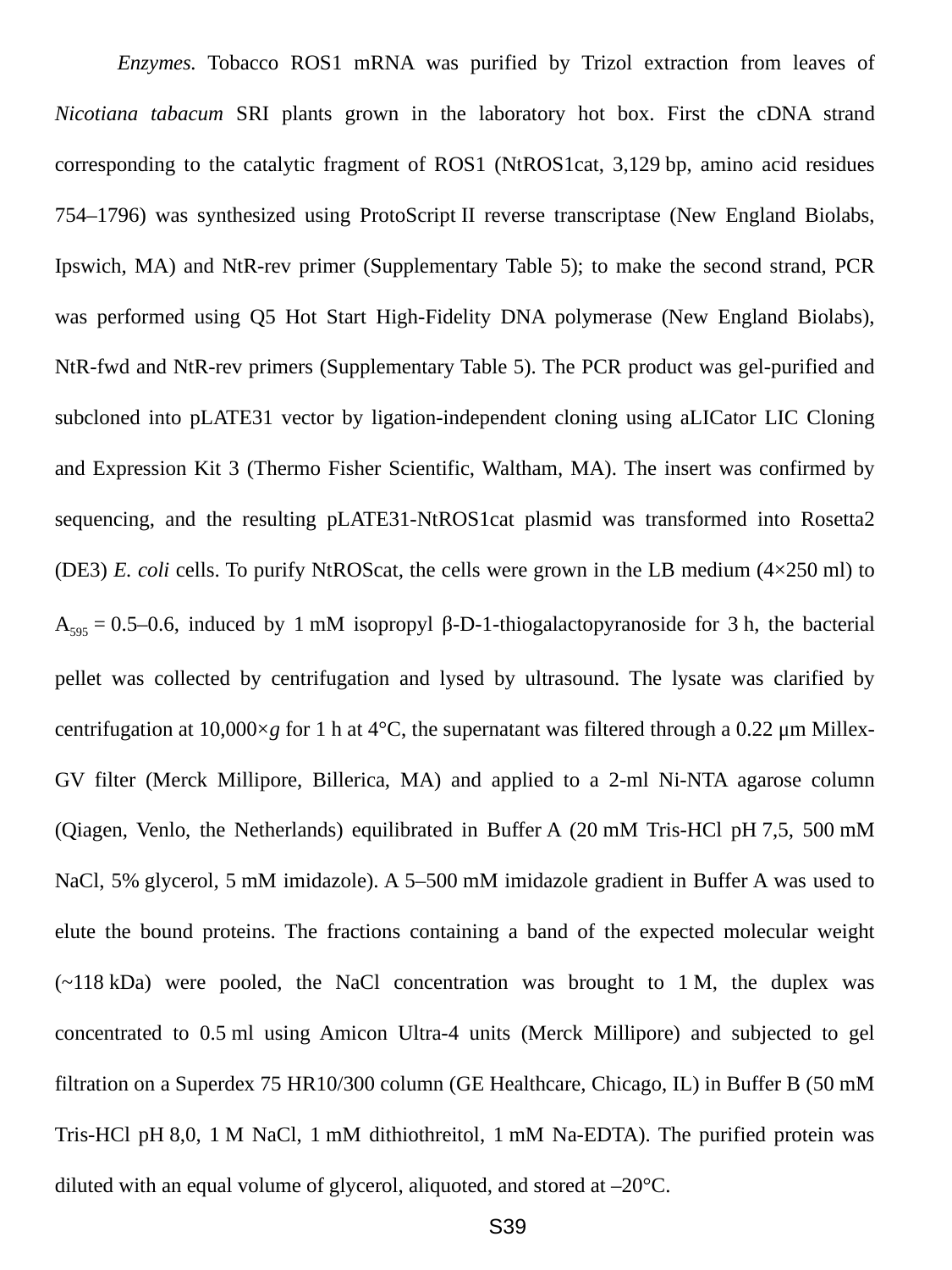

Enzymes. Tobacco ROS1 mRNA was purified by Trizol extraction from leaves of Nicotiana tabacum SRI plants grown in the laboratory hot box. First the cDNA strand corresponding to the catalytic fragment of ROS1 (NtROS1cat, 3,129 bp, amino acid residues 754–1796) was synthesized using ProtoScript II reverse transcriptase (New England Biolabs, Ipswich, MA) and NtR-rev primer (Supplementary Table 5); to make the second strand, PCR was performed using Q5 Hot Start High-Fidelity DNA polymerase (New England Biolabs), NtR-fwd and NtR-rev primers (Supplementary Table 5). The PCR product was gel-purified and subcloned into pLATE31 vector by ligation-independent cloning using aLICator LIC Cloning and Expression Kit 3 (Thermo Fisher Scientific, Waltham, MA). The insert was confirmed by sequencing, and the resulting pLATE31-NtROS1cat plasmid was transformed into Rosetta2 (DE3) E. coli cells. To purify NtROScat, the cells were grown in the LB medium (4×250 ml) to А595 = 0.5–0.6, induced by 1 mM isopropyl β-D-1-thiogalactopyranoside for 3 h, the bacterial pellet was collected by centrifugation and lysed by ultrasound. The lysate was clarified by centrifugation at 10,000×g for 1 h at 4°C, the supernatant was filtered through a 0.22 μm Millex-GV filter (Merck Millipore, Billerica, MA) and applied to a 2-ml Ni-NTA agarose column (Qiagen, Venlo, the Netherlands) equilibrated in Buffer A (20 mM Tris-HCl pH 7,5, 500 mM NaCl, 5% glycerol, 5 mM imidazole). A 5–500 mM imidazole gradient in Buffer A was used to elute the bound proteins. The fractions containing a band of the expected molecular weight (~118 kDa) were pooled, the NaCl concentration was brought to 1 M, the duplex was concentrated to 0.5 ml using Amicon Ultra-4 units (Merck Millipore) and subjected to gel filtration on a Superdex 75 HR10/300 column (GE Healthcare, Chicago, IL) in Buffer B (50 mM Tris-HCl pH 8,0, 1 M NaCl, 1 mM dithiothreitol, 1 mM Na-EDTA). The purified protein was diluted with an equal volume of glycerol, aliquoted, and stored at –20°C.
S39

## Slide 40
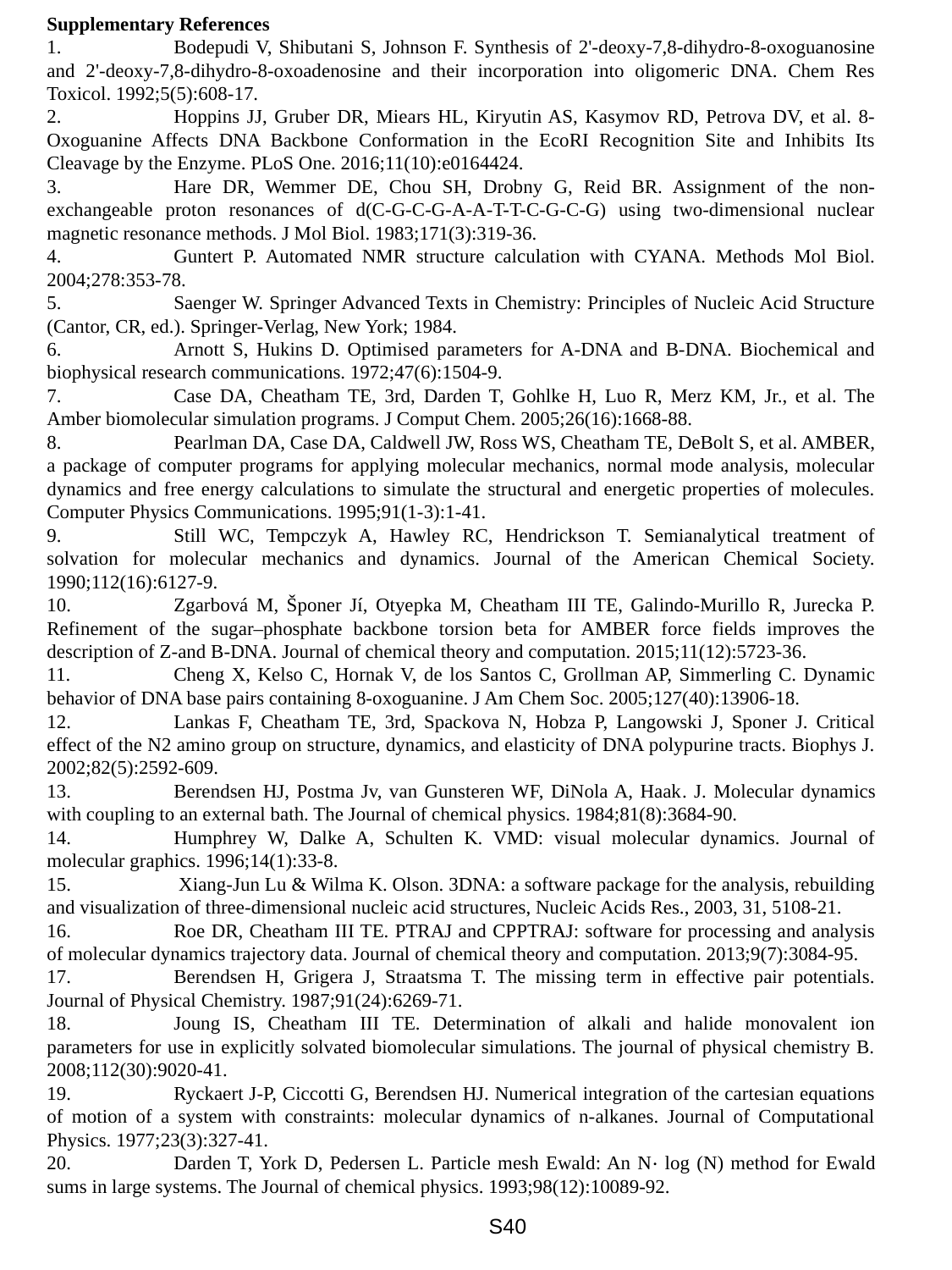

Supplementary References
1.	Bodepudi V, Shibutani S, Johnson F. Synthesis of 2'-deoxy-7,8-dihydro-8-oxoguanosine and 2'-deoxy-7,8-dihydro-8-oxoadenosine and their incorporation into oligomeric DNA. Chem Res Toxicol. 1992;5(5):608-17.
2.	Hoppins JJ, Gruber DR, Miears HL, Kiryutin AS, Kasymov RD, Petrova DV, et al. 8-Oxoguanine Affects DNA Backbone Conformation in the EcoRI Recognition Site and Inhibits Its Cleavage by the Enzyme. PLoS One. 2016;11(10):e0164424.
3.	Hare DR, Wemmer DE, Chou SH, Drobny G, Reid BR. Assignment of the non-exchangeable proton resonances of d(C-G-C-G-A-A-T-T-C-G-C-G) using two-dimensional nuclear magnetic resonance methods. J Mol Biol. 1983;171(3):319-36.
4.	Guntert P. Automated NMR structure calculation with CYANA. Methods Mol Biol. 2004;278:353-78.
5.	Saenger W. Springer Advanced Texts in Chemistry: Principles of Nucleic Acid Structure (Cantor, CR, ed.). Springer-Verlag, New York; 1984.
6.	Arnott S, Hukins D. Optimised parameters for A-DNA and B-DNA. Biochemical and biophysical research communications. 1972;47(6):1504-9.
7.	Case DA, Cheatham TE, 3rd, Darden T, Gohlke H, Luo R, Merz KM, Jr., et al. The Amber biomolecular simulation programs. J Comput Chem. 2005;26(16):1668-88.
8.	Pearlman DA, Case DA, Caldwell JW, Ross WS, Cheatham TE, DeBolt S, et al. AMBER, a package of computer programs for applying molecular mechanics, normal mode analysis, molecular dynamics and free energy calculations to simulate the structural and energetic properties of molecules. Computer Physics Communications. 1995;91(1-3):1-41.
9.	Still WC, Tempczyk A, Hawley RC, Hendrickson T. Semianalytical treatment of solvation for molecular mechanics and dynamics. Journal of the American Chemical Society. 1990;112(16):6127-9.
10.	Zgarbová M, Šponer Jí, Otyepka M, Cheatham III TE, Galindo-Murillo R, Jurecka P. Refinement of the sugar–phosphate backbone torsion beta for AMBER force fields improves the description of Z-and B-DNA. Journal of chemical theory and computation. 2015;11(12):5723-36.
11.	Cheng X, Kelso C, Hornak V, de los Santos C, Grollman AP, Simmerling C. Dynamic behavior of DNA base pairs containing 8-oxoguanine. J Am Chem Soc. 2005;127(40):13906-18.
12.	Lankas F, Cheatham TE, 3rd, Spackova N, Hobza P, Langowski J, Sponer J. Critical effect of the N2 amino group on structure, dynamics, and elasticity of DNA polypurine tracts. Biophys J. 2002;82(5):2592-609.
13.	Berendsen HJ, Postma Jv, van Gunsteren WF, DiNola A, Haak. J. Molecular dynamics with coupling to an external bath. The Journal of chemical physics. 1984;81(8):3684-90.
14.	Humphrey W, Dalke A, Schulten K. VMD: visual molecular dynamics. Journal of molecular graphics. 1996;14(1):33-8.
15.	 Xiang-Jun Lu & Wilma K. Olson. 3DNA: a software package for the analysis, rebuilding and visualization of three-dimensional nucleic acid structures, Nucleic Acids Res., 2003, 31, 5108-21.
16.	Roe DR, Cheatham III TE. PTRAJ and CPPTRAJ: software for processing and analysis of molecular dynamics trajectory data. Journal of chemical theory and computation. 2013;9(7):3084-95.
17.	Berendsen H, Grigera J, Straatsma T. The missing term in effective pair potentials. Journal of Physical Chemistry. 1987;91(24):6269-71.
18.	Joung IS, Cheatham III TE. Determination of alkali and halide monovalent ion parameters for use in explicitly solvated biomolecular simulations. The journal of physical chemistry B. 2008;112(30):9020-41.
19.	Ryckaert J-P, Ciccotti G, Berendsen HJ. Numerical integration of the cartesian equations of motion of a system with constraints: molecular dynamics of n-alkanes. Journal of Computational Physics. 1977;23(3):327-41.
20.	Darden T, York D, Pedersen L. Particle mesh Ewald: An N⋅ log (N) method for Ewald sums in large systems. The Journal of chemical physics. 1993;98(12):10089-92.
S40
